# Perspectives of inpatients with palliative care needs, their families, clinicians and key stakeholders on measuring quality of hospital care via patient experience measures: A qualitative study

**DOI:** 10.1177/02692163231209845

**Published:** 2023-11-03

**Authors:** Claudia Virdun, Elise Button, Jane L Phillips, Patsy Yates, Tim Luckett

**Affiliations:** 1 Cancer & Palliative Care Outcomes Centre, School of Nursing, Centre for Healthcare Transformation, Faculty of Health, Queensland University of Technology, Kelvin Grove, QLD, Australia; 2 Improving Palliative, Aged and Chronic Care through Clinical Research and Translation (IMPACCT), Faculty of Health, University of Technology Sydney (UTS), Sydney, NSW, Australia; 3 Healthcare Excellence and Innovation, Metro North Hospital and Health Services, Herston, QLD, Australia; 4 School of Nursing, Faculty of Health, Queensland University of Technology, Kelvin Grove, QLD, Australia

**Keywords:** Palliative care, hospital, patient reported experience measures, consumer participation, qualitative research, patient-centred care, quality improvement, quality of care

## Abstract

**Background:**

Globally there are high numbers of patients with palliative care needs receiving care in hospitals. Patient reported experience measures (PREMs) provide useful data to guide improvement work. How to implement PREMs within palliative care populations is unclear.

**Aim:**

To explore the perspectives of inpatients with palliative care needs, their family members, and the clinical team regarding the use of a generic PREM as compared with a PREM designed for people with palliative care needs and related implementation factors.

**Design:**

A qualitative study was undertaken using semi-structured interviews and focus groups and integrated thematic analysis.

**Setting/participants:**

Inpatients with palliative care needs, their family members, and clinical team members were recruited from three wards in an Australian metropolitan hospital.

**Results:**

Twenty-seven interviews and three focus groups were conducted. Six themes emerged: (1) PREMs for people with palliative care needs ought to be tailored to the needs of this population; (2) PREMs should appraise whether the needs of families have been met in addition to those of patients; (3) PREMs for inpatients with palliative care needs ought to be easy to use, brief and incorporate space for free text alongside each question; (4) Implementation of PREMs for people with palliative care needs ought to consider who administers these, when and how often; (5) PREM data need to be specific enough to inform process change and/or care provision; (6) Patients and families require meaningful feedback to encourage PREM completion.

**Conclusions:**

This study provides practical guidance for PREM selection and implementation to inform improvements to care for inpatients with palliative care needs.

**What is already known about the topic?**

- Globally there are high numbers of patients with palliative care needs requiring care in the hospital setting and this number is expected to rise;
- Enabling high quality, accessible and integrated care for inpatients with palliative care needs, and their families, is an important healthcare priority;
- Patient Reported Experience Measures (PREMs) are gaining international attention as a measure of healthcare quality, but how to capture these data in terms of tool selection and mode of administration, within the palliative care population is unclear.

**What this paper adds?**

- Patients with palliative care needs and their families prefer a PREM tailored to their needs and that is easy to use, brief and offers the option for free text responses.
- No consensus was identified regarding who should administer PREMs for this population, when and how often, nor regarding the ideal mode of administration.
- PREM data can be used for both improvement work and as part of care delivery with these distinct purposes having implications for how and when PREM data are collected.

**Implications for practice, theory or policy?**

- Implications for practice centre around two key areas. Firstly, that hospitals adopt a brief PREM (<12 item) that either: provides feedback on a small number of domains of importance for quality palliative care, as defined by palliative inpatients; and/or provides a deeper dive into one domain of importance for quality inpatient palliative care (e.g. effective communication). Secondly, that hospitals clearly define how they plan to use the adopted PREM prior to its implementation and agree who and how it will be administered, and how the data will be fed back to participants and clinicians to enable change.
- Implications for research centre on how to collect and use PREM data in a meaningful way to enable improvements, within resource constrained environments.

## Introduction

Enabling high quality, accessible and integrated care for inpatients with palliative care needs and their families is an important healthcare priority.^1,2^ Appraising quality of care from the perspectives of inpatients and their families is one mechanism to inform improvement efforts.^3–5^

Patient Reported Experience Measures (PREMs) are *‘. . .survey tools used to record patient perceptions about various elements of the healthcare they received*’ (p. 1).^6^ Although PREMs are gaining international attention as a measure of healthcare quality^3,6^ for palliative care service appraisal,^4^ which PREMs are optimal and how best to implement these remains unclear.^7^ A recent systematic review identified 44 PREMs designed for people with advanced serious illness and/or their families.^7^ The variable alignment of these PREMs with noted areas of importance for safe and high-quality inpatient palliative care is described in addition to the fact the majority of items within listed PREMs are written in language that limits accessibility.^7^ In addition, there are a large number of PREMs developed for other care contexts^3^ which may or may not be applicable in informing improvements in care quality for patients with palliative care needs. A recent scoping review identified methods used globally to capture patient experience data within the hospital setting.^8^ This review identified 30 publications focused on capturing patient experience data and noted the majority used formal, paper-based surveys.^8^ The need to ensure PREM data triggers meaningful reflection and change is important, ideally by means of qualitative as well as quantitative data to provide greater depth.^9^

In Australia, hospitals are accredited following assessment against the National Safety and Quality Health Service Standards (‘Standards’).^10^ The standards include several items of relevance for high quality palliative care.^10^ The Australian Commission on Safety and Quality in Health Care, which oversees the standards, developed and validated a generic PREM for inpatients (the Australian Hospital Patient Experience Question set) that has demonstrated acceptability and feasibility across 36 hospitals.^11^ However, it is unclear whether this PREM is optimal for patients with advanced serious illness. Therefore, this study aimed to explore the perspectives of inpatients with palliative care needs, their family members, and the clinical team regarding the use of a generic PREM as compared with a PREM designed for people with palliative care needs and related implementation factors.

## Method

### Design

A qualitative study was undertaken using semi-structured interviews and focus groups and integrated thematic analysis. We drew on the tenets of pragmatism to enable a focus on a defined research problem, varied viewpoints and to enable a practical, outcome-oriented process.^12–14^

### Setting and participants

Eligible participants were recruited from three wards in two departments (cancer care and internal medicine) from an Australian tertiary metropolitan hospital. There were five groups of participants: inpatients with palliative care needs; family members of these inpatients; clinical representatives (medical, nursing and allied health); hospital executive and quality management personnel; and consumer representatives.

### Eligibility criteria

*Semi-structured interviews:* Inpatients and family members were eligible if they were: an adult (aged ⩾18) with palliative care needs within one of the participating hospital wards, or their adult family member; and able to provide informed consent. Patients with palliative care needs were defined as having ⩾2 general indicators of deteriorating health and ⩾1 clinical indicator of a life-limiting condition^15^ in accordance with the Supportive and Palliative Care Indicators Tool (SPICT™).^16^ The SPICT tool was designed to assist in identifying people with deteriorating health due to advanced serious illness.^16^ Patients noted by the clinical team to have cognitive impairment limiting ability for survey completion were not eligible to participate, however their families were.

*Focus groups:* Key stakeholders were eligible if they were: (1) clinicians (nurses, doctors or allied health) from participating wards; (2) hospital executives or quality management personnel from participating departments; or (3) consumer representatives recruited via the hospital’s consumer engagement committee with self-nominated experience with palliative care.

### Research team

A nurse researcher with palliative care expertise (CV) supported by experienced researchers with oncology, palliative care and qualitative research expertise (JLP, PY, EB and TL) oversaw all interviews and focus groups. A reflexive journal captured reflections immediately after each interview or focus group. Field notes informed team discussions when uncertainties arose, to support rigour.^17,18^

### Recruitment

*Inpatients and families:* The recruitment target was 24 participants (12 patients and 12 family members) based on guidance that a sample size of 9–17 provides sufficient perspectives to enable identification of recurring themes when taken from a relatively homogenous study population informed by a defined research objective for carefully selected participants samples.^19,20^ Purposive sampling was used guided by the eligibility criteria. The research nurse screened and approached inpatients being sensitive to their condition. If an inpatient was ineligible due to cognition and a family member was at their bed-side, the family member was invited to participate. Participants were provided with adequate study detail to provide informed consent.

*Key stakeholders:* An initial email (and two follow-up reminders) with key information about the study was sent to eligible stakeholders (clinicians, clinician executives and quality management personnel from participating wards) inviting participation. This same process occurred via the hospital consumer engagement committee to invite consumer representative participation. Potential participants were provided with adequate information and written consent obtained. The study particularly wanted to understand implementation factors, in addition to feedback about the selected PREMs, to inform recommendations. This is why organisational consumer representatives were invited to participate, in addition to patients and family members currently receiving care.

### Data collection

Interviews were conducted in September–November 2021, audio-recorded, transcribed verbatim and field notes taken. The interview guide focused on understanding perspectives about two specific PREMs^21,22^ (Textbox 1) chosen to provide examples for participants to reflect on. These were purposely chosen to include one designed for all inpatients and the other designed for people living with advanced serious illness. The chosen PREMs were:

1. The Australian Hospital Patient Experience Question Set (AHPEQS)^22^ – this validated PREM has been designed for all inpatients and is not specific for people with advanced serious illness; and
2. The ConsideRATE tool^21^ – this validated, brief PREM has been designed for inpatients with advanced serious illness.

Demographic data were also collected to describe the study sample, including: age, gender, nationality, culture and highest level of education.

**Textbox 1.** Semi-structured interview question route.

1. How would you react if someone asked you to complete this document? 2. Do you find this survey easy to fill out? 3. Is anything confusing or poorly worded within the survey? 4. This survey was not designed specifically for people living with a serious illness. Do you think this matters? Are there any important questions missing from your perspective? 5. We added the final free text question ourselves – what do you think of this? 6. We have found another brief survey (ConsideRATE tool) that has been developed for people with serious illness in particular – could I ask you to have a look at this and tell me which survey you prefer and why? 7. Is anything confusing or unclear about the layout? a. Would you suggest any changes? 8. Can you tell me what you think of the length/number of items? 9. Can you walk me through how you would complete this (prompt re mode – paper/personal device/other/want assistance or independent)? 10. Right now, these questions are only for patients. What do you think about this in relation to family members? Should we create a set that is similar for them to use? 11. Do you think we should give this survey out to inpatients? If so, how often would you like to see a survey like this? 12. Any other comments or questions about this survey?

Focus groups occurred in October 2021 and were audio-recorded, transcribed verbatim and field notes taken. The consumer representative focus group focused on the interview guide used with inpatients and families (Textbox 1). Clinicians, executives and quality improvement participants focused on understanding perspectives about the two included PREMs,^21,22^ how they might be implemented, inform change and which tool participants would prefer to use and why (Textbox 2). Given time constraints for clinicians in busy clinical settings, focus groups were offered to enable multiple perspectives in a defined time period. The study design enabled patients and carers to contribute via individual interviews to account for variances in health condition, privacy when recounting personal experiences and to cater for those who may have felt less able to speak up in a group setting. Demographic data were collected to describe the study sample, including: age, gender, professional role and highest level of education.

**Textbox 2.** Focus group interview question route.

1. What are your views about the Australian Hospital Patient Experience Question Set? 2. Do you think this tool would provide useful data for you to work with? 3. Are there any parts of this tool that concern you or that you would like changed? 4. Can you tell me what you think of the length/number of items? 5. How do you think this tool could be administered within the clinical environment (prompt re mode – paper/personal device/other/want assistance or independent + prompt re timing – regular Vs snapshot? 6. Right now, these questions are only for patients. What do you think of that? Should we make a separate version for family members? 7. This survey was not designed specifically for people living with a serious illness. Do you think this matters? Are there any important questions missing from your perspective? 8. We added the final free text question ourselves – what do you think of this? 9. We have found another brief survey (ConsideRATE tool) that has been developed for people with serious illness in particular – could I ask you to have a look at this and tell me which survey you prefer and why? 10. If you had the choice to implement just one of these tools onto the ward – which one would you prefer to use and why? 11. Any other comments or questions?

### Data analysis

Integrated thematic analysis^23^ occurred via data immersion, coding, categorising and generation of themes. Transcripts were checked against audio-files at the completion of each interview, before being entered into NVivo 12 (QSR International) for management. Line by line coding was completed to generate codes before further analysis to enable categories and theme generation. Coding was informed by the semi-structured interview guide and two identified PREM examples. Inpatient and family voices were prioritised throughout analysis. Coding and theme development was completed by one reviewer (CV) with review by all members of the research team to provide feedback, further develop analytical themes and resolve areas requiring consensus.

### Ethics

Ethical approval was granted in July 2021 with reference number: HREC/2021/QRBW/77494. The Standards for Reporting Qualitative Research (SRQR)^24^ informed reporting of results with 21/21 standards addressed.

## Results

### Patient and family members

Seventy-six participants were approached across all wards, and of those, 49 declined and 27 participants (19 inpatients and 8 family members) proceeded to take part in a semi-structured interview (40% response rate). The inpatient and family participants included 14 females and 13 males, with a mean age of 66.4 (10.8 SD) years. Most participants were inpatients (*n* = 19; 70%), born in Australia (*n* = 18; 67%) and with a malignant diagnosis (*n* = 18; 67%) (Table 1). Twenty-six face-to-face interviews and one family telephone interview were conducted with a mean duration of 22 min (range 12–34 min) per interview. No interviewees required a translator to enable participation.

**Table 1.** Demographic characteristic of participating patients and family members (*N* = 27).

	*N* (%)
Gender	
Female	14 (52)
Male	13 (48)
Country of birth	
Australia	18 (67)
New Zealand (NZ)	3 (11)
United Kingdom (UK)	3 (11)
India	1 (4)
South Africa	1 (4)
China	1 (4)
Participant	
Patient	19 (70)
Family member	8 (30)
Diagnosis	
Malignant	18 (67)
Non-malignant (renal failure, dementia, heart failure or chronic obstructive pulmonary disease)	9 (33)
Level of education	
Did not complete secondary education	12 (44)
Completed secondary education	3 (11)
Advanced diploma/diploma	3 (11)
Bachelor degree	2 (7)
Postgraduate degree	7 (26)

### Health professionals (clinicians, health executives and quality improvement personnel)

Two focus groups composed of nine health professionals were run in October 2021 and one executive participated in a semi-structured interview due to availability. Health professionals were mostly female (*n* = 6, 60%) with high levels of postgraduate education (*n* = 7, 70%) (Table 2).

**Table 2.** Demographic characteristic of participating health professionals (*N* = 10) (clinicians (*n* = 5) and executives (*n* = 5) ).

	*N* (%)
Gender	
Female	6 (60)
Male	4 (40)
Age	
25–40 years	4 (40)
41–60 years	5 (50)
>60 years	1 (10)
Professional role	
Nurse, clinical nurse, nurse consultant, nurse educator	4 (40)
Executive nursing – nursing unit manager, assistant nursing director	3 (30)
Medical – fellow and specialist	3 (30)
Highest level of education	
Bachelor degree	3 (30)
Graduate diploma/graduate certificate	4 (40)
Masters	3 (30)

Consumer representatives (*n* = 5) were mostly female and born in Australia, over 60 years of age and highly educated (Table 3).

**Table 3.** Demographic characteristic of participating consumer representatives (*N* = 5).

	*N* (%)
Gender	
Female	3 (60)
Male	2 (40)
Age	
60–65 years	3 (60)
70–80 years	2 (40)
Country of birth	
Australia	4 (80)
England	1 (20)
Highest level of education	
Bachelor degree	2 (40)
Graduate diploma/graduate certificate	1 (20)
Masters	2 (40)

Illustrative participant quotes are embedded into the analysis to enhance transparency and trustworthiness of data presentation.^25^ A broader representation of illustrative quotes is available in Supplemental Appendices 1 and 2.

Six themes emerged from the analysis: (1) PREMs for people with palliative care needs ought to be tailored to the needs of this population; (2) PREMs should appraise whether the needs of families have been met in addition to those of patients; (3) PREMs for inpatients with palliative care needs ought to be easy to use, brief and incorporate space for free text alongside each question; (4) Implementation of PREMs for people with palliative care needs ought to consider who administers these, when and how often; (5) PREM data need to be specific enough to inform process change and/or care provision; (6) Patients and families require meaningful feedback to encourage PREM completion. Details about each theme are summarised below:

#### PREMs for people with palliative care needs ought to be tailored to the needs of this population

Inpatients desired a PREM survey that is attuned to their palliative care needs and not a generic inpatient measure that all inpatients are asked to complete:

*It’s telling me about a point in time when I’m in hospital. And it probably doesn’t cover in regards to the progress with my chronic condition . . . I find with my chronic condition is that I’m doing these surveys but I’m not getting answers in regards to, and obviously there’s a reason for that. But every individual case is completely different. But I’m not getting answers of what I’m expecting next and that sort of can be frustrating yeah. Patient 19 (54yr male with malignant illness)*

Similarly, clinicians preferred a PREM designed for inpatients with palliative care needs as it provides information that is more useful for them to work with:

*I guess the specific information you get from here you can use. Whereas I don’t know like if question four, I felt cared for, like if they circle . . .I guess you’d have to, you’d need them to elaborate more and be like ‘Okay well why did you not feel, why did you say never or why did you say rarely’? Whereas this [ConsideRATE] is like quite specific to, it’s not as vague as this I guess I would say. (Clinician focus group)*

When provided with choice about two PREMs (ConsideRATE designed for patients with serious illness and the AHPEQS designed for all inpatients), inpatients noted ConsideRATE to be more aligned with their needs:

*It’s more in tune with me. . . . . .it reflects that they know what page I’m on love.. they know my needs, they all know me. Patient 15 (78yr female with non-malignant illness)*

Although some questions are direct within the tool designed for people with palliative care needs, inpatients noted this was important as it addressed issues that might otherwise be overlooked:

*Only because the most important issue with people in my and a lot of other people’s situations here is that you have a finite time to live and so at some stage you’re going to be needing to either discuss or come to grips with that. Which I think Question 7 is a very important one. Patient 24 (64yr male with malignant illness)*

Clinicians also described that a PREM specifically designed for people with serious illness provides more directed feedback for their use:

*There’s more specific sort of examples of things that the patient is almost prompted with to, rather than maybe from this one* [AHPEQS]*. . . where it feels a little bit more about like a broad general feeling of overall care and you know rather than pinpointing exactly what went really well, wrong. (Clinician focus group)*

#### PREMs should appraise whether the needs of families have been met in addition to those of patients

The importance of measuring the experience of care quality from a family member’s perspective in relation to their own care needs, was noted:

*I think that would be helpful because family members are often advocating for someone who is very sick and yeah I think it would be helpful. A similar set of questions but from the point of view the family member’s point of view. Family member 6 (56yr male family member for non-malignant illness)*

The need to understand care quality in relation to family members was also emphasised by patients:

*Well I think there should be one for family members and one for patients because family members have different needs. . .I’m doing everything I can they need support as well. Patient 10 (74yr female with malignant illness)*

As ConsideRATE can be completed by family members there was some discomfort about the use of a proxy rating with respondents noting this PREM would be difficult to complete without including the patient:

*Whose perspective? Because you said that they’re completing it on behalf of their loved one. So it’s not them completing it, who is this for? It’s not for the caregiver, it says people who are ill or for their caregiver. Not it’s for this person who is ill but the caregiver is actually completing the form. So that’s very confusing. Patient 12 (57yr female with malignant illness)*

Clinicians and hospital executives identified their concern about a proxy rating due to differing experiences of care provision between inpatients and families, noting the family rating of patient need would need to be recorded within the data collection and reporting processes:

*Only if you identify that it was the family member that filled it in and not the patient. Because the patient’s expectations may be very different from the family member’s expectations. (Executive Focus group)*

#### PREMs for inpatients with palliative care needs ought to be easy to use, brief and incorporate space for free text alongside each question

Formatting of the PREM is critical for this patient population as poor formatting leads to survey disengagement and contributes to cognitive load:

*I just find the formatting because of so close I do find it hard to sort of concentrate on that line and do the answer. Patient 19 (54yr male with malignant illness)*

Inclusion of the option for a free text response to each question was helpful both in terms of enabling tailored feedback but also ensuring white space within the PREM itself:

*If I felt strongly enough to write a comment I would want to do it in a way that it was clear cut and there’s no way anyone could misinterpret which question was linked to which answer. Family member 18 (69yr female family member for malignant illness)*

In addition to aspects of formatting, accessible language is also critical. Patients noted their need for language that is not too clinical:

*I’d say if I was a patient with a tertiary education I’d say no but given that I’ve mixed with a lot of very ordinary people I’d say some of the language is too clinical. Patient 20 (80yr male with malignant illness)*

Consumer representatives agreed with the need for accessible language and also noted the importance of tailoring questions so they do not bias a particular response:

*The words of some of those could be improved but I think the way it’s framed it suggests an openness or honesty in the response whereas this one* [AHPEQS] *even though I like the format of it better I think it’s more readable it does sort of pre-empt. (Participant 1, 64yr female) . . . You’re right because even in Question 1 it says ‘my views and concerns were listed to’ so that’s sort of putting words in their mouth to agree. (Participant 3, 70yr female) . . .It gives you a positive statement. (Participant 4, 80yr male). . . My mother never wanted to be a problem to anyone. She would have agreed. (Participant 1, 64yr female)*

The need for PREMs to be accessible and easy to use was emphasised given many patients with palliative care needs experience cognitive impairment as they are living with advanced disease, have acute concerns and often require strong medications:

*Yeah but I mean to say the way I write things and the way my brain goes around the corner it could be quite twisted but there again it couldn’t be it just depends on what I say and it’s what I do you know. Just what I said just before you know I probably went from down there up to here and then shot out there somewhere and come back again. It is what it is. Patient 26 (66yr male with malignant illness)*

Participants noted the helpfulness of having small prompts added to each question as it focuses the mind on what is truly being asked:

*It kind of has that just that single small explanation of what the question is getting at is really useful I think. Family member 6 (56yr male family member for non-malignant illness)*

Brevity for the survey was appreciated, however patients also noted the PREM should not be so brief that the data collection is not meaningful:

*Can’t do it too short then you’re not going to get the depth of knowledge that you need. And I think most of us should be able to cope with twelve questions. Patient 12 (57yr female with malignant illness)*

Preferences in relation to the measurement scale were noted. Some comments were made about the difficulty of rating a feeling as very good or very bad:

*. . . because I guess you’re just trying to associate a feeling with the care then rather than umm you know like a net promoter score, sort of rating. . . .it’s very hard to say something is very good or very bad I would say. So I don’t know if you would get a good range of responses with that category. Whereas in the other one having always, sometimes, mostly probably covers the same sort of umm response that you’re wanting to get from them I guess. Family member 5 (43yr female family member for non-malignant illness)*

In addition to this, the use of ‘very bad’ was questioned as participants noted it would need to be extremely bad for someone to use this:

*I don’t think a hospital anywhere would have very bad or even bad. One of those two just bad would do. . .It would have to be an extreme for me. Family member 18 (69yr female family member for malignant illness)*

The presentation of the scale itself adds to ease of use with care needed in relation to positioning of response options:

*Okay my first thing would be when I think of something as being very good that’s my number ten or five whatever it is. . .and that should be at the end of my row yeah? That should be my last thing because it’s the highest. And then I get doesn’t apply so that confuses me because I naturally go to tick this box and actually I need to tick this box. Patient 12 (57yr female with malignant illness)*

The need for a neutral response was also noted by some:

*I would like in between good and bad like average. . . Because I don’t like to jump from good to bad you know it doesn’t look good you know. Family member 17 (54yr female family member for non-malignant illness)*

#### Implementation of PREMs for people with palliative care needs ought to consider who administers these, when and how often

While participants expressed a willingness to provide feedback to inform appraisal of care quality to inform improvement, they stated the timing for this is important as completing a PREM when acutely unwell would not be welcomed:

*Depends on what condition I am in. Today I’m in a very good condition. When I came in on Thursday I was in a very, very bad state. So if someone was to come to me on that day I would have probably told them go away. Patient 1 (49yr male with malignant illness)*

Participants said the PREM should be captured at multiple time points to capture the full experience.

*Because it needs to be taken not just once I guess you know so that there’s different parts of when you are an in-patient. There’s when you’re admitted, so when you’re admitted the answers might be different to when you’re moved to a ward and being treated to when you’re discharged. So if it was to be done to an in-patient the timing of the inpatient’s road map would need to be considered in that as well. Family member 5 (43yr female family member for non-malignant illness)*

The importance of preparing patients to understand the intent and purpose of PREM completion was noted within the clinician and consumer representative focus groups. The sensitivity of the content within these tools was discussed as was enabling confidence for patients to feel safe in answering honestly without impacting their clinical care:

*I actually think it’s important to give direct feedback but I agree you’re not going to get that from patients unless they feel confident that they can be authentic when they answer this. I think that’s the big barrier. . . But I think patients need help to understand that it’s okay to be honest and they need help and support to be guided that they’re not going to be judged by what they’re saying and it’s not going to come back at them. It won’t affect their treatment but I don’t know that they feel that. (Consumer representative group)*

Several participants noted that it felt hard to answer PREMs. Participants also stated it would be difficult to distinguish factors relating to care provision rather than individual pain and illness factors and noted some questions were very broad:

*There’s a lot of motherhood sort of statements you know that the sort of all encompassing. . .No my individual needs were met? Well some of them might have been some of them maybe not I don’t know I mean I’d have to think about what you mean by individual needs were met. Patient 13 (70yr male with malignant illness)*

Most patients stated a preference for completion of a PREM in paper form but a variance was noted with some electing for completion via a text or email:

*It’s not so easy on a device especially if it’s a phone because they’re smaller and I have to tell you I never take surveys on the computer. I certainly wouldn’t on my phone. Patient 20 (80yr male with malignant illness)*

Variance was also noted in relation to whether a patient was willing to complete a PREM independently or with assistance, relating to how unwell a person was on the day the survey was administered:

*I think if someone had a serious illness they’d find it hard to engage with a survey period. I can’t think of some of the patients in Mum’s bay I don’t think any of them would be able to engage with this because they’re so sick. Family member 6 (56yr male family member for non-malignant illness)*

#### PREM data need to be specific enough to inform process change and/or care provision

Clinicians stated that PREM data need to provide enough detail to enable an understanding of exactly what areas need improvement:

*If it was a little bit more specific on you know were your needs addressed or whereabouts you know could we improve, you know directed a little bit more if that makes sense? (Clinician focus group)*

They noted using a PREM for higher level screening could be useful, but a deeper understanding would then be required to inform improvement work:

*But then I think there would have to be a second screening, a point before we got to trying to implement some sort of project to know what we’re trying to target. So yes so even if this was just like a first step and then depending on specific areas that maybe have more problems than others or whatever doing a further drill down into what those issues actually look like to the patient. (Clinician focus group)*

The complexity of collecting these data for broader quality improvement and not for immediate clinical response was noted. ConsideRATE was felt to provide important information to tailor discussions and care planning in real time:

*I think if you had access to this* [ConsideRATE] *at the time it could bring up some important conversations (Clinician focus group)*

Clinicians noted their concern in collecting this information and not addressing any noted areas of concern:

*I do worry when it’s collected if you don’t respond to it as well. . . why would you ask them if you’re not going to do anything about them sort of thing. (Executive and quality improvement focus group)*

Patients and families agreed, noting that PREMs could be useful to enable an immediate clinical response:

*You know umm with myself like wanting to speak to someone about palliative care. And having that as an option in the questionnaire. Then they can come to you. . .I’m sort of a little bit sort of shy a little bit sort of not up front in actually ringing up a person and saying ‘I want to speak to someone’ but I prefer to be approached in regards to that sort of thing. It would be nice to have that as an option in a way. . . I’d like to get, instead of writing that information out, getting more details. And more generalised like I said you know would you want any more assistance from palliative care, from dieticians etc. Just as a tick box than writing it down because as I said you don’t know what’s available through Queensland Health and all that sort of thing. I didn’t know a thing about palliative care until recently and I’ve been in the system for four years. Patient 19 (54yr male with malignant illness)*

#### Patients and families require meaningful feedback to encourage PREM completion

Patients and families noted the importance of being provided with meaningful feedback in response to PREM completion. This feedback needed to be specific rather than a generalised ‘thank you for time provided’. It was noted that time is particularly precious for people with palliative care needs, and honouring this time through meaningful feedback is required:

*I would like to think if I’ve devoted half an hour or an hour to filling out a survey or whatever that I would get some sort of return feedback to say well this is what resulted from participation in this survey. This is the actions that we’re taking or this is what people didn’t agree with or whatever you know . . . You know and with all this sort of end of life stuff you’re talking about people that really haven’t got a lot of time to spare. So we don’t want to be spending hours and hours bogged down on paperwork. But I mean I would agree with trying to help somebody in the future if we can improve the services. But I’m not going to fill out a survey if it’s just going to be thrown in a too hard basket somewhere. Patient 13 (70yr male with malignant illness)*

## Discussion

Participants in our study expressed a preference for a PREM tailored to the needs of people living with advanced disease that is easy to use, brief and offers the option for free text responses, compared with a generic person-centred PREM. Participants views varied on who should administer PREMs, when and how often. Meaningful feedback to patient and/or family member respondents was deemed important, including specific information about how PREM data were being used to inform system improvements. Understanding the specific needs of families in addition to patients was identified as necessary given the unique requirements for people living with palliative care needs.

This study confirms the need for tailored tools that appraise issues of importance for people living with advanced serious illness,^26–28^ given these areas of importance have a profound impact on patient experience. The use of generic PREMs risks losing the focus on what matters most for this population. Conversely the implementation of a structured PREM based on what is important for people with palliative care needs brings complex care needs to the forefront, centring clinical care and improvement work accordingly. There are little data about clinician perspectives and experiences in collecting and using PREM data^29^ to better understand patient and family needs. Clinicians in this study wanted PREM data to be specific enough to improve care provision or drive change at ward level. This distinction is important, as the need to identify patients to inform individual care limits opportunities for anonymous feedback. Unlike patient reported outcome measures (PROMs), PREMs require patients to rate care quality, which can be uncomfortable for patients due to the clinician-patient/family power imbalance.^30^ It is also noteworthy that collecting feedback about how clinical teams and/or units can improve may be limited when using PREMs due to feedback generosity leading to a ceiling effect, with the need for further methods that encourage constructive criticism.^31^

Another important consideration for implementing a PREM for patients with palliative care needs is their varied levels of cognition due to impacts of illness, severity of illness or related medications used for symptom relief.^32^ Patients within this study confirmed the need for a PREM to be easy to use with white space available to enhance visual engagement and with language that was not too clinical. Patients in this study appreciated the inclusion of prompts to centre the respondent’s thinking to ensure they were answering the question asked. This is important as this supports participation for patients who have the ability to self-report but may otherwise be limited in answering longer and more complex PREMs given they are so unwell.

Of the two PREMs used in this study, patients resonated more with the ConsideRATE^21^ PREM stating the survey questions aligned more closely to their current needs and noted areas of importance for quality care. This makes sense given the development of ConsideRATE was informed by a systematic review of domains of importance for inpatients with palliative care needs^27^ and further co-design work to refine these domains into a brief, targeted PREM.^21^

## Recommendations for future practice

Recommendations for future practice centre around two key areas. Firstly, that hospitals adopt a brief PREM (<12 item) that either: provides feedback on a small number of domains of importance for quality palliative care; and/or provides a deeper dive into one domain of importance for quality inpatient palliative care. Secondly, that hospitals clearly define how they plan to use the adopted PREM prior to its implementation and agree how it will be administered and how the data will be used to enable change.

## Recommendations for future research

Recommendations for future research centre on how to collect and use PREM data in a meaningful way to enable improvements, within resource constrained environments.

## Strengths and limitations

This study has several strengths including the defined sampling approach, interviewing and data analysis. Sampling across oncology and general medical wards enabled representative sampling of the true population of people with palliative care needs in hospitals. Study limitations relate to asking about perspectives for PREM use within a population of patients with evidence of cognitive impairments. This was evident in transcripts where not all patients displayed a clear understanding of all questions asked. This may have affected the depth of responses for some participants. However, this also provides real information from a cohort of patients who are explicitly the group we are seeking to work with. Therefore, witnessing and working with their cognitive limitations informs analysis and study outcomes and is therefore also a strength of this work. Views of people from culturally and linguistically diverse backgrounds, and Indigenous Australians were underrepresented. In addition, access to family members was limited due to impacts from the COVID-19 pandemic given visitor restrictions. In addition, a single coder completed coding and categorisation and whilst robust group consensus procedures were in place to review and develop these categories into themes, this could have led to analysis bias. Finally, data from patients and family members were accessed via 1:1 interviews whereas clinicians, clinical executives and consumer representatives provided input through focus group formats. This variability in data collection may have created a bias in data received.

## Conclusions

Supporting clinical teams working in busy hospital settings to provide optimal care for people with palliative care needs is urgently required. One mechanism to do this is through provision of regular data describing patient and family perspectives about care quality. This study reviewed two PREMs resulting in practical guidance about key areas that ought to be considered when choosing and implementing a PREM to inform improvements to care for inpatients with palliative care needs.

## Supplemental Material

sj-docx-1-pmj-10.1177_02692163231209845 – Supplemental material for Perspectives of inpatients with palliative care needs, their families, clinicians and key stakeholders on measuring quality of hospital care via patient experience measures: A qualitative study Click here for additional data file. Supplemental material, sj-docx-1-pmj-10.1177_02692163231209845 for Perspectives of inpatients with palliative care needs, their families, clinicians and key stakeholders on measuring quality of hospital care via patient experience measures: A qualitative study by Claudia Virdun, Elise Button, Jane L Phillips, Patsy Yates and Tim Luckett in Palliative Medicine

sj-docx-2-pmj-10.1177_02692163231209845 – Supplemental material for Perspectives of inpatients with palliative care needs, their families, clinicians and key stakeholders on measuring quality of hospital care via patient experience measures: A qualitative study Click here for additional data file. Supplemental material, sj-docx-2-pmj-10.1177_02692163231209845 for Perspectives of inpatients with palliative care needs, their families, clinicians and key stakeholders on measuring quality of hospital care via patient experience measures: A qualitative study by Claudia Virdun, Elise Button, Jane L Phillips, Patsy Yates and Tim Luckett in Palliative Medicine
