# Supplementary material for: Perspectives of inpatients with palliative care needs, their families, clinicians and key stakeholders on measuring quality of hospital care via patient experience measures: A qualitative study
Source: Palliat Med. 2023 Nov 3;37(10):1498–508. doi: 10.1177/02692163231209845 (PMC10657505; doi:10.1177/02692163231209845)
Supplement: sj-docx-1-pmj-10.1177_02692163231209845 – Supplemental material for Perspectives of inpatients with palliative care needs, their families, clinicians and key stakeholders on measuring quality of hospital care via patient experience measures: A qualitative study [file sj-docx-1-pmj-10.1177_02692163231209845.docx]

**Inductive coding – 27 interviews and 3 focus groups**

| **Initial coding (inductive – within NVivo)** | **Related quotes** |
| --- | --- |
| **Ability to use this data to inform change** | *Reference 1 - 1.31% Coverage*  *R1: Yeah so I think in a lot of the feedback I received just in general, not even necessarily just with like end of life care is that consumers seem to notice how fragmented the health care system is. And they’re not privy to a lot of the conversations that are occurring in the background. So when they’re feeling like their needs aren’t being addressed or they’ve got a concern, they’re not sure if the message has made it from the bedside nurse to the treating doctor or the speech pathologist or whoever it might be. And in my ward we don't have the sort of ability to do rounds with Allied Health nursing and medical, still very you know one person after the other and separate. So it’s easier than to, if it was a little bit more specific on you know were your needs addressed or whereabouts you know could we improve, you know directed a little bit more if that makes sense?*  *Reference 2 - 0.34% Coverage*  *if they say my needs are always met, always, they don't need to distinguish because that’s everyone. But in question three where it’s like please elaborate it would be like please elaborate on why you felt, like who was lacking?*  *Reference 3 - 1.60% Coverage*  *R: Depends what sort of information you want. If you want an overall sort of picture from the patient about how they’ve perceived their care versus if you’re looking at it from a quality improvement or you know feedback to the teams on the ward you can probably need more specific information because as you were saying they might greatly dislike their medical care. Or love the nursing staff or they may just be missing care in one specific very specific thing. Like whether it be pain or sleep or whatever it might be and then they’re very, very focussed on that and then that can change everything else. So if this seems like an overall sort of assessment for the patients on their total care. But I think that that’s okay to compare across maybe units or something but if you want to get these (actual actions??) about how they might be able to improve then you probably need more specifics to go back with. Because if you just say people aren’t happy and they’re saying that their care needs aren’t met. And you go well what care needs? And then…so that would be my comment.*  *Reference 4 - 0.71% Coverage*  *R1: I mean maybe yeah. But then I think there would have to be a second screening, a point before we got to trying to implement some sort of project to know what we’re trying to target. So yes so even if this was just like a first step and then depending on specific areas that maybe have more problems than others or whatever doing a further drill down into what those issues actually look like to the patient. To them yeah. Have that inform whatever we do from it. But like…*  *Reference 5 - 1.14% Coverage*  *So overall would you say that you think even just this higher level screening brief tool would provide useful information for you at a ward level?*  *R: I think it would.*  *R1: Yes. I do.*  *R2: It’s very similar to what we already have the patient experience survey. And on oncology ward it’s that survey that feedback is really helpful. Particularly from like a quality and safety and even that’s, it helps us kind of figure out what we need to improve on. So because the questions are quite similar I do, it would be helpful but this obviously focuses more obviously the patient experience is the entire thing. Like food and like hand hygiene all that kind of stuff whereas this is more specific to like their own needs. Which yeah it would be it would help I think yeah.*  *Reference 6 - 2.24% Coverage*  *R3: Ahh probably prefer considerate mainly because I think if you had access to this at the time it could bring up some important conversations about things. Because you know patients are obviously very different in what they want to discuss. Sometimes they’d like to talk about time like to live or they want you know some certainty about that. And then there’s some patients who outright don't want to have to talk about it. And certainly things you know this had got specifics about wills, finances, advanced care directives and all of these things should be addressed while somebody is, particularly if they’re admitted to hospital. And if patients feel like they’re not then I’d actually see this as a really important tool at the time to start a discussion with them. Because it’s a short survey and you can actually see you know it’s one page you can actually see down the list and I don't know how the survey would be given. If it’s given as a confidential thing and then sort of tabulated but it could be useful as a tool at the time to start a discussion. Because if these things aren’t being met while they’re in hospital then you know there’s a couple of things here which are really, really, important I would think for patients. Like advanced care planning and like discussing their illness getting worse and they haven’t felt like they’ve been communicated about those things then I would see that one probably as a better review at a point in time. Particularly before they are leaving hospital.*  *Reference 7 - 1.13% Coverage*  *I: So if I'm hearing it correctly so far we’re thinking commission is good for broad high level information but the considerate tool could be used in clinical care deliveries? So you can give it out and then use that within the MDT to say the patient is worried about this, this and this.*  *R3 I think so.*  *I: Yeah.*  *R: I think that would give a quick snapshot yeah of what they’re thinking.*  *R3: Even as a pre-discharge tool or something like that to you know if they’re going to go home make sure that they’re aware of what’s going to happen and any of their needs are addressed. But that’s kind of, that’s probably why I prefer that one because I think this is good as a yeah as you say as an overall sort of picture of patient’s overall hospital experience.*  *Reference 8 - 0.92% Coverage*  *I: So you think the considerate is the first you’d do and then …*  *R4: ….yeah and so if we were doing bad in any of those things this would be like the right before you were leaving what if we, like did we do a good job. Versus this has very specific things that we could use. And it would be good if you were getting palliative care information as to what areas are we like staff needing extra education to be able to provide adequate needs being met.*  *I: So I'm wondering if you’re sort of saying the commission tool is good for QI sort of work but the considerate tool is good for clinical work?*  *R4: Yeah*  *R: Mmmm yeah.*  *Reference 9 - 1.04% Coverage*  *R1: So I think I agree, I think this was also like a bit more user friendly.*  *I: The considerate tool?*  *R1: Yeah how it is displayed and I guess the specific information you get from here you can use. Whereas I don't know like if question four, I felt cared for, like if they circle …I guess you’d have to, you’d need them to elaborate more and be like “Okay well why did you not feel, why did you say never or why did you say rarely”? Whereas this is like quite specific to, it’s not as vague as this I guess I would say. Which is why I feel like it’s user friendly and it’s quicker and I think that’s what I feel like that’s how you get more numbers. If it’s something that’s quick and ticking a box.*  *Reference 10 - 1.31% Coverage*  *R3: From a clinical point of view this one.*  *I: Considerate? Yep.*  *R3: Yeah but for the reasons I mentioned before I think. You know some patients will not highlight you know their concerns about what’s happening and sometimes they’ll be more willing to do it in a form. But I think as well you have to think about who would help them fill out the form. Because if they’ve got you know medical staff or the people that have been caring for them and then they’re saying you know very bad for attention to their feelings. So maybe a little bit less willing to write that if the person caring for them is sitting in front of them. Particularly if there’s you know if the information is going to be used at a higher level and you know..for those sorts of things. So does it need to be someone independent or do they need to fill it out themselves? I don't know but that might be an issue.*  *Reference 11 - 1.65% Coverage*  *R: I think they have different, I think there’s some different benefits in each of them…*  *R4: ..it depends on what you’re aiming for…*  *R: …yeah in what you’re aiming for.*  *R4: Like in terms of the information that you are wanting to get I think considerate would be useful and you could implement it at whatever stage they are in. This would be easily accessible through an iPad like easier to tick than this kind of one. So it could be used easily through someone with an iPad. And I like the family being able to be involved without skewing too much with the ambiguity of the commission one. So the considerate would give you specific and user friendly things and I think it doesn’t like it’s pretty objective data versus subjective which you’re going to be getting from the commission based. I like this one.*  *I: You like considerate okay.*  *R: I think it’s got a few more palliative driven sort of questions.*  *I: Very much so.*  *R: Which from a palliative care point of view we would find of value yeah.*  *R4: This one would be good for every other patient that I have. That one would be better for my palliatives.*  *Reference 12 - 2.24% Coverage*  *: So you just mentioned question one to five of considerate you could more or less give out to anyone. So if you were to think of those first five questions as opposed to the twelve questions on the commission tool, as a bit of a screener for quality improvement would you have a preference?*  *R2: Definitely considerate.*  *I: Okay the first five?*  *R1: I think that I actually don't find now looking at both of them I don't find the commission one as helpful to really …I still feel the same. That if it was my umm choice of like if it was what we were using to screen people we’d still have to dig down further. Like it really doesn’t give me any tangible information to go with without having to pull the answers apart a little bit deeper.*  *R: It leaves us with more questions doesn’t it?*  *R1: Yep whereas this one I can the considerate like questions one to five for instance, you know it’s, they’re similarly framed about like how respected they felt and our attention to their feelings. But with the you know feeling sad or worried or like a burden might prompt them then to say you know the staff weren’t sensitive to how I was feeling. I did just get some bad news or feeling like a burden I hear a lot about. Just how busy they think the staff are all of the time and so you know like I think I feel like that would prompt them a lot more to…elaborate.*  *R: I think certainly one to five you could hand out, be done very quickly and I think you could do that to a very large population of the hospital.*  *Reference 13 - 0.29% Coverage*  *R4: yeah I think formatting of this one is nicer but the questions on this actually seem to be more in line with what your study is and being able to get information for that with an improvement.*  *Reference 14 - 1.02% Coverage*  *R: Certainly from a palliative care point of view I’d love everyone to think about six and seven.*  *R1: Oh me too.*  *R4: But I think the, like I probably do this it would just be dependent on what patient it would be. Because we’ve got chronic respiratory and then when they’re sick they’re inside is like phhht and they would, these last two questions would make them….*  *R1: …I agree. This is something that should be discussed with everyone but it’s just not necessarily done well. Which is if the first sort of is in a survey then…*  ***1:02:12.5***  *R: …the counselling of it may be harder work than laughs…and that’s a society thing like that’s a hard thing we’re going to change culturally.*  *Reference 1 - 0.68% Coverage*  *R3: We don’t in general med have those strong conversations about the last year of life.*  *R1: Despite all the research that’s right.*  *R3: And you know advanced directors of care you know that should happen in the community before patients come in and if not it should happen whilst they are in hospital. But we still struggle to get that so some of it might trigger.*  *R1: But maybe it just would be another it would highlight ongoing work in that area so it adds to that body of evidence.*  *Reference 2 - 0.85% Coverage*  *R1: Well I think we don’t talk about it enough do we? And I mean I’m clearly biased I’m at one end of the whole spectrum. But as [name] says despite all of the work that’s gone on we still have very low rates of advance health planning or advanced care planning so it doesn’t help.*  *R3: And even though those questions are difficult for some of the young ones to ask like you know you might bring up we have issues all of the time with our complex patients about finances and that. The social worker then sort of deals with those. So do you see it as a tool just for the nurses or for all of the team.*  *Reference 3 - 0.85% Coverage*  *R3: I do worry when it’s collected if you don’t respond to it as well.*  *R1: I mean it’s clearly going to be an education tool isn’t it? It has to be an assess but it’s got to its highlighting global issues. It’s going to highlight higher issues isn’t it and management.*  *I: That’s an interesting point you’re making like [name] you feel if it was administered it would need to be clinically responded to because the questions are so personal. Whereas you don’t feel that so much from the Commission Tool?*  *R3: No probably why would you ask them if you’re not going to do anything about them sort of thing.* |
| **Complexity about shared decision making** | *Reference 1 - 7.00% Coverage*  *R: Yeah like I'm if I come into this situation I'm here for treatment and people have done a lot of research and know a lot more than I do to start with. So I don't want to be telling them what they should be asking me. I don't want to direct them there of sorts.*  *I: So you don't, with this question number five here I was involved as much as I wanted in making decisions about my treatment and care.*  *R: Yeah mostly likely does cover that yes. It’s just….yeah I just must have been at a (??)…*  *I: ..so do you..*  *R: It does give you, you know it gives…*  *I: …so do you feel as though that question is asking whether you were dictating your care to the providers or….?*  *R: Yeah. Yeah that would be about it yeah.*  *I: Okay. How would you reword that question? Do you think you would reword that question at all to make it a little bit more clearer in your eyes?*  *R: pause*  *I: Because I suppose that question might be looking at you being a participant of your care and not a recipient of it.*  *R: Yeah*  *I: So…*  *R: Yeah that’s what I'm sort of getting at I think yeah.* |
| **Complexity for carers to rate as proxy** | *Reference 1 - 1.11% Coverage*  *You see as I read this it’s just more or less it’s to me that looks like it’s for [patients name].*  *I: Yeah so the commission…*  *R: …like I received pain relief that doesn’t apply to me. I can answer that what I think has happened you know I can’t answer that for [patients name] can I?*  *Reference 2 - 1.95% Coverage*  *R: Oh it doesn’t worry me doing it. But I would make sure that Sandra was there with me doing it. Like I would not do just off on my own.*  *I: Okay.*  *R: I’d have to have [patients name] there because [patients name] has got to answer the questions…*  *R1: ..answering…*  *R: ….I’ll write them down but [patients name] has to enter them in her words not mine.*  *I: yeah, no…*  *R: You know as a carer I can only go by what I’ve seen while I’ve been here. And I'm here every…*  *R1: ..does it have a…*  *R: …day.*  *Reference 3 - 0.71% Coverage*  *R1: …does it have a section at the bottom where it says sign, filled out…you know how some forms have umm filled out on behalf of such and such. And then the second person signs it.*  *Reference 4 - 0.29% Coverage*  *If they had a section on …maybe at the top where it said carer/patient …*  *Reference 1 - 2.30% Coverage*  *: Yeah that would not be a bad idea because at least then the patient can do this. [name of patient] wouldn’t be able to do this at the moment his brain’s too busy but for the two together that would be a good idea.*  *Reference 1 - 0.50% Coverage*  *R: No well the fact that I’m here and I know what he’s thinking and his needs and whether they’re met etc. no I could fill it out.*  *Reference 2 - 2.51% Coverage*  *R: No not really I think it’s probably more something that you’re here 24/7, I’m here a few hours a day. It’s more something that we could sit down and fill out together because he obviously knows a lot more about what’s going on in my absence.*  *R2: Any questions that come to the carer about my care are very different for what I say I’ve been sitting here 24/7 and getting the care.*  *R: He knows more about what’s going on than I do but by the same token I’ve got a pretty bloody good idea.*  *R2: If there was something I thought I could complain about.*  *R: Well the first thing you’d do would be to complain to me about it. You know what do you reckon.*  *Reference 1 - 0.92% Coverage*  *R: It’s you know it’s hard to capture I think the nuance of a patient experiencing some of those questions and for one like Mum its difficult because she has dementia.*  *Reference 2 - 2.63% Coverage*  *R2: So from your Mum’s point of view well I’m projecting her experience I guess is that she came to hospital because she had a fall at home, wasn’t injured and then when she was in the hospital setting and I understand this happens, it’s quite a common thing to happen. She fell in the hospital setting and she broke her hip and her shoulder. So if she was looking at this survey she would be thinking well I’m in a worse position now than when I first came in the hospital.*  *Reference 1 - 0.47% Coverage*  *R: This one I don’t understand. I was involved as much as I wanted making decisions about my treatment and care.*  *Reference 2 - 0.33% Coverage*  *R: Well if these are about the patient what sense would it be me saying anything?*  *Reference 3 - 1.42% Coverage*  *R: My view and concern was listened always, mostly, never, rarely, didn’t apply. If he thinks he can get away with it. Like I say are you in pain? Oh no it’s not too bad I’m right. He’s I suppose you’ve got to look at these some of these blokes they’re back from the 60’s and they’re a different type of man to the man you’ve got today you know.*  *Reference 4 - 0.32% Coverage*  *R: Well I think if I filled it out and he then read it I think he would agree.*  *Reference 5 - 1.10% Coverage*  *R: Mostly that top one. No that one I don’t agree with. See the thing is this I can’t understand well when you say when I was in the hospital treatment and care I experienced …*  *I: So with regards to …*  *R: See because he’s in the hospital he doesn’t tell you anything.*  *Reference 6 - 0.54% Coverage*  *R: I’ve received pain relief that met my needs. With him you’d never know. So if it’s in the hospital he’s not going to tell you.*  *Reference 7 - 1.83% Coverage*  *I: So you would find as a carer some of these questions would be very difficult for you to complete?*  *R: To answer yes completely.*  *I: But out of the questions do you feel any of the wording is you don’t like the wording or you don’t understand some of it?*  *R: I experienced … I couldn’t really see that happening in a hospital could you?*  *I: I experienced unexpected harm or distress as a result of my treatment of care?*  *R: I mean I know it does happen.*  *Reference 8 - 0.91% Coverage*  *R: Yeah I would. I mean I know he feels like he’s a burden at the moment but then again see we’re looking I’m looking at it through my eyes I’m not looking it through his eyes and that makes the difficult part of it you know.* |
| **Confronting terminology** | *Reference 1 - 10.88% Coverage*  *I: It has been pointed out that the wording like burden, feeling like a burden or time left to live is quite confronting…*  *R1: …oh…*  *I: …what do you feel about those types, that type of wording?*  *R1: …it’s true though.*  *R: I (???) the question. I remember reading it.*  *I: So it’s just….so feel like a burden is just in the second question.*  *R: Oh yes.*  *I: and then time left to live in the last question.*  *R: How would you rate our attention to your feelings? Things like feeling sad and worried or like a burden and they say…*  *R1: …it’s true…*  *R: …yeah…*  *R1: ..it’s true I often say to mum I worry that …my little things. Like someone setting up my meal tray for instance, it’s not a medical issue. Like I'm not medically but I need someone to help me. And it makes my stay in hospital easy because if I get frustrated I just won’t eat which affects my medical condition.*  ***17:26.3***  *R: And twice this week she’s had a, she’s…they haven’t put a, given her a cup of tea without a top on it and she wore the cup of tea. And then another day she wore the milo. And then she waited for like an hour…*  *R1: …and….and I say to mum…*  *R: ….an hour and a half…*  *R1: ….I worry that…*  *R: ..she’s going to get burnt….*  *R1: ….I’m less of, because this is a medical ward and they deal with medical issues. I'm less…*  *R: …important…*  *R1: …I feel like a burden and I feel like when the nurse come in the other day it was like I could feel her literally rolling her eyes when I asked her to do something. And I do I feel like a burden to my family. But when you come into hospital and you’re hoping that because it’s their job and they’re care professionals, paid care professionals that …that it wouldn’t feel like that. But at times I still do, the fact that they put those words in that survey in my opinion is great. Because then that’s exactly and when you come into hospital and you’ve got doctors at your bed. …and mum has been here. And they’re asking you to sign a ‘do not resuscitate’ order..*  *R: ..they’ve asked about that about three times since she’s been in here.*  *R1: You know.*  *R: Even on dialysis at least once a week.*  *R1: You know you feel like , you feel like you know you know your number is you know, you know you’re not going to live a long life. But being constantly reminded and felt like you’re sort of pushed and they know my response is always the same. But it’s like they have a list and you have to you know….but the fact that they put that into words on a survey on paper to me is like “oh …*  *R: ..finally…*  *R1: …thank God”. Yeah it is harsh and is confronting but it’s people that have …chronic illness they feel that way. Absolutely feel that way. And there’s no sugar coating it or glossing over it because it’s, those words are exactly what I’ve used a thousand times.*  *Reference 1 - 4.39% Coverage*  *I: Some people have pointed out that the wording such as “like a burden” or “time left to live” can be quite confronting. What are your thoughts around that [name of respondent]?*  *R: No I think it’s part of life.*  *I: Okay.*  *R: I think it’s a very important you know a very important conversation to be had or to you know but some people that are doing it or not are at the stage where I am and they’re not ready.*  *Reference 1 - 7.55% Coverage*  *R: I only read the main one sorry things like how would you no because I think at some point no matter what’s wrong with you you don’t like asking for help because you don’t even for I don’t know even for a Panadol or something if you’re a patient. Because you don’t someone’s already run off their feet you don’t want to be a problem sort of thing so you let it go. No “like a burden” no I think that’s realistic.*  *I: What about “time left to live”?*  *R: Where’s that one down here?*  *I: Just down on the last question.*  *R: I think I commented on this the other day in a way that someone that had come in had assured [name of patient] of a painless passing and I had a lot of problem with that because they didn’t know him from [name of someone else (?)] and they’d didn’t know well obviously he knows what he knows. But that person doesn’t know whether he has accepted that or whether it’s be buggered I’m going to give up without a fight. Or my suggestion is even as blunt as that “left to live” I think there should be some care taken to know where the head space is for the patient.*  *I: Yeah.*  *R: And to assure them of a painless passing that’s a little bit …*  *R2: Because I accept it. I’m 80 years old one I know one of these days.*  *R: We’re all going to go.*  ***12:47:1***  *R2: I’m going to die.*  *R: I might not make it home tonight you never know.*  *I: It’s inevitable.*  *R: Still you’re dying the day you’re born.*  *R2: I intend to live for another 20 or 30 years.*  *I: You mentioned that last week.*  *R2: I mean it.*  *R: Don’t get upset. Come on it’s fine. My point being that someone that comes in the door and assumes that he’s accepted or accepting of having cancer without knowing you know half the will half the plus of being sick is the will to fight. You’ve got to determine that so talking about a painless passing if you don’t know the patient, you don’t know anything about them, you don’t know whether they’re accepting of it that doesn’t make them in denial.*  *Reference 2 - 16.05% Coverage*  *I’m not talking for him but he’s going to give it his damnedest to fight it and he probably will win to some degree.*  *I: So that type of wording “time left to live” isn’t offensive to you but it’s important that some form of …*  *R: Those particular words.*  *I: There’s some form of further exploration before you approach someone?*  *R: Exactly just find out just by way of introductory you know I believe you’re not well [patient] and sort of how are you coping? Just judge from his answer.*  *I: Yeah because the two last questions there how would you rate your attention to your affairs and how would you rate your attention to what you can expect those two questions do you think that they potentially might need to be sort of vetoed from the survey until you know where a person’s up to with regards to their journey?*  *R: Well attention to your affairs from my point of view that’s our business.*  *I: Yep.*  ***21:43:3***  *R: Are you alright?*  *I: Are you okay?*  *R2: He’s got cancer I don’t know if he’s worse than mine or not. These two that are seeing him now …*  *R: No they’re probably his family so be careful. He went down for his radiotherapy last week and he got told that they’re going to stop it because his cancer’s grown. I think palliative care for example I’ve already been given an envelope an advanced health directives and powers of attorney and I’ve been asked for it a couple of times. Have you made it out yet? Can we put it on record? No full stop because if he falls over and cracks his head and has a heart attack if there’s an advance health directive in place they won’t save him. The advanced health directive gives them the power to not resuscitate and what if we go home in the car and we have a car accident and he comes in? To me the advanced health directive is a dangerous weapon.*  *I: Okay.*  *R: And he’s going to have to really be in the final stages and then you know the best health directive me. I’m his next of kin and I won’t be more than a few steps away. The Power of Attorney and we’ve been asked if we’ve made out our Wills that’s our business really that’s our business.*  *I: So that question No.6 how would you rate your attention to your affairs you believe that’s more it’s quite personal?*  *R: It’s quite personal and I think that should be by way of idle chat when you walk in. But you see there’s about six different people who have come to him in the last week representing occupational health, palliative care, they’re all basically asking the same questions. They’re all coming from a different direction but they’re asking the same questions.*  *I: So you think it’s really important for them to communicate amongst themselves so you’re not having to?*  *R: Not only amongst themselves but the paperwork our paperwork is our business and long before advanced health directives were the go you know my mother and father didn’t have advanced health directives they each made sure the other one didn’t suffer. And from a Will point of view that’s our business and I’m not rushing to fill them out because as I said you know say he gets bitten by a snake or something and he comes in oh there’s a DNR so they leave him because he’s also got cancer. They get to pick and choose if they only got to not resuscitate him in a cancer related issue but for any other injury or whatever injury or illness it just worries me that it’s a very broad document. Even [name of doctor] the very first day with the three kids there with our three kids there he made out a thing I want you to do that, come in for the appointment, start the chemotherapy and then the last three were advanced health directive, Power of Attorney and I think he verbally said something about make sure your Will’s up to date.*  *R2: He said consider them.*  *R: But yeah that shouldn’t have been and he hasn’t written him off. Even my son said you know I think he was writing him off a bit early you’re talking it might be terminal cancer. But anyone who comes in and talks about our paperwork and the fact that I’m here every day.*  *R2: It’s like in the form of a car it’s going to break down.*  *R: We’ll follow you with a tow truck just in case. Anyway this is all on a tape recorder.*  *Reference 1 - 1.17% Coverage*  *The only one that I sort of double read was the one about unexpected harm or distress because I thought oh gee I hope I never have to worry about that sort of thing.*  *Reference 2 - 10.06% Coverage*  *I: Do you find anything confusing, poorly worded or confronting in the Considerate questions?*  *R: I know you have to have that question in there.*  *I: So you’re pointing to Question 7 there?*  *R: Yes.*  *I: You find that quite confronting?*  *R: I do yes. About getting worse.*  *I: Because it has we’ve done a number of these interviews already and it has a few people have sort of commented on the wording such as “like a burden” and “time left to live”. They have sort of commented on that that it can be quite confronting. Also people have sort of commented about not only Question 7 but Question 6 “how would you rate our attention to your affairs”. This one being well it’s a bit personal so is it your business?*  *R: Though …*  *I: Do you think we should be omitting those questions because it could be personal?*  *R: No because I think it makes people aware. See we had a lady come in and do an ACAT the other day and we already had the Will and the well finances and our Power of Attorney both of us but as far as the advance health directive we haven’t done that. They spoke to us at [name of hospital] about it and they’ve spoken to us about it here. I’ve got the brochure at home that I’ve got to go online and get the form because the other things we went to the solicitor and had but I found that very interesting actually that that was brought up to our attention and we were able to understand a bit more.*  *Reference 1 - 1.50% Coverage*  *R: No. I think if people I mean we’re all going to die regardless of whichever way we want to go around it. But if you’ve got things sorted out and especially if there’s a family that side and a family this side it’s I mean I suppose that little bit different if there’s only one family but if somebody says right this is what I want to do it makes life so much easier.*  *Reference 1 - 1.02% Coverage*  *R: Certainly from a palliative care point of view I’d love everyone to think about six and seven.*  *R1: Oh me too.*  *R4: But I think the, like I probably do this it would just be dependent on what patient it would be. Because we’ve got chronic respiratory and then when they’re sick they’re inside is like phhht and they would, these last two questions would make them….*  *R1: …I agree. This is something that should be discussed with everyone but it’s just not necessarily done well. Which is if the first sort of is in a survey then…*  ***1:02:12.5***  *R: …the counselling of it may be harder work than laughs…and that’s a society thing like that’s a hard thing we’re going to change culturally.*  *Reference 2 - 0.69% Coverage*  *R: I actually think they’re more likely to answer this on here than they are to talk to us about it.*  *R2: I agree.*  *R: Because if we talk to people we explore it. Whereas this just asks this just gives us a bit of information. They’re more likely to just give us an answer they don't have to divulge into it, we don't have to dig into it or anything like that. But it will just give us an answer about well yeah has someone talked to them about the time they have?*  *Reference 3 - 0.65% Coverage*  *R1: And I feel like if the wording is uncomfortable sometimes it’s an uncomfortable time. Like you know I don't know if we should be bubble wrapping that like it is real life. And yeah I really like it because it gives you a question it gives you a prompt. So it removes the test from it, like what do they want me to say? What are the looking for, okay it’s very clear. And then an opportunity to just give an honest easy response. Like…*  *Reference 1 - 0.68% Coverage*  *R5: Question 6 might be for more people that are who can’t fill this form out because if you’re talking Will, finance and advanced directives are they further down the track and able to fill that out?*  *R3: That pre-empting death isn’t.*  *R5: Yeah I’d feel like I’m about to die if I read that.*  *R4: And I think some of the language other than your advanced directives your Will and your finances have absolutely nothing to do with the hospital but in America your finances have a big thing to do with the hospital because they want you to pay.*  *Reference 2 - 1.54% Coverage*  *I: Can I ask you whether Question 7 on the Considerate Tool do you find that confronting where it says, “how would you rate our attention to what you can expect”?*  ***54:59:3***  *R2: It is a bit vague. Some people would need a lot more information.*  *R4: It’s a vague question I don’t fully understand.*  *R1: Well that’s why I don’t like this form a lot of it’s too vague.*  *I: With the things like “illness getting worse” or “time left to live” is that too confronting?*  *R2: Time left to live is a real hard one because so many people get told it and 20 years later they’re still alive.*  *R3: My mother was like that.*  *I: You don’t like it?*  *R1: Not the way it’s worded.*  *I: Because that’s a concern for us as clinicians as to if you were to just give that out would that be upsetting for a patient?*  *R2: Yes because I was burnt 34% of my body and I’m up there in ICU and all my wife wants to know is he going to live or not and it was day four that we finally that she finally got to find that out. He’s going to be alright.*  *R3: Even people with mental incapacity now who are in comas and stuff who historically have been told turn the machines off they can recover you know it’s not necessarily a life sentence anymore.*  *Reference 3 - 1.49% Coverage*  *R1: I think it’s the wording of it. I think it’s the wording the important questions you can see where they’re going I think it’s the wording of it.*  *R3: I think it’s a societal problem people don’t like talking about death and it’s not an easy subject for consumers to talk about. They don’t like to talk about it.*  *R4: I think it’s like it was being said before everybody when you get to a certain age or when you have a serious illness like when I had my breast cancer and was treated for that. You start to think about your morality and you start to think about certain things. But other times in your life when you’re sailing along fine.*  *R3: Society doesn’t like to talk about it.*  *R4: But personally you don’t always talk to your loved ones about it because these are very deep questions about life and life and death and whether there is a life after death and what your personal thing is. And I know I’m not, I was brought up as a Christian, I have Christian beliefs but as I’ve got older I don’t know if I believe in the supreme being. But I’ll tell you what when I first got diagnosed with my cancer I started to rethink that you know because it does.*  *Reference 4 - 0.44% Coverage*  *R1: Because one of the things when I was caring for Mum was that there wasn’t any talk about this it. I didn’t know what was happening, I didn’t know whether she’d live a year or a week.*  *R3: People don’t talk about it.*  *R1: And it was just a vacuum there was no one. So I think the issue is important but I think it’s the wording of that.*  *Reference 5 - 0.16% Coverage*  *R3: But I think that’s part of the trouble with addressing things like death it’s not talked about in our society anymore.*  *Reference 1 - 0.68% Coverage*  *R3: We don’t in general med have those strong conversations about the last year of life.*  *R1: Despite all the research that’s right.*  *R3: And you know advanced directors of care you know that should happen in the community before patients come in and if not it should happen whilst they are in hospital. But we still struggle to get that so some of it might trigger.*  *R1: But maybe it just would be another it would highlight ongoing work in that area so it adds to that body of evidence.*  *Reference 2 - 0.48% Coverage*  *R3: But I think like in 7 some clinicians might feel very uncomfortable but in fact it comes right to the core of the issue they’ve been identified by spec likely not to live for another year and many patients know they’re getting worse but they’re not having a face-to-face conversation about what is happening. So it might look confronting.*  *Reference 1 - 1.26% Coverage*  *R: Well certainly I don't find it distressing.*  *I: Yeah okay.*  *R: that question is a very subjective question, some people will not like it.*  *Reference 1 - 3.57% Coverage*  *I: with the considerate tool there has been, it has been highlighted that some of the wording on the considerate tool might be considered quite sort of slap in your face, quite heavy as such. Like for instance things like illness getting worse or time left to live or things like ahhh worried or like a burden. Do you think this wording is too harsh or are you offended by that wording at all?*  ***12:04.2***  *R: No.*  *Reference 1 - 5.02% Coverage*  *I: So with the considerate tool there was concerns around some of the wording. So for instance the wording around like sorry, like a burden or time left to live. Do you find that that’s quite confronting wording to you?*  *R: Yeah I do.*  *I: …or that doesn’t…you do find that’s quite confronting?*  *R: I do umm…the one I found more confronting, that was a bit oh my God you know…*  *I: …yeah I saw you sort of gasp a little bit there.*  *R: Yeah there’s that one. And the time left to live.*  *I: Time left to live.*  *R: Those two I ohh I never thought of meself as a burden. I do worry about you know what’s going on ahead of me. Ahhh but I guess I’ll take one day at a time but yeah.*  *Reference 1 - 2.99% Coverage*  *I: so some people have commented about the wording of like a burden or time left to live, do you find that quite confronting as well?*  *R: The time left to live I kind of went that’s …*  *I: …sort of backtracked on it?*  *R: …that’s cutting to the chase. You had told me it was cutting to the chase. Obviously the answers …would be very different whether it’s patient or carer and you don't ask that here.*  *I: You’re not stipulating it as such as who is completing it?*  *R: Yeah. And I think that’s important.*  *Reference 1 - 3.58% Coverage*  *R: Umm…yeah probably a bit blunt for some people. Where does it say about time left to live?*  *I: It’s on the last question just at the bottom here. Time left to live.*  *R: Yeah no that’s pretty poorly worded isn’t it? How would you rate your attention to what you can expect? Things like illness getting worse or time left to live. To me that’s a nothing question. I probably wouldn't answer that one.*  *I: Okay. Would you suggest changes?*  *R: Yeah delete that one.*  *I: Delete that question?*  *Reference 1 - 1.95% Coverage*  *R: and it’s good. How would you rate our attention to what you can expect? Things like illness getting worse or time left to live. Well I do worry about it sometimes but since I'm at the end of the line I'm on palliative care now. All they can do is fix the umm the pain and get me sorted and let me go. I was home for three months this time, usually I'm in every couple of weeks.*  *Reference 2 - 2.32% Coverage*  *R: I don't think they really are love. I think they’re I think what I’ve read of the questions I think they’re all pretty deserving you know and they’re sort of in the right perspective.*  *I: you don't think that because there has been feedback that so the wording ‘like a burden’ or ‘time left to live’ can be quite confronting. Do you find that a bit?*  *R: Well actually it can be but if you are positive love and you don't let it be confining it doesn’t.*  *Reference 3 - 3.93% Coverage*  *R: Yes, yes, yes. No well I can't really see anything wrong with these words love this is and actually I think it’s quite clever in a way they’ve done. Except for that one down the bottom about dying, that one is a little intrusive.*  *I: Okay so you would…*  *R: …you know…*  *I: ..prefer that question not to be in anything?*  *R: Something a little bit more gentle.*  *I: A little bit more gentle?*  *R: Like going to greener pastures.*  *I: Okay*  *R: You know.*  *I: Yeah*  *R: you’re just going to leave this world and go to greener pastures. Something like that.*  *I: Okay.*  *R: I think because some people I think it would affect. So I’ve trained myself to accept what’s going on. And I'm pretty positive and I don't try and think I try not to be negative because it’s no good.*  *Reference 1 - 5.25% Coverage*  *I: Okay. That’s been suggested as well. Really quickly with the considerate tool one other thing which has been that has been sort of ahhh sort of we’ve identified, is that some of the wording in the considerate tool some people have sort of found the wording such as ‘like a burden’ or ‘time left to live’ quite confronting. What do you think of these of that wording?*  *R: Negative. It’s all negative and yeah I don't think it really relates to the real reason why you’re doing this survey.*  *I: Okay.*  *R: You know and yeah no, if you’re going for, especially if you’ve been told that you’re in that situation and you’ve got a chronic condition with no…*  *I: …limited life…*  *R: …limited life expectancy, you don't want to see those sort of, that sort of wording. You want to sort of always want to sort of how can we do better? Not you know why are we so bad you know what I mean?*  *Reference 1 - 2.21% Coverage*  *R: I think that would depend on the person. You know like depends on your view of life and life after death and all these sorts of things come into it. So it’s quite a heavy question without seeming to be I think.*  *I: Would you find it confronting?*  *R: No I don't think so. No I think I’d like to know the answers to some of those yeah.*  *Reference 1 - 6.15% Coverage*  *R: No because no not at all in fact it was my GP who recommended to me that I should give up operations and go into palliative care which I was lucky I was on the cusp of that thing where palliative care was becoming very acceptable. I’d had an ex-wife who had died in palliative care and I’d been able to speak to her perfectly lucidly two days before she passed on and the words what other word?*  *I: “Like a burden” in Question 2.*  *R: No because well I can tell you that I felt like a burden and I understand what feeling like a burden means.*  *I: Yeah.*  *R: It’s not complex language and the other thing I would say that almost every person say in my situation or in this situation would feel like a burden to their relatives, their parents and I you know certainly I’m not morbid on this stuff but every now and then I’m thinking bloody hell they are spending so much money on me. You know it’s like a compliment in a way except that I’ve since found out also that one reason for spending money on me is that there was a good probability that it would work. But even so you know I sort of go around thinking maybe there’s someone else who’s more worthy and I think a lot of people think that. I think there are people who think to themselves I wish I could pass on now so the family wasn’t having to carry a load of costs or be so lengthened in the passing. But see I’ve been so lucky in my life in a way but my father died at 97. He was up until the last few years he was walking, well for a while just with a stick.*  *Reference 1 - 8.55% Coverage*  *I: Is there anything in the Considerate Survey that you found confronting with regards to the wording at all?*  *R: No.*  *I: No. So some people have commented that the words in Question 2 for instance “like a burden” or the words in Question 7 “time left to live” can be quite a shock. Do you find that shocking at all?*  *R: It was shocking it was.*  *I: Are you offended by that wording?*  *R: No.*  *I: No. What about the last two questions about how would you rate our attention to your affairs and how would you rate our attention to what you can expect? Do you feel that those questions are appropriate?*  *R: Yeah and I think the hospital does a good job.*  *Reference 1 - 6.71% Coverage*  *R: Yeah the thing is you are sick you know the tumour in your body, what do you expect other people to do? They do their best to help you that’s all.*  *I: Okay*  *R: Yeah.*  *I: And you don't feel like the last two questions how would you rate your attention to your affair or how would you rate your attention to what you can expect quite offensive questions at all?*  *R: I think it’s fine. I got my will I haven’t done this and umm…*  *I: …but they’re things that…*  *R: …but I told my daughter.*  *I: Okay*  *R: Ask the doctor if they can’t recover me don't delay. Just stop the breathing.*  *I: Okay so these are questions that you’ve actually sort of been looking at …*  *R: …straightforward yeah…*  *I: …yourself anyway.*  *R: Yeah I already did.*  *I: Okay so you don't find them offensive?*  *R: Yeah, the finance I already told my daughter how to. I have a will with a lawyer and they know how to get the money. Laughs After it is over they can have fortune to buy their house or something. But not for the house but make them comfortable, pay their uni fee or something.*  *Reference 1 - 10.25% Coverage*  *R: Question 7 is quite confronting.*  *I: Confronting? Okay. Because that is one thing which has been highlighted from previous interviews that we’ve conducted. Are you alright? Are you okay? So from our previous interviews that we have conducted a few people have mentioned the wording like I think in Question 2 “like a burden” and in Question 7 that “time left to live” quite confronting. So you would be in agreeance with that?*  *R: Definitely yes.*  *I: Would you like to some people have even suggested Question 6 “how would you rate our attention to your affairs” quite sort of I suppose it can be quite personal and a bit intrusive.*  *R: Oh it is it is quite personal but it’s something you have to do.*  *I: It’s something you have to do.*  *R: Something that I had to do.*  *I: Would you prefer not to see those questions on the survey?*  *R: No I think it’s something that has to be.*  *I: So even though it is confronting you believe it’s something that needs to be addressed?*  *R: Yes. I know when I was a lot younger and if anyone said you have to get a Will and I was like I don’t want to do a Will. And it’s true today that we should do one because you don’t know when you’re going to walk out there and bang you’re gone.*  *I: True. Very true. You don’t know when your time’s up.*  *R: Yeah.*  *Reference 1 - 7.35% Coverage*  *Do you find that confronting when I highlight it to you?*  *R: Not at all.*  *I: Not at all. Okay. And in addition to that some people also find that both Question 6 and 7 things that affect you in the future as well as that they find those questions are either personal or it’s a bit heavy I suppose but you want to see Question 7?*  *R: Absolutely and if they do find that discomforting I think they obviously haven’t come to grips with their situation and perhaps that would be an indication that they just need a little more help possibly.*  *I: So more time?*  *R: Yeah.*  *I: And maybe further explanation or whatever that should be?*  *R: Perhaps there could be a lead into that where people could be asked if they would like to investigate those services with someone more you know before they even did the questionnaire.*  *Reference 1 - 1.84% Coverage*  *R: Well I’m not really that well educated but I mean to say people can really delve into stuff like this you know like “affairs” you know why do you want to know about my affairs. That’s that other thing you know.*  *I: So you find that’s quite a personal question to be asking?*  ***12:41:7***  *R: Well everyone’s affairs are a personal thing because I’m an older hat in that way you know.*  *Reference 1 - 0.79% Coverage*  *R: I don’t personally but because I’m quite aware and informed so not really no. I think they need to be quite direct.* |
| **Enabling PREMs to impact clinical care** | *Reference 1 - 16.39% Coverage*  *R: Yeah ….I think you’re right it’s telling me about a point in time when I'm in hospital. And it probably doesn’t cover in regards to the progress with my chronic condition and it’s probably going to come up in a question later, I find with my chronic condition is that I'm doing these surveys but I'm not getting answers in regards to, and obviously there’s a reason for that. But every individual case is completely different. But I'm not getting answers of what I'm expecting next and that sort of can be frustrating yeah.*  *I: Okay with regards to that do you think there’s questions that are missing that you would like to see on that survey?*  *R: I would umm…*  *I: …can you think of anything like you said about your expectations would you like a question that sort of focuses on what the future might…*  *R: .,..okay yeah probably as a present situation and is possibly you know umm with myself like wanting to speak to someone about palliative care. And having that as an option in the questionnaire. Then they can come to you. I’ve got advice from people like I’ve got from the social person here that sort of gave me connections to palliative care. I'm sort of a little bit sort of shy a little bit sort of not up front in actually ringing up a person and saying “I want to speak to someone” but I prefer to be approached in regards to that sort of thing. It would be nice to have that as an option in a way. And that’s only just a slight example not a criticism and that sort of thing.*  *I: But it’s something like you would like to have that option?*  *R: Yeah, yeah and I know how busy you are and it’s a public service and I understand that. I know it’s not going to happen straight away but if I get on the list at least I can get someone to knock on the door at one stage.*  *I: Because your preference is that you would like them to approach you not having to approach them?*  ***6:55.7***  *R: Yeah, yeah.*  *I: So we actually added in the free text option at the back. So it’s just free text so that’s a section of the survey that we actually added in. What do you think about that as an option? Having the free text there? Is it something that you would use?*  *R: Yeah I prefer to be more civilised. Would you like more assistance in, as an example of …social, dietician, palliative care etc. etc. As an option circling option then writing it down. And that’s you know embarrassment in regards to hand writing skills and embarrassment to spelling and the embarrassment of you don't know what’s available. Education is probably a big thing, you don't know what is available through Queensland Health. Obviously …*  *I: So having that as an option of these are the services that we can offer you…*  *R: …,yeah do you feel like you need more assistance.*  *Reference 2 - 3.62% Coverage*  *R: …I think keeping it under sort of twenty is probably a good idea even under fifteen is a good idea. But as I said to you before on both these questionnaires I’d like to get, instead of writing that information out, getting more details. And more generalised like I said you know would you want any more assistance from palliative care from dieticians etc. Just as a tick box than writing it down because as I said you don't know what’s available through Queensland Health and all that sort of thing. I didn't know a thing about palliative care until recently and I’ve been in the system for four years.*  *Reference 3 - 3.57% Coverage*  *I: So that is one of the things which has been highlighted is potentially using the survey that we do use as a tool to see where there might be gaps as a quality improvement type exercise to a certain extent. But also to use it as a sort of discharge checklist of is there anything else that we need to address or would you like us to assist you with. So that I suppose falls in line very much with being aware of what is potentially out there to help you in the future.*  *R: Yes and giving those options as I said but yeah.*  *I: So you believe that would be something…*  *R: …oh definitely….definitely.*  *Reference 4 - 2.70% Coverage*  *R: …no umm…I think going back to the point in regards to umm you know asking the question of if you do require more care, possibly something you know. If you do require and I'm not barking on (??) but there are other like social security things and all that sort of thing. I think maybe websites or that sort of information so we can do research if we care to. Might be beneficial, again this is only a personal thing but it could be beneficial. Yeah.* |
| **Feedback about the icons** | *Reference 1 - 0.90% Coverage*  *I find it very helpful that it’s got all these pictures, it’s easy to understand.*  *Reference 1 - 0.38% Coverage*  *R: Yeah I like those.*  *I: You like the icons?*  *R: Yes.*  *Reference 2 - 1.63% Coverage*  *R: They just sort of make it a little bit more interesting don’t they?*  *I: Okay.*  *R: I mean the thermometer and the heart and this here I mean every one of the icons fits what the questions are about. Yeah no I think it’s good.*  *Reference 1 - 1.39% Coverage*  *I would put the icons to the left and then the numbering system. And I’d have it left the line…that’s probably my only other thing from a formatting perspective.*  *Reference 1 - 3.38% Coverage*  *I: The question itself do you believe it’s a good question to be asking?*  *R: Yes I do. I believe it’s a very good question because I mean people have got to get I mean I’ve made a Will. I mean granted when I made the Will I had money but now I haven’t but that’s beside the point. You know it’s you’ve got to make it easier for other people to you know I’m going to say no he can’t be buried there. They’re going to walk over and say well we’re his daughters here he can and I thought you can’t do that you must have it written down on paper even if it’s a sheet of paper.*  ***30:33:9***  *I: Yep. But you feel that’s a good question to be asking?*  *R: I do feel and I mean he’d be only one of many I should imagine that wouldn’t do it.*  *I: Yeah. And you’re not offended by any of the wording in the Considerate Tool?*  *R: No not at all.*  *Reference 1 - 1.10% Coverage*  *These little images here like are fluff. And take up unnecessary space to…*  *R4: I think they’re cute. Laughter*  *R: I don't….*  *R4: …I think they’re cute because if they had it on the right hand side it would almost work as a bullet point versus being on the right hand side which is just a picture.*  *R: …yeah it would be better if they were a bullet point not just a little…*  *R4: …yeah like as in this question is this thermometer. So I think if you …*  *R: …I did like how they…*  *R4: …put…*  *R: …put a calendar on the last one. You know just to try and see who actually liked…*  *All laugh and talk at once*  *R: …as every day goes past you can tick those boxes off.*  *R1: Like an advent calendar…*  *R: …that’s right. Getting close to the end of life.*  *Reference 1 - 3.60% Coverage*  *R: that’s just a bit more I think the symbols umm….as well as the questions are good because you can sort of look at it and know what it’s or partially know what it’s about before you even start reading.*  *I: Okay so you think that having the symbols on the side of the considerate tool relates to the question and therefore it’s easier to…*  *R: …yeah you’ve got the question sort of half of that in your head yeah.*  *Reference 1 - 1.94% Coverage*  *I don't know what these are for but they are very confusing.*  *I: So that, you’re pointing just because this is the interview sorry, recorded, so you’re pointing to the icons on the side of the considerate tool.*  *R: Yes*  *I: So you find they’re quite confusing that they’re just what’s the purpose of them type thing?*  *R: exactly.*  *Reference 2 - 1.36% Coverage*  *R: I mean that’s marginally better as much as it’s smaller than the other one. And that’s fine. But I think the icons I thought the icons were going to somehow relate to the bad, good, very good and they don't. They are just pretty.*  *Reference 3 - 0.70% Coverage*  *R: Well for the reasons I mentioned I think this all looks very silly.*  *I: Yep so that’s the icons on the considerate.*  *Reference 1 - 3.35% Coverage*  *I: Because we have had feedback already that wording such “like a burden” or “time left to live” is a bit sort of like not in your face but quite bold as such. What do you think about that wording?*  *R: No I think that you know I don’t think there’s anything in there that I’d be …*  ***08:09:0***  *I: Upset by.*  *R: No.*  *Reference 1 - 0.50% Coverage*  *R: Yeah. I think they might be felt as a pressure and also as a substitute for a device and what do they call them emojis.*  *Reference 1 - 4.92% Coverage*  *R: It’s a little bit primary school.*  *I: Primary school? What do you mean by that?*  *R: Just the graphics?*  *I: The icons. Do you find it’s quite cramped because that has also been sort of brought up that there’s a lot of information on one page and the questions sort of might potentially flow into each other?*  *R: I didn’t think it was too bad. This is easier to read obviously.*  *I: So the Commission Tool is easier to read?*  *R: Yes.*  *I: Is that because of the layout with the boxes like that space underneath?*  *R: Yes it’s just the formatting.*  *Reference 1 - 0.41% Coverage*  *R2: Take away the pictures and move that up.*  *I: You don’t like the pictures?*  *R: No.*  *Reference 1 - 1.08% Coverage*  *R: And it stands out that’s good and it’s got the medical things and the shaking hands and all that stuff.*  *I: Okay so you like the icons on the considerate tool?*  *R: Yeah* |
| **Feeling there is inequity across diagnoses** | *Reference 1 - 7.08% Coverage*  *But it does vary. If you’re getting treated on Level 7 with cancer you’re treated so much differently than if you’re a renal patient.*  *I: Okay*  *R1: The..*  *R: ..the funding for renal…*  *R1: ..the funding is….*  *R: ..is disgusting…*  *R1: …just horrific.*  *R: ..in this hospital.*  *R1: Umm and the food is different. Morning tea for patients is different, they’re limited financially with budget restrictions on how much they can give a patient. Whether it’s one piece of cheese and one cracker. You have the cancer patients coming up …*  *R: …and they laugh.*  *R1: …and they laugh. And they’re like “Oh no I'm not having morning tea here when I can get this, this, this and this down in oncology”. And just makes your illness seem not as important or not as serious.*  *I: Yeah*  *R1: Whereas you are just dying over a longer period of time is the only difference. And the, it’s heart breaking watching patients older patients having to ask …*  *R: ..every week…*  *R1: …a food service person just for an extra piece of cheese. And a cracker and being refused. It’s like this person is sitting on dialysis and because of a list on a piece of paper that you’ve got with all these people’s names and what they can only have. These patients are only allowed to have one piece cheese…*  *R: …two crackers…*  *R1: And one cracker, packet of crackers which has got two crackers in it. That’s it. No more because the list says so. And it’s just like give these people a break like I don't eat them so it doesn’t bother me but it’s heart breaking. Listening to a seventy year old ask ‘Can I have an extra piece of cheese’ you know. It’s like how degrading is that? This man has probably fought for our country for all they know and they won’t give them one extra piece of cheese and some crackers. It’s like you have to be kidding me.* |
| **Formatting** | *Reference 1 - 1.61% Coverage*  *R: Just the formatting is…*  *I: …the formatting…*  *R: …isn’t difficult.*  *I: Okay is there anything confusing or poorly worded within the survey at all that you found?*  *R: Umm…no. That’s fine.*  *Reference 2 - 1.39% Coverage*  *I would put the icons to the left and then the numbering system. And I’d have it left the line…that’s probably my only other thing from a formatting perspective.*  *Reference 1 - 0.89% Coverage*  *So I think the design of this is more user friendly definitely and for a person who’s perhaps in more pain than not this is definitely much more user friendly.*  *Reference 2 - 0.55% Coverage*  *R2: It’s quick and well the lay out of it and you know you can go through it really quickly I think.*  *Reference 3 - 3.59% Coverage*  *R2: I think this lay out with the tick a box underneath.*  *I: So that’s looking at the considerate tool?*  *R2: I think is easier to follow and how [name] was just saying the little quick explanation of the type of …*  *I: So having that sort of not prompt as such but those couple of words under each question would be helpful?*  *R2: Yeah.*  *I: Is there anything else?*  *R: I think the same thing applies here if you want to explain some of your answer you know obviously this goes into a big study that kind of provides some you know research but whether you presumably you know a little bit about the patient, the study knows a little bit about the patient.*  *Reference 1 - 5.97% Coverage*  *R: But this one here like that one there it was I had to go over the questions and I had to vision what the question was saying. I had vision myself and I had to vision Mum and I came back to that’s why I put always for that question.*  *I: And you were just pointing at the Commission tool then when you explained that. That’s just for the tape recording sorry. So is there anything unclear or confusing about the lay out?*  *R: No they’re both good don’t get me wrong they’re both good but this one here was a lot better so like I said I just read that part under the word.*  *I: So you preferred the lay out of the Considerate survey that they did that explanation underneath.*  *R: Yes.*  *I: You kept talking about that you had to envision the questions so having that sort of explanation underneath helped you in that?*  *R: Yeah then I could understand it.*  *I: Okay.*  *R: It’s just that with this one here I had to sort of put Mum in the picture and put myself in the picture as well and I had to like when she was doing stuff like the same again for this one here I put the same sorts of scenarios to this one.*  *Reference 1 - 1.04% Coverage*  *R1: So I think I agree, I think this was also like a bit more user friendly.*  *I: The considerate tool?*  *R1: Yeah how it is displayed and I guess the specific information you get from here you can use. Whereas I don't know like if question four, I felt cared for, like if they circle …I guess you’d have to, you’d need them to elaborate more and be like “Okay well why did you not feel, why did you say never or why did you say rarely”? Whereas this is like quite specific to, it’s not as vague as this I guess I would say. Which is why I feel like it’s user friendly and it’s quicker and I think that’s what I feel like that’s how you get more numbers. If it’s something that’s quick and ticking a box.*  *Reference 2 - 0.93% Coverage*  *R: I’ve got a bit of dyslexia and I find it really messy.*  *All at once*  *R2: …I think that would be a solution by as (?) had said that if it was just not one straight under each other, if they had a text box here this becomes very clear.*  *R: It breaks it up.*  *R2: …and you don't come together. I think that could additionally make it a lot better if not, putting it to the side makes no big difference actually.*  *R4: I like the questions.*  *R2: Yeah I actually totally agree about the layout. I found it busy and didn't like it as soon as I looked at it but when I took the time to read it that’s what, that’s why I prefer this.*  *Reference 3 - 0.66% Coverage*  *R2: ..it’s messy and I even missed, when I first read it if I'm being honest I was like “Oh these questions I feel like are just going to like get a bit more of like a culture response”. In the sense of what’s the culture of this ward or place I'm in. But then when I looked closer and saw that the prompts were there for the types of information we’re trying to derive, that’s why I prefer it. But it’s definitely visually too busy and messy.*  *Reference 4 - 0.34% Coverage*  *R1: I was thinking even if you broke it up with different, more colours, the question in a different colour. Like I know it’s bold but like it takes a lot to actually figure out this is one question and this is where I answer like…*  *Reference 1 - 0.36% Coverage*  *R1: Just physically this one’s easier to look at.*  *I: Okay the Commission is easier. I’m going to keep using the word for our recording.*  *R3: To engage me I found this one much easier.*  *I: The Commission layout is better.*  *R1: This one’s too busy.*  *I: So the Considerate is too busy.*  *Reference 2 - 1.67% Coverage*  *R1: The issue I have with just handing these out is I think patients need to be prepared. Communicated in some way about what these surveys mean because I don’t think my mother would have done this properly and she hadn’t lost capacity or anything. I have a problem with some of the wording in this one even the way the questions are framed probably encourages an honesty more this one sort of pre-empts a certain sort of response. And my mother would have been tempted to please given what she thought.*  *I: You think the Commission is trying to pre-empt?*  ***34:36:7***  *R1: It’s better set out but those questions they’re worded in a positive statement “my individual needs were met”*  *R3: My views were listened to.*  *R1: To get the patient to agree, agree, agree and get away home. But this one my mother would have probably had issues with “how would you rate our attention to your affairs” and she would have said what affairs [name] what do they mean by that?*  *R3: These surveys need to be explained to patients.*  *R5: How dare they want to know about my personal affairs and how much money I’ve got in the bank it leads itself to …*  *R1: The vagueness of “how would you rate our respect for what matters to you”.*  *R2: It says Will, finances or advanced directives.*  *R3: We’re not helping you [name of researcher].*  *Reference 3 - 0.42% Coverage*  *R4: I think the problem with this is the design but I like the questions with a few of the questions being changed.*  *R3: It’s too busy.*  *R4: But that’s a very busy format.*  *R3: It’s busy isn’t it it’s too busy.*  ***37:20:6***  *R4: But that’s a very buy format I think someone could design a different form that could look very much like that.*  *Reference 4 - 0.13% Coverage*  *R3: That’s what my thoughts were you’re more likely to engage with something simple rather than busy.*  *Reference 5 - 0.11% Coverage*  *R3: Not that it’s not right but it’s very busy which is an automatic disengagement.*  *Reference 1 - 0.65% Coverage*  *R1: I like the way you just have to circle or indicate. I mean if I had the survey it doesn’t take long obviously if someone’s got cognitive impairments it’s going to take a bit long and that’s another compounder which you’ve got to work out. But I think giving this so it’s really important that people can write something more because each question may stimulate something else they want to say. Or it could be have you I mean they can say positive things to.*  *Reference 2 - 0.29% Coverage*  *R2: I think the layout I don’t like I think it looks messy.*  *I: The Considerate?*  *R2: I think it looks very busy for only having what 7 questions or something.*  *I: It is 7.*  ***30:12:6***  *R2: It looks very busy.*  *Reference 3 - 0.16% Coverage*  *R1: But if you actually spaced it out like if you put that over two pages it would be really attractive to look at.*  *Reference 4 - 0.77% Coverage*  *R1: And it is actually quite good to have it underneath. I think it’s quite hard when people going to fiddle and circle you know whereas someone can easily just tick a box whether you change it somehow there or give more space.*  *R2: I think the layout on here is good if you had some spaces in between questions.*  *R1: That would fit all on one line if you just widened it and then you could spread it out.*  *R3: I mean it’s good you know the physical problems it’s not just pain which we’ve talked about already which makes it really clear to the person.*  *Reference 1 - 3.59% Coverage*  *R: I think …umm…I don't know I think they’re both much of a muchness. Umm…but see yes they’re all much, I didn't see the little boxes underneath. Oh well see look see the boxes, I didn't see them. Tick them lovely. Anything that’s easy.*  *I: You like the ease?*  *R: I like the ease and they’re not looking for a ten mile explanation , tick, done, tick done. Yeah. Because in my expectations would be I’d have to write something in there.*  *I: Okay.*  *R: That’s what it looks like to me.*  *Reference 2 - 3.57% Coverage*  *I: So is there anything I mean you sort of touched into it a little bit too before, because my next question is about if there’s anything confusing or unclear about the format. But you were saying that you preferred the tick boxes nice and simple. And you did like the explanation of the considerate tool. And that the format of the commission tool having that space with the implication that you might need to expand further, you didn't like so much.*  *R: Yeah I didn't like that.*  *Reference 1 - 1.97% Coverage*  *R: Whose perspective? Because you said that they’re completing it on behalf of their loved one. So it’s not them completing it, who is this for? It’s not for the caregiver, it says people who are ill or for their caregiver. Not it’s for this person who is ill but the caregiver is actually completing the form. So that’s very confusing.*  *Reference 1 - 3.32% Coverage*  *I: If you were to fill this survey out would you find it just by the formatting and so forth would you find it an easy survey to complete?*  *R: I think so. It looks pretty basic to me.*  *I: Okay. There’s nothing confusing or poorly worded.*  *R: No.*  *I: You understood all the questions quite clearly?*  *R: Yep.*  *Reference 2 - 2.65% Coverage*  *I: Do you think that’s something you might not use you prefer having that bulk text free answers at the back?*  *R: No I reckon it’s a good idea because otherwise you don’t know what’s happening do you? You’re saying never and you don’t know why.*  *Reference 1 - 1.46% Coverage*  *R: Umm no. No it’s very, very easy yeah. No that’s fine no worries there. A lot of these, this is a good thing, I think my last survey I got from you guys is like this and it’s a good thing to do online because you just highlight it, it’s great.*  *Reference 2 - 1.86% Coverage*  *R: oh probably just skimmed through it too quickly but no I don't have umm…I just find the formatting because of so close I do find it hard to sort of concentrate on that line and do the answer. More so than because of that, and that’s you know again from reading abilities and all that sort of thing. And yeah.*  *Reference 1 - 3.95% Coverage*  *R: Well on first inspection yes it was a bit confusing. But it didn't once I started in I was pretty right yeah.*  *I: Okay so and what about the layout for the commission tool did you find that there was, was that confusing? Or you felt it relatively easy to flow through?*  *R: Mostly I found that a bit more or less, more hands on size version I suppose rather than a wordy version.*  *I: Okay*  *R: I found that perhaps could develop into a wordy sort of thing. Whereas yeah. But like yeah for a person ahhh….*  *I: Is there anything that you would potentially suggest you would change in either of the tools?*  *Reference 1 - 1.68% Coverage*  *R: Again I’d just if I had a tertiary education I’d say if I was a patient with a tertiary education I’d say no but given that I’ve mixed with a lot of very ordinary people I’d say some of the language is too clinical. Like individual needs, views and concerns when a need could not be met is not because sometimes it’s the need is not exactly me asking for tea in the afternoon or even it’s also unexpressed needs.*  *Reference 2 - 2.25% Coverage*  *: I think 12 even the lay out of that I’m less and less liking.*  *I: Okay and that’s the Commission Tool.*  *R: I much prefer these things.*  *I: So you prefer the boxes to tick?*  *R: Under the question.*  *I: Yeah. There’s enough questions there do you think it covers everything that it should do there’s nothing you’d like added or taken away?*  *R: I’m not sure. Let’s say if you can think of other things to ask that I don’t I’m in two minds I don’t think you necessarily need more but I think 10 questions would convince people of the sincerity of the survey.*  *Reference 3 - 1.53% Coverage*  *R: Yeah. I think they might be felt as a pressure and also as a substitute for a device and what do they call them emojis. But anyway yeah and obviously you know I prefer this one but I really would recommend the possibility of putting some individual comment as well under each question. Not necessarily only three words but something like is there a phrase you like to use.*  *Reference 1 - 6.57% Coverage*  *I: Okay. What do you feel about the layout of the surveys themselves?*  ***11:34:2***  *R: No they’re pretty good.*  *I: There’s nothing confusing?*  *R: No.*  *I: No. So some people have said with the Considerate Tool that it does look quite busy like there’s not distinct definition between the questions. Do you feel that at all [patient name]?*  *R: Well it could just be spaced out a bit more and maybe some of the free form answer.*  *I: So free form a bit of text underneath?*  *R: To allow you to make comments.*  *Reference 1 - 0.99% Coverage*  *I: Okay. Is there anything in the survey that you found confusing or poorly worded?*  *R: No.*  *I: No? So it was all very clear?*  *R: Simple, straight forward.*  *Reference 1 - 0.42% Coverage*  *R: Oh definitely.*  *I: Yeah.*  *R: Very easy to fill out.*  *Reference 2 - 0.87% Coverage*  *R: And I’m sure somewhere there you’ve got to be able to answer one of those. It’s just laid out really good.*  *Reference 3 - 4.79% Coverage*  *I: What do you think about the length of the survey? So in the Considerate we’ve got 7 questions and in the Commission Tool we’ve got 12. Do you think that’s enough questions or would you prefer to see more or less?*  *R: I think it’s set out well actually.*  *I: Okay. Do you like the formatting of both of the surveys or do you prefer the formatting one more than the other?*  *R: I prefer the way it’s done that.*  ***14:45:6***  *I: So you like the Considerate Tool and the way it’s formatted?*  *R: Yes.*  *I: You don’t find it too busy with too much information that gets a bit missed at all?*  *R: No to me it doesn’t.*  *Reference 1 - 4.92% Coverage*  *R: It’s a little bit primary school.*  *I: Primary school? What do you mean by that?*  *R: Just the graphics?*  *I: The icons. Do you find it’s quite cramped because that has also been sort of brought up that there’s a lot of information on one page and the questions sort of might potentially flow into each other?*  *R: I didn’t think it was too bad. This is easier to read obviously.*  *I: So the Commission Tool is easier to read?*  *R: Yes.*  *I: Is that because of the layout with the boxes like that space underneath?*  *R: Yes it’s just the formatting.*  *Reference 2 - 1.06% Coverage*  *I: Okay. So you prefer the formatting on the Commission Tool over the Considerate Tool?*  *R: Yeah it’s just cleaner.*  *Reference 1 - 1.00% Coverage*  *R: We didn’t read it that close not really no. No it’s just pretty basic you know like what it is “did I receive pain relief that met my needs”. Well I didn’t ask they asked and I received yeah quite good.*  *Reference 2 - 1.37% Coverage*  *R: Well I mean to say it just says here “how do you rate your attention to your physical problems” and then it’s got a question mark and then it says “things like dry mouth” instead of just putting it underneath you’ve got to go one dry mouth, two you could do it that way I don’t know.*  *Reference 3 - 0.89% Coverage*  *R: But I mean say see this one here as I was reading it and then you might miss the underlining see the underlining and that’s got to be highlighted. That would have to be highlighted.*  *Reference 4 - 2.36% Coverage*  *R2: How would you rate this number one, two dot, three dot.*  *I: So after each number you think that they should have the full stop so it’s very clear that that’s the question?*  *R2: Yeah because that just looks like I.*  *I: Like I how would you.*  *R2: There’s no dot after it. So if you took the pictures away then you could move that over.*  *R: But like this you’ve got your dots down the bottom.*  *R2: Yeah dot points.*  *R: Dot points. You know you could sit here for hours and do this you know.*  *Reference 1 - 1.97% Coverage*  *R: Yes but it doesn’t actually tell you how to respond. But that’s given you that but it doesn’t say whether or not you need to circle it or whatever.*  *I: So a bit of direction I suppose.*  *R: Yeah. And even if it’s just a tick a box sort of thing. But other than that it’s straight forward.*  *Reference 2 - 2.04% Coverage*  *R: In terms of ease of reading it is not very good at all. That’s the other part of the work that I do. I mean it’s not designed to actually be able to flow the content at all. I mean everything’s got numbering going right through the middle of the thing it’s just crazy so it needs a really good edit.*  *Reference 3 - 5.46% Coverage*  *R: Well to start with the whole thing’s actually back to front in terms of my understanding. I mean you’ve got icons down the right-hand side which is where they are don’t mean anything. If you actually put the icon on the left it gives you some indication and a visual clue as to what it is the question’s going to be about. The other thing is you need to I mean to start with align everything to your left and people shouldn’t use centred on any survey you have at all. And making sure that there’s a clear differentiation between the question, the explanation component and then where your responses need to go. And then you’ve got the second question drops into the right so it just looks like it’s just a continuing piece of text there’s no visual clues as to where you need to go so it’s pretty appalling I think.* |
| **Brevity and ease of completion is important** | *Reference 1 - 3.00% Coverage*  *I: what specifically about the set out? Is it the questions? Is it the formatting…*  *R1: …it’s easy….*  *R: It’s a lot easier. To me that’s a lot easier to…*  *R1: ..that’s quick…*  *R: …to understand and do.*  *R1: ..can I say like a lot of the dialysis patients would easily be able to…*  *R: …to do that.*  *I: Okay*  *R1: …skim through that and do that and be happy to hand that in.*  *R: Yeah*  *R1: I'm only speaking from what I know. But when there’s a section to write an answer, a section to write an answer, a section to write an answer for every question …*  *R: …See this one on here…*  *R1: ….it’s too much.*  *R: …Sandra? Has got always, mostly, sometimes, really, never, didn't apply. This one is much better it’s got very bad, bad, good, very good doesn’t apply.*  *R1: Yeah.*  *Reference 2 - 1.34% Coverage*  *: Okay what about the length of questions? So the considerate tool is seven questions long and the commission tool is twelve questions long. Do you think …*  ***20:24.5***  *R: …I think that’s enough because on the back there you can still write any other extra feelings you’ve got.*  *I: Okay so that’s the considerate tool that you’re talking about….*  *Reference 1 - 5.69% Coverage*  *I: So when you chose to actually complete that survey today did you find it quite easy to complete?*  *R: Yes.*  *I: There was nothing in the wording that was confusing or it was poorly worded you understood all of the context?*  *R: Very easy.*  *I: Okay. Is there anything that you could suggest to like any suggestions with regards to the survey itself?*  *R: In what context?*  *I: So with regards to formatting. Is there anything around the formatting that you didn’t like?*  *R: No I think it’s pretty straight forward which is important.*  *Reference 2 - 3.18% Coverage*  *I: Okay. And what about the length and numbers of the questions. So we have 7 questions in the Considerate Tool and there’s 12 in the Commission Tool. Do you like those numbers of questions? Do you think that’s too many or too less?*  *R: No it’s good. I had no problems filling out the 7.*  *Reference 1 - 0.35% Coverage*  *R: No I don’t think I’ve found it so far this time or last time no it’s pretty clear cut.*  *Reference 2 - 0.37% Coverage*  *R: Very, very, very straight forward. They can’t misunderstand anything just tick the boxes.*  *Reference 3 - 1.70% Coverage*  *R: Yeah. But this one as I said I look at that and it’s if that was a field trip thing for high school students who were half paying attention sort of thing they could go very bad, good, good, good, bad and that’s it it’s all done. But if you put two or three lines in the middle and everyone had the opportunity of passing a comment I think ticking the boxes I suppose you’re doing the same thing there but you’re circling them aren’t you?*  *Reference 4 - 0.59% Coverage*  *R: It will give you an idea anything less it would be a case of just not adequately covering all topics. No that one’s alright and that one’s not bad.*  *Reference 1 - 4.07% Coverage*  *R: About it? Well they’re questions that are easy to answer and respond to that’s for sure. And I mean they’re reasonable they’re not asking too much of anybody.*  *I: Okay. There’s nothing confusing or poorly worded in the questions?*  *R: No definitely not confusing.*  *I: And there’s no questions that you had to sort of read a couple of times because you didn’t quite understand what they meant?*  *R: No. The only one that I sort of double read was the one about unexpected harm or distress because I thought oh gee I hope I never have to worry about that sort of thing.*  *Reference 2 - 8.02% Coverage*  *Yes definitely I find the formatting a bit too close.*  *I: Okay no problems.*  *R: I think a double space between each of the questions would have been better.*  *I: Alright. Some people have mentioned that even putting some lines under to make …*  ***12:21:8***  *R: Lines under yeah just to make a division because it all runs into each other.*  *I: Okay.*  *R: That’s a bit picky I suppose.*  *I: No not at all you want it to be visually so people aren’t going to be oh okay and also that people aren’t going to get confused by it and so forth.*  *R: I like that format far better.*  *I: So you prefer the Considerate format better than the Commission?*  *R: Oh yes because I mean this one are they expecting you to write something there or just or you’re supposed to just circle that one?*  *I: Yeah it’s definitely circle the response that applies to you but I’m sort of I was under the assumption that potentially and that box that space that’s shaded underneath it’s an area that you could write into it I suppose. Exactly if that was their intentions or not I’m not too sure.*  *R: Yeah but then on this one you could put …*  *I: You could put additional.*  *Reference 3 - 0.93% Coverage*  *R: No I like this one I think it’s concise.*  *I: So the Considerate.*  *R: Yeah I think it is. People are to the point on this one.*  *Reference 1 - 1.54% Coverage*  *R: Umm…yeah I think the seven questions cover enough of the care plan that you would get in hospital. And the free text is a good thing to have. And the last one is fine as well.*  *Reference 1 - 0.55% Coverage*  *R2: It’s quick and well the lay out of it and you know you can go through it really quickly I think.*  *Reference 2 - 0.93% Coverage*  *R: So the seven is much easier to use I think. So I think from the perspective of the patient if it captures enough information than that’s probably better for them.*  *Reference 1 - 0.48% Coverage*  *No it’s just I read the question I had to think and debate and vision the actual question.*  *Reference 2 - 0.78% Coverage*  *I: With regards to that it was more about the ease of the questions and you pointed to the explanations underneath the questions as well.*  *R: Yes.*  *Reference 3 - 0.60% Coverage*  *No the length was fine, everything was all fine it like I could have answered 50 questions you know what I mean.*  *Reference 1 - 0.94% Coverage*  *I: Okay great. Does anyone else have any comments? What their thoughts are?*  *R1: I think it’s a good amount of questions. I think any more than this yeah you know probably wouldn't get a great yeah engagement or compliance just completing it.*  *I: Engagement from nurses or from the patients?*  *R1: Both. Probably more staff is where I'm thinking of just because they’re so time poor. I think that if the patient has got the capacity to complete this they probably would and yeah of course a couple of extra questions I don't see that being a huge barrier for a patient. Providing that they’ve actually got the ability to do it.*  *Reference 2 - 0.13% Coverage*  *no one is going to want to fill it out if it keeps getting bigger and bigger and bigger.*  *Reference 3 - 0.46% Coverage*  *R3: I think the same, it’s short which is important. Patients or staff getting bombarded with questionnaires usually other things as well, whether it be the quality of food that they’re getting or you know they get usually multiple surveys when they’re admitted. So keeping it short is very important I think.*  *Reference 4 - 0.34% Coverage*  *R3: Certainly nothing that screams it’s concerning or you know I think that most questions I think people would be able to find fairly easy to answer. Like whether it’s an always or never I think it’s fairly, it’s pretty clear cut.*  *Reference 1 - 0.16% Coverage*  *R1: I think the fewer questions you’ve got the better to I think sometimes if you have too many questions it puts people off.*  *Reference 2 - 0.04% Coverage*  *R3: No the briefer the better.*  *Reference 1 - 1.03% Coverage*  *R: So that’s very quick, that would be alright. Even 12 if it’s a multi-choice question I mean it’s not that but I would have said that’s okay. I mean ESAS is not too bad but the distress thermometer obviously people use it it’s easy but the one with the 50 different sort of worries me a bit oh your patient’s so minor but that has so many things that you’ve got to click. I mean one of our problems has got that the ESAS and ECQ5D regularly and then using some sort of maybe QLQ, RTC, Q30 that’s only been in for a few months but then once you have that plus the disease specific module then all of a sudden you’ve got 50 questions for that. So you do have to get if you’re going to ask regularly you need fairly committed patients.*  *Reference 1 - 1.12% Coverage*  *R: Yeah definitely. Nobody likes to fill forms. But this is twelve questions this is seven questions that’s the right number.*  *Reference 1 - 1.98% Coverage*  *I: So do you feel this, the considerate tool might be a bit more personal than the…*  *R: …yes…*  *I: …commission tool? Is there anything else, are there any other reasons why you prefer it?*  *R: No I just…so easy to answer you know.*  *Reference 1 - 1.08% Coverage*  *R: Oh yeah, yeah. Because the answers are there you don't have to like I don't have to think about it you know because they’re just lovely people.*  *Reference 2 - 0.81% Coverage*  *R: No. No. Pretty good survey actually. Normally you get all the, all the long words and that no that’s good.*  *Reference 3 - 2.85% Coverage*  *I: What about the length of the surveys? So the considerate tool is seven questions and the commission tool is twelve questions. Do you find that that’s okay, would you like less, would you like more?*  *R: No that’s just about it. Like when you start getting into you know miles and miles of questions you think “Oh God how long is this going to take”? But no I like this one better.*  *Reference 1 - 0.58% Coverage*  *R: I'm assuming I could just circle each of the answers or underline or something, yes, yes I do.*  *Reference 2 - 3.66% Coverage*  *R: Can’t do it too short then you’re not going to get the depth of knowledge that you need. And I think most of us should be able to cope with twelve questions. Especially if you got rid of this and put them under here.*  *I: Yeah. So that is getting rid of the free text on the third page of the commission tool and putting some sort of text box underneath the questions themselves.*  *R: Which the only thing that does do is take away the ability to be general. If there’s something somebody wants to say that doesn’t relate to your…*  *I: …to those questions…*  *R: …questions. So maybe you still need one free floating box.*  *Reference 1 - 4.25% Coverage*  *: So is there anything confusing or unclear about the layout exactly? You mentioned about the ticking of the boxes, do you not like that option? Do you prefer to be able to circle a box?*  *R: Well I prefer to be able to add comments if I need to. There’s nowhere there to, only on the back which you know…*  *I: Okay so you like to be able to be given the choice of adding comments underneath the specific questions but also…*  *R: ..yes I think that…*  *I: …be able to have that option of adding anything else that’s not covered in the questions in a free text option at the back?*  *R: Yep.*  *Reference 2 - 2.97% Coverage*  *R: Oh I think it’s a bit too brief. I'm not quite sure what the thrust of the whole thing is.*  *I: Okay so that’s the considerate tool that you’re talking about is too brief.*  *R: Yeah*  *I: So you prefer just having that option of more questions to cover everything?*  *R: yeah I mean if you’re going to do this sort of thing you may as well do a more comprehensive one.*  *I: Okay.*  *R: Just a tick and flick (??)*  *Reference 1 - 1.64% Coverage*  *R: pause Well maybe instead of putting ‘how would you rate your respect for what matters to you’ maybe if you just say you know ‘you just appreciate someone coming and shaking your hand. Or just saying a kind word to you’ love. And that would be, fit that wording somewhere down that would be quite pleasant I think.*  *Reference 1 - 1.59% Coverage*  *R: No I think that’s a good length you don’t want to sit there for ages and go through a hundred things.*  *I: Yep.*  *R: No I think that’s pretty good.*  *Reference 1 - 3.62% Coverage*  *R: …I think keeping it under sort of twenty is probably a good idea even under fifteen is a good idea. But as I said to you before on both these questionnaires I’d like to get, instead of writing that information out, getting more details. And more generalised like I said you know would you want any more assistance from palliative care from dieticians etc. Just as a tick box than writing it down because as I said you don't know what’s available through Queensland Health and all that sort of thing. I didn't know a thing about palliative care until recently and I’ve been in the system for four years.*  *Reference 1 - 2.25% Coverage*  *: I think 12 even the lay out of that I’m less and less liking.*  *I: Okay and that’s the Commission Tool.*  *R: I much prefer these things.*  *I: So you prefer the boxes to tick?*  *R: Under the question.*  *I: Yeah. There’s enough questions there do you think it covers everything that it should do there’s nothing you’d like added or taken away?*  *R: I’m not sure. Let’s say if you can think of other things to ask that I don’t I’m in two minds I don’t think you necessarily need more but I think 10 questions would convince people of the sincerity of the survey.*  *Reference 1 - 1.25% Coverage*  *R: I don’t think questionnaires benefit from having too many questions. So it’s probably okay.*  *Reference 1 - 4.79% Coverage*  *I: What do you think about the length of the survey? So in the Considerate we’ve got 7 questions and in the Commission Tool we’ve got 12. Do you think that’s enough questions or would you prefer to see more or less?*  *R: I think it’s set out well actually.*  *I: Okay. Do you like the formatting of both of the surveys or do you prefer the formatting one more than the other?*  *R: I prefer the way it’s done that.*  ***14:45:6***  *I: So you like the Considerate Tool and the way it’s formatted?*  *R: Yes.*  *I: You don’t find it too busy with too much information that gets a bit missed at all?*  *R: No to me it doesn’t.*  *Reference 1 - 1.04% Coverage*  *R: Well I couldn’t really say what other questions to ask at this stage of the game. Some surveys you go there and you get 40 questions and it’s so confusing and it’s some of the questions just contradict the other.*  *Reference 2 - 5.28% Coverage*  *R2: We know what they’re feeling.*  *R: We do we’re pretty well tele connected.*  *I: You’re in tune with one another?*  *R: Tele connected type of thing well we’re not but we are.*  *R2: Pathetically connected.*  *R: Pathetically.*  *I: So you believe that we should be having a set of similar questions for carers?*  *R: Yeah.*  *I: Okay.*  *R: Yeah why not you’ve got to ask the question as you say but there again you’ve got to have the people.*  *R2: And a lot of the time when they’re sick they’re not thinking straight.*  *R: They’re not thinking at the right level field no. I’ve been at home sometimes and I’ve been in bed you know just lying there you know just going through the motions and you know I couldn’t sit here with you doing this or lie there with you. I’m not being rude to you or anything but I’d end up telling you to move but that’s the way it is it’s just not right. But me this is as I said today is the best day I’ve had in a hell of a long time. Everything’s clear kind of you know well up to a degree I think. I’m getting better and I feel good.*  *I: Okay.*  *R: To do surveys type of thing.*  *Reference 1 - 0.45% Coverage*  *R: No I think it’s good I mean if you can keep it to two pages great.*  *Reference 1 - 1.92% Coverage*  *R: Well I did for me because ahhh all the questions that were asked have been answered you know. Particularly the last few days but as I say I’ve been here before, once before I was here four days and I was treated brilliantly.*  *Reference 2 - 0.53% Coverage*  *R: Umm…looking through survey…no it seems to cover most things.*  *Reference 3 - 10.52% Coverage*  *R: On I don't like that sort of…I’ve seen things like this before and to me it’s just…I don't find it makes any sense to me anyway.*  *I: Yeah no so my question for you was going to be which survey would you prefer out of the two and why?*  *R: Number one because it’s more definite. This one seems to be you can take your pick.*  *I: Okay so the first one you prefer is this commission tool, so I’ve just because we are going to transcribe this so that’s why I'm just saying this. And the one that you feel is…what did you say it was sorry? It was not…*  *R: It’s just you could be….you could put something down there and it could be taken either way. In too many ways.*  *I: Okay and that’s the considerate tool that you’re talking about there.*  *R: Yeah I just don't find that, well it reminds me of oh I don't know some of these religious things or something. So you know then we can mark you down as you know.*  *I: Okay so you feel it’s putting a label on you as such or…*  *R: …yeah I think that yeah…*  *I: …when you’re talking about when you put yourself down as a specific religion how you follow a religion?*  *R: yeah it could be like that yeah. Yeah I don't know I just don't, I’ve seen things like this before and to me they don't make any sense.*  *Reference 4 - 2.81% Coverage*  *R: …well how to rate your attention to your physical problems like things like pain, dry mouth or trouble breathing. Very bad, good, very good, doesn’t apply. Well…why can’t you write what really you think your problem is? You know sort of I have trouble breathing because I smoked. It doesn’t mention anything like that you know.* |
| **Consistency and simple scale is important** | *Reference 1 - 1.39% Coverage*  *Yeah doll it’s got like how would you rate our attention to your physical problems, things like pain, dry mouth, trouble breathing. And you’ve got very bad, bad, good, very good or doesn’t apply. So that’s the same to all the questions you have that choice of like very bad, bad, good, very yeah. And then you’ve also got, you can write extra on the back.*  *Reference 2 - 3.00% Coverage*  *I: what specifically about the set out? Is it the questions? Is it the formatting…*  *R1: …it’s easy….*  *R: It’s a lot easier. To me that’s a lot easier to…*  *R1: ..that’s quick…*  *R: …to understand and do.*  *R1: ..can I say like a lot of the dialysis patients would easily be able to…*  *R: …to do that.*  *I: Okay*  *R1: …skim through that and do that and be happy to hand that in.*  *R: Yeah*  *R1: I'm only speaking from what I know. But when there’s a section to write an answer, a section to write an answer, a section to write an answer for every question …*  *R: …See this one on here…*  *R1: ….it’s too much.*  *R: …Sandra? Has got always, mostly, sometimes, really, never, didn't apply. This one is much better it’s got very bad, bad, good, very good doesn’t apply.*  *R1: Yeah.*  *Reference 1 - 1.81% Coverage*  *: I don’t think a hospital anywhere would have very bad or even bad. One of those two just bad would do.*  *R2: Every questionnaire that I do that has the scale never or they always go down the middle unless they were just a bit better or horrible or very good and I always it’s just an automatic thing straight down the middle unless they can’t read it.*  *R: We don’t have to read it. Even that the questions there the options are there, the comments in the box underneath.*  *Reference 2 - 0.66% Coverage*  *R: On the other page on the back but it’s just got the questions on the front but tick the boxes very bad, bad, good, very good.*  *R2: It would have to be an extreme for me.*  *Reference 1 - 1.86% Coverage*  *R: I don’t like these?*  *I: So you’re pointing to the Commission Tool?*  *R: Yeah the response options.*  *I: So you don’t like the always, mostly, sometimes, rarely?*  *R: No I don’t.*  *I: What about the very bad, bad, good, very good?*  *R: I find that to the point yes.*  *Reference 1 - 8.11% Coverage*  *R: Umm….I guess I like the second’s formatting. I guess I don't …I guess I'm pretty used to doing like you know (net promoter scores??) I guess so having a very distinct very bad, bad and very good and doesn’t apply, I’d probably change the wording.*  *I: yeah so that’s for the considerate tool?*  *R: yes. Yeah. Umm because I guess you’re just trying to associate a feeling with the care then rather than umm you know like a net promoter score, sort of rating. Some people would probably …it’s very hard to say something is very good or very bad I would say. So I don't know if you would get a good range of responses with that category. Whereas in the other one having always, sometimes, mostly probably covers the same sort of umm response that you’re wanting to get from them I guess. I like the format of this one, there’s a lot of (?) and it’s not as long. I guess and you’re probably getting the same sort of questions out of the carer as well.*  *Reference 1 - 0.40% Coverage*  *R: I think the response thing you know like always, mostly, sometimes like I think that’s an easy one for them to answer quite quickly. You know you’re not going to ponder backwards and forwards as much, someone will make a decision fairly quickly on that, what they felt.*  *Reference 1 - 4.13% Coverage*  *I: Can you maybe elaborate on that, why…what from the survey would you find it easy?*  *R: Well it’s a very plainly put out. And it’s not you know A, B, C, D, E, F, G you know it’s words rather than letters. Yeah.*  *I: Than letters?*  *R: Yeah, yeah*  *I: Yep and is there anything confusing or poorly worded in the survey at all?*  *R: No.*  *I: So you understand all of the questions there’s no like…*  *R: …yeah…*  *I: …health jargon or lingo that you don't quite get?*  *R: Yeah, yeah.*  *Reference 1 - 4.46% Coverage*  *R: Okay my first thing would be when I think of something as being very good that’s my number ten or five whatever it is…and that should be at the end of my row yeah? That should be my last thing because it’s the highest. And then I get doesn’t apply so that confuses me because I naturally go to tick this box and actually I need to tick this box. Which is fine.*  *I: So that’s a formatting sort of…*  *R: So it’s a formatting thing where it doesn’t look user friendly for me. If the doesn’t apply which has to sort of has to float at an end I think I’d go doesn’t apply, very bad, bad, good, very good you know. So doesn’t apply starts over here before very bad and we just shunt everything along. I don't know what these are for but they are very confusing.*  *Reference 1 - 1.76% Coverage*  *: Oh only to sort of…ahh…other than putting in some sort of order…*  *I: So like sort of sub…you’d like sort of sub-headings like…*  *R: Well yeah just they’re all just thrown in there as being of equal importance and I'm not sure that they are.*  *Reference 1 - 1.73% Coverage*  *R: Yeah umm one thing I …the response is opposite. And I yeah it, I think you’d probably get a better (??) and mark, a bad mark on this because it’s on the left hand side.*  *I: On the left hand side so the formatting?*  *R: Yeah so but that’s probably something that you guys would work on but…*  *Reference 2 - 1.09% Coverage*  *R: Umm ease of reading. There’s a lot of information for so many lines and processing that information in a square like this, this is a lot easier. That’s probably the way I’d think.*  *Reference 3 - 1.53% Coverage*  *R: yeah, yeah as soon as I saw that I thought well hold on a second this is going the opposite and I think that, and I think going down is probably a good way too because…*  *I: ..okay…*  *R: …because you know you’re going from first to last by this one does it.*  *Reference 4 - 1.62% Coverage*  *I: So you like on the commission tool having the option for one you prefer the wording of the options that you’re given. But for two you like how it’s formatted going from top to bottom.*  *R: Yeah*  *I: So that you can actually sort of see that a little bit better.*  *R: Yeah*  *Reference 1 - 1.60% Coverage*  *R: You see what bothers me is very bad, bad, good, very good, doesn’t apply. I’d like to then have another line underneath where it said something like write down the three words you can think of that would most apply that you would most apply to this thing. Even if it’s just three words that said that you know said brilliant, wonderful and fabulous you’re in a different area to very good.*  *Reference 1 - 5.69% Coverage*  *R: Yeah. But this one is always sometimes or something it’s a little bit more but it’s fine. It’s, I can answer this yeah at least they don't …always, or mostly or…always, mostly I think this one. They can’t always you know a hundred percent.*  *I: So just because it’s being recorded, so you’re basically saying that the commission tool you don't like how you’ve got the five responses, you prefer the less responses?*  *R: Yes laughing*  *I: And you prefer the bad, good type responses than the always, mostly type responses is that correct?*  *R: But this is important I understand, I can do this just you know if I say mostly I need to give the explanation right. I like always or something like that you know what I mean?*  ***12:28.3***  *I: yeah okay. So you feel like if you were filling out the commission tool the way you’re having to answer you might also have to explain why?*  *R: Yeah, yeah. Yeah, yeah.*  *Reference 1 - 3.80% Coverage*  *I: Did you find anything that was confusing or poorly worded in the survey itself?*  *R: No not at all.*  *I: So there’s no questions that you didn’t really quite understand?*  *R: No not really. You do get very used to going always and then you get to one that you have to read a couple of times because it’s actually a no at the bottom of the question. Yeah which is probably a good thing because it makes people concentrate.*  *Reference 1 - 1.02% Coverage*  *Oh okay so you like the layout of the considerate questions….*  *R: …yeah because this is so easy like very bad, bad, bad, tick, tick, tick you can flow through it.* |
| **Wanting an ‘average’ in ConsideRATE** | *Reference 1 - 3.94% Coverage*  *I: Some people have said that they really like the option of having that sort of prompt. What do you like about that? So what are your thoughts around that?*  *R: I would like that I would like in between good and bad like average.*  *I: Okay.*  *R: Because I don’t like to jump from good to bad you know it doesn’t look good you know. But that’s probably the only thing.* |
| **Language and literacy** | *Reference 1 - 0.65% Coverage*  *R1: The words of some of those could be improved but I think the way it’s framed it suggests an openness or honesty in the response whereas this one even though I like the format of it better I think it’s more readable it does sort of pre-empt.*  *R3: You’re right because even in Question 1 it says “my views and concerns were listed to” so that’s sort of putting words in their mouth to agree.*  *R4: It gives you a positive statement.*  *R1: My mother never wanted to be a problem to anyone. She would have agreed.*  *Reference 2 - 0.54% Coverage*  *R2: If you’re after proper data let’s look at these research finds if you’re after proper data then you’ve got to cater for all the cultural groups. So if we’ve got reading problems, if we’ve got cognitive problems, we’ve got language problems or even I was burnt you might have physical because my hands are bound up.*  *R3: There’s a lot of people that can’t read.*  *R4: Literacy let alone health literacy just normal literacy.*  *Reference 1 - 1.04% Coverage*  *I: There is but you’re wanting to capture their experience.*  *R1: But its retrospective isn’t it?*  *I: No this is real time.*  *R1: Real time but I was involved as much as I wanted. When a need could not be met staff explained why. It’s got a slightly retrospective feel to it.*  *I: Yes.*  *R1: When I was in the hospital I felt confident in the safety of my treatment and care.*  *R2: They must have been in for a few days.*  *R1: It feels a bit retrospective to me.*  *R3: And most of our patient experience is retrospective.*  *I: Yes.*  *R1: So I don’t know how you would get around that but that’s something [name] would have to …*  *I: So you could just change some my individual needs are …*  *R1: Are being met or something if you decide to go with this tool.*  *Reference 1 - 1.68% Coverage*  *R: Again I’d just if I had a tertiary education I’d say if I was a patient with a tertiary education I’d say no but given that I’ve mixed with a lot of very ordinary people I’d say some of the language is too clinical. Like individual needs, views and concerns when a need could not be met is not because sometimes it’s the need is not exactly me asking for tea in the afternoon or even it’s also unexpressed needs.*  *Reference 1 - 5.31% Coverage*  *I: If you were like just by reading through it is there anything that is confusing or poorly worded from the survey?*  *R: No I don’t think so.*  *I: No. You didn’t find any words that you might not have understood the meaning of?*  *R: No.*  *I: Obviously in medical terms I suppose there’s lots of words that can be confusing to people.*  *R: Yeah this is all about just care so it’s fine it’s easy to understand.*  *Reference 1 - 1.81% Coverage*  *R: Let me have a look at this page here. Oh I received the pain relief….they met my needs. Oh I think that’s fine, yeah that’s fine yeah so if I ask they always give to me. But I don't think they need that much but it’s fine, it’s fine the question is fine it’s me. My Chinglish is …*  *Reference 1 - 0.39% Coverage*  *R: No very straight forward and very easy to read.*  *Reference 1 - 3.14% Coverage*  *R: Oh you could do that that’s no problem everyone’s yeah that’s fine you know. That’s a guideline, that’s a good guideline but you can well everyone’s got their own opinion on things you know.*  *I: Is it something that you would use?*  *R: I would if I ever got the time to write it all done. Yeah but I mean to say the way I write things and the way my brain goes around the corner it could be quite twisted but there again it couldn’t be it just depends on what I say and it’s what I do you know. Just what I said just before you know I probably went from down there up to here and then shot out there somewhere and come back again. It is what it is.*  *Reference 1 - 5.27% Coverage*  *I: What do you think about the content and the wording of the questions themselves? Do you find anything either confusing or poorly worded or confronting at all?*  *R: I don’t personally. One of the sorry I’ve got to stop thinking from a professional point of view.*  *I: No, no, no it’s definitely still think like a professional. We want your opinion and your perspective.*  *R: I mean “physical problems” using the word “problems” is always a bit iffy in the sense that it creates potentially the wrong perception of something so I would change that to “how would you rate our attention to your physical concerns” or something like that. And then you can have your example. I would make sure you actually put example before that and say things like “pain, dry mouth” and that sort of stuff.* |
| **Free text is important** | *Reference 1 - 1.31% Coverage*  *I: Oh no so it’s just the last page that free text option where you have the choice to actually add in any additional comments.*  *R: Oh no that’s a great idea that.*  *I: Great idea?*  *R: Yeah, yeah that’s a great idea yeah.*  *I: So that’s something that you would definitely use?*  *R: Yeah oh definitely …*  *I: ..given the choice?*  *R: Yeah*  *Reference 2 - 1.34% Coverage*  *: Okay what about the length of questions? So the considerate tool is seven questions long and the commission tool is twelve questions long. Do you think …*  ***20:24.5***  *R: …I think that’s enough because on the back there you can still write any other extra feelings you’ve got.*  *I: Okay so that’s the considerate tool that you’re talking about….*  *Reference 1 - 6.03% Coverage*  *I: Did you like the option of the free text at the back?*  *R: Yes.*  *I: So that’s actually something that we’ve added to the survey.*  *R: Okay.*  *I: The actual Commission survey doesn’t have that free text option. Would that be something that you would actually use?*  *R: Yes.*  *I: So you like that. We have had suggestions from previous interviews that having the option of having text underneath each of the questions so you could elaborate. What do you like would you prefer that or would you prefer just having the text at the back?*  *R: Just the text at the back.*  *Reference 1 - 1.82% Coverage*  *R: As I said to you the other day I probably wouldn’t write on that personally if I had a brief comment on each of those I’d write it directly under.*  *I: You’d add it in that space underneath the question?*  *R: I’d add it in the box. There always a chance that I might want to go over to the page but just to keep it as a brief comment relative to the question I’d probably write it in that box.*  *I: Okay.*  *R: Why was the box put there if it wasn’t meant to be written into.*  *Reference 2 - 4.45% Coverage*  *R: As I said the other day I prefer that and I still do. It sort of allows me well this one if nothing else you don’t get the option of putting it there or a more lengthy version over the page you have to turn over the page to write something.*  *I: And you’d like that option to be able to if you wanted to to be able to put some brief comment or …*  *R: Well I like that option that’s the question, that’s the answer, that’s it. But this one you’d have to go over the page and number it number one and then do something else and then go back over the page and number two, number three and they’re not there’s a disconnection between question and answer.*  *I: Okay.*  *R: If you prefer to comment. Like if someone says number one is very bad you’ve got to go over the page to write why. I’d much rather that within no space of the question and answer.*  *I: So if we were to use the Considerate questions in our next phase of the study your recommendation would be to definitely add in something?*  *R: Put two lines under it instead of having that with 20 lines forget that break the questions up and four on this page and four on that side and put four lines between.*  *Reference 3 - 0.89% Coverage*  *R2: I never write comments.*  *R: I do sometimes.*  *R2: Unless they’re really standing out about something or really want to make a good comment about something.*  *R: This one has got the room for writing things.*  *I: On the other page.*  *Reference 4 - 0.79% Coverage*  *R: If I felt strongly enough to write a comment I would want to do it in a way that it was clear cut and there’s no way anyone could misinterpret which question was linked to which answer I’d pick this one.*  *Reference 1 - 8.10% Coverage*  *: We added that third page which is the free text option. What do you think of that?*  *R: I think that’s a good idea.*  *I: Is that something that you would use?*  *R: Yeah I would most definitely because there’s been a few things that I have felt a bit in the dark about. Because we transferred from [name of hospital] and I do all his medications. He has a Webster pack so I know what he has and I add a couple of other things like Targin that the chemist prefers I put in and his Burinex which I put the one tablet in every day. And because I’m not here when the tablets are given out I worry that he’s getting his tablets and that wasn’t just here that was at Redcliffe as well. And when I ask questions it’s like I’m an what would you say untrained person if you know like me asking a nurse have you done this makes me feel like I’m being a bit pushy. But I like to be assured that he’s getting his blood pressure tablets and you know and I quite often say have you had your tablets.*  *I: So having that option to be able to write things down is important to you?*  *R: It is.*  *I: Because there’s things that might fall outside of the box.*  *Reference 1 - 6.30% Coverage*  *R: I'm okay with that, I’d like that to be umm I guess in a Word document fine umm…if the questionnaire were in a pdf format where you could select radio buttons next to it that might be easier for you guys to collate the information more quicker as well. I don't know if you have OCR technology that actually you know takes those comments and work out what people are trying to say overall as well. Yeah?*  *I: Yeah*  *R: Because the Word document is pretty manual for someone to fill it out with pen and paper. If it was an editable pdf for the first two pages, free text in that last one and then had some sort of technology for you guys to be able to collate that data. Because there’ll be a general thing in that third page I would say.*  *Reference 1 - 2.64% Coverage*  *R2: For each question yeah I think often you get a survey where it’s you know you choose sometimes or rarely and then you get another little section to provide a comment. That could be useful because by the time you get to here …*  *I: You might have sorry I shouldn’t be leading you sorry.*  *R2: You might not be able to capture.*  *R: Link it all to answering the questions or it might not make that much sense to a researcher.*  *R2: But the qualitative data’s often really useful.*  *Reference 2 - 2.11% Coverage*  *R: Anyway I think this one’s much more user friendly.*  *I: So that’s the considerate you’re talking about?*  *R: Yeah the considerate.*  *I: Sorry I’m just because of the tape recorder sorry.*  *R: But it again if you could link you know comments the additional things you wanted to share to questions or suggest that people link them to the question if they wanted to that would be good.*  *Reference 3 - 2.07% Coverage*  *R: Which is one of the problems. But it makes the questions really important to doesn’t it because if you’re not asking the right questions well you’re not necessarily dealing with you know problems don’t come through the survey you know.*  *R2: Yeah it’s where the qualitive data does come in really handy for improvement. I don’t know whether we’ve been very helpful.*  *Reference 1 - 0.24% Coverage*  *R2: you know could be instead of a text box in the three having just the notation to say please elaborate on comment section, could be the easiest way to do it.*  *Reference 2 - 0.43% Coverage*  *R2: Yeah but if they’re unhappy with something usually they would be happy to go to the comment section. So it could be as easy as doing that because that could indicate the clinician. Or it could indicate for the concern and we’re smart enough to figure out whose responsibility that was.*  *Reference 3 - 0.59% Coverage*  *R: I think you definitely need that. Because there will be some who really need to explain that experience. Because you know like the questions here won’t delve to some of these things that are not going to be listed here. There’s going to be other things that they’re going to want to, not everyone…*  *R1: …or they’ll write on here, I think if we didn't have it.*  *R: Yeah they will, they will yeah.*  *Reference 4 - 1.86% Coverage*  *R1: Yeah I think that would be great yeah.*  *R4: I think you can just put one in on the back couldn't you? It says write anything here I think instead of under each of them you could write at the top where you’ve got the who is that, what is the score and you could have just a line under saying ‘please elaborate within’…*  *R: ….elaborate on the back.*  *R4: …on question eight if you feel it necessary’.*  *R1: I personally like the text box under each question just so they have that visual prompt to elaborate on the question rather than getting to the end of the questionnaire…*  *R: …I think they’re more likely to say “Oh it was so and so that really…” then they could elaborate on that more. But if it’s just something small they could just put it in and move to the next question.*  *R1: Yeah it might be as simple as like you know how would you rate our attention to your surroundings? Noise, light, warmth? A little text box under that would then maybe…’it’s really noisy in the hospital the lights never go off’ or whatever it is.*  *R: …the blue curtains are rubbish.*  *R1: Yeah. You get that straightaway. Whereas by the time they get to the bottom of the questionnaire I don't know I'd that’s going to be the focus of what feedback they want to give.*  *Reference 5 - 1.08% Coverage*  *R4: I feel like if you have the text box you don't need this one.*  *I: Okay.*  *R: Particularly if you want to try and shorten it to keep it short and sweet.*  *R2: yeah if you want to keep it within like you could do this on two pages. If you had a text box under each one and then….but if you had a text box as well as this it would be making it up to the commission’s size where you’ve got three pages.*  *R: You will always get that one or two people who just need to say a lot more.*  *R2: I think give them additional pieces of paper.*  *R: You can always give them…*  *R2: …they bring more out…*  *R: ….I think an optional…lots of laughter and chatter….if you’ve got more to say please…*  *R2: …please ask …*  *R: Please ask for extension paper.*  *Reference 1 - 0.83% Coverage*  *R1: Yes I do. I always fill them up. The form that I send back to the hospital …*  *R4: I think people just want to complete but some people wouldn’t use them.*  *R3: I think it’s good again it gets back to communication like I don’t think a patient should feel they have to write on here if they don’t want to but they may not know that if they’re just given this form they might feel pressured to fill the space.*  *R4: Sometimes it’s daunting as you say a whole page. If I write something I can’t just write one sentence.*  *R3: But if they’ve got someone sitting there with them explaining you can write one sentence or as much as you like or nothing.*  *Reference 1 - 1.82% Coverage*  *R: Well questions with multiple choice are always easier when writing something. Laughter*  *I: Yeah sure do you feel you want to provide specific additional information or not so much?*  *R: No not so much.*  *Reference 1 - 0.96% Coverage*  *R: Oh that’s a good idea. So if someone does have a thought well they can jot it down and it can be considered.*  *Reference 1 - 5.68% Coverage*  *R: Honestly I look at it and go “Oh my God no, no I don't want to write an essay”*  *I: Too many lines.*  *R: yeah.*  ***9:18.3***  *I: Yeah no problems so it’s something that you possibly wouldn't use?*  *R: It puts me off even before I start reading the question.*  *I: Okay just looking at all those lines?*  *R: But I appreciate some people want to elaborate but I think the fact that there are so many lines means I think I have…*  *I: …you have to fill it?*  *R: ..I have to fill it in. Which I know is stupid. And…*  *I: No don’t be sorry. We have conducted a number of user interviews already and from previous interviews people have actually sort of suggested having a couple of lines sort of underneath the questions themselves. What would you think of that as an option instead of that big, since you don't like having that whole page of lines is that something that you would…*  *R: ..it would probably …*  *I: ..probably…*  *R: .. be something that’s easy to face.*  *I: Okay yeah*  *Reference 2 - 2.50% Coverage*  *R: And you know the other bit you can complete if you want to but you don't have to. But it’s a lot less …it feels like a commitment. When you came to ask me if I could help my instinct is I want to help because the system has helped me so much. But equally I don't want to be filling out lots of forms and having to speak to somebody …*  *I: …forever…*  *R: …forever and you know. So this makes me feel like the forever part.*  *Reference 3 - 3.66% Coverage*  *R: Can’t do it too short then you’re not going to get the depth of knowledge that you need. And I think most of us should be able to cope with twelve questions. Especially if you got rid of this and put them under here.*  *I: Yeah. So that is getting rid of the free text on the third page of the commission tool and putting some sort of text box underneath the questions themselves.*  *R: Which the only thing that does do is take away the ability to be general. If there’s something somebody wants to say that doesn’t relate to your…*  *I: …to those questions…*  *R: …questions. So maybe you still need one free floating box.*  *Reference 1 - 1.34% Coverage*  *R: Yes, yes I’d use that.*  *I: Yeah.*  *R: Usually this section is not long enough for what I want to write but that one might be.*  *I: That one would be long enough?*  *R: Yeah I think so.*  *Reference 1 - 0.77% Coverage*  *R: I couldn't write no.*  *I: Yeah because of like you are limited…*  *R: ..the only way I can do it is yeah, yeah. It’s a bit hard to write at times love.*  *Reference 1 - 3.07% Coverage*  *I: So we added the free text option at the back of the survey that actually wasn’t there. What do you think about that?*  *R: This?*  *I: Yep.*  *R: No I think that’s a good idea.*  *I: Is that something that you would use yourself if you had the choice?*  *R: Probably if I could sit and think.*  *Reference 2 - 2.65% Coverage*  *I: Do you think that’s something you might not use you prefer having that bulk text free answers at the back?*  *R: No I reckon it’s a good idea because otherwise you don’t know what’s happening do you? You’re saying never and you don’t know why.*  *Reference 3 - 2.85% Coverage*  *I: Okay. We’ve also been so very similar to the Commission Tool were having the option of having free text underneath the questions what you think about that?*  *R: That’s a good idea to because some people would rather put down in their own words what something is.*  *Reference 1 - 1.23% Coverage*  *I think it’s good to have the option because some people are much more fluent with their writing than they are with these things overall. So I think it’s a …yeah I reckon it’s fair enough.*  *Reference 2 - 0.84% Coverage*  *R: I most likely wouldn't but only because I have problems writing…*  *I: …because of your writing…*  *R: …and stuff like that. Yeah.*  *Reference 3 - 0.46% Coverage*  *R: Oh I'm not a wordy sort of a person so I most likely wouldn't no.*  *Reference 1 - 0.63% Coverage*  *R: I think that’s excellent.*  *I: And you would use that?*  *R: Sorry?*  *I: And you would use that as an option?*  *R: Oh I would use that right to the very brim.*  *Reference 1 - 2.93% Coverage*  *R: Well I think it gives people the option to make comments and add stuff that isn’t part of that isn’t the answer to a question.*  *I: Is it something that you would potentially use?*  *R: Yeah.*  *I: Okay.*  *R: I’m a chatterbox.*  *Reference 1 - 0.20% Coverage*  *Oh definitely. Oh yes.*  *Reference 1 - 4.08% Coverage*  *We added that free text option at the back of the survey.*  *R: I saw that.*  *I: What do you think about that?*  *R: That’s good.*  *I: Is that something that you would use or it’s something like I’m not really one to put extra?*  *R: In my experience at the moment if I was filling this out right now I would actually put a comment in there and the comment would be positive and it would be that I was very impressed with the way departments have worked together.*  *Reference 1 - 3.14% Coverage*  *R: Oh you could do that that’s no problem everyone’s yeah that’s fine you know. That’s a guideline, that’s a good guideline but you can well everyone’s got their own opinion on things you know.*  *I: Is it something that you would use?*  *R: I would if I ever got the time to write it all done. Yeah but I mean to say the way I write things and the way my brain goes around the corner it could be quite twisted but there again it couldn’t be it just depends on what I say and it’s what I do you know. Just what I said just before you know I probably went from down there up to here and then shot out there somewhere and come back again. It is what it is.*  *Reference 2 - 0.73% Coverage*  *R2: It’s good to have the spaces here where you can write your own stuff where with this one there’s nowhere to write anything you just have to pick one.*  *Reference 1 - 4.80% Coverage*  *R: Yeah I think a lot of that depends on what you want to get out of the survey but it’s a hard one because if you ask I’m always very wary of how you include the open comment stuff and whether it should be attached to individual components or whether it’s just a broad reaching one. Because part of the issue about putting broad reaching one is that you can have it end up being in a situation where you’re receiving information in the study that it could be somebody who’s actually writing down and complaining about something and that sort of stuff. Which if you’re receiving that information could be problematic from their perspective but I think there’s always you always need to have some space for some people to …*  *Reference 1 - 0.71% Coverage*  *: If I had time. I’d sit down and think about it and write a few things out to help you to help them to help me.*  *Reference 1 - 0.52% Coverage*  *R: Yeah alright if somebody has got something to say they can.* |
| **Frequency for PREM completion** | *Reference 1 - 1.48% Coverage*  *R: I reckon every three months, every twelve weeks I reckon.*  *R1: and if you’re an in-patient and you get changed to a different ward, I think it should be…*  *R: …each ward…*  *R1: ..each ward you go to. Because different wards honestly…*  *R: …run differently…*  *R1: …wow. I was on level 8 was it 8?*  *R: Yeah 8 love yeah.*  *R1: And it’s run totally different to this ward.*  *I: Okay.*  *Reference 1 - 3.79% Coverage*  *R: Close to the beginning, the middle and then like just before they leave.*  *I: So you think we should give it a couple of times throughout the duration of their hospital stay?*  *R: Yeah.*  *I: And for every hospital stay or once every six months?*  *R: Just the ones that are chronically ill.*  *I: Okay.*  *R: It gives them a good idea of where they’re at as well.*  *Reference 1 - 0.32% Coverage*  *Probably a day or so before they leave maybe or depending on how long they’re here.*  *Reference 2 - 1.08% Coverage*  *If they’re here for six months maybe a couple of times in that time but after a day or two you can’t get an idea of anything because you might have just hit a ward when it’s run off it’s feet and things are a little bit chaotic. You’ve got to let them settle and then go from there.*  *Reference 1 - 4.53% Coverage*  *R: Umm…I would. I think it would be interesting to see a third party that could you know just provide an overall rating. Because it needs to be taken not just once I guess you know so that there’s different parts of when you are an in-patient. There’s when you’re admitted, so when you’re admitted the answers might be different to when you’re moved to a ward and being treated to when you’re discharged. So if it was to be done to an in-patient the timing of the in patient’s road map would need to be considered in that as well.*  *Reference 2 - 8.68% Coverage*  *R: Well I guess yeah I guess it’s the key points of the point in time of the patient’s care you know. I think this time nan might have been in Emergency for ten hours. So you know if they’re in Emergency in that time waiting to see if they’re even going to be admitted would you want someone to fill it out then if they’re not then going to be an in-patient? Maybe not. But maybe in that first time once they are admitted maybe a question around that process of your admission would be interesting to see. Communication between those different areas of the hospital while it might be there in the back you know the patient is not necessarily aware of that stuff either. Nor should they all the time. Yep. As long as it doesn’t impact on their care so probably you know when they’re first admitted to when they are released you’d probably want to see the change in the survey in that time. You know they’re saying it’s very bad the whole time for one particular area then at least you’ve got something to work on then.*  *Reference 1 - 9.22% Coverage*  *R: So it’s a good question because I assumed it would be once during their stay but obviously people you know what I was getting at before talking about people coming in and out of hospital they develop a body of knowledge actually about what’s good and what isn’t. You know if you go to hospital once like I wouldn’t know I haven’t really spent much time in a hospital if I went into hospital I wouldn’t know what was really good or not. But if I’d been in three times or been in there for a long stay you’d develop that knowledge. So in some ways it’s a bit hard to get them to compare their experiences but in some ways if you could capture some of that somehow it would be good. So doing it more than once might do that. So that’s a very long way of not answering your question.*  *I: No that was helpful like a different way of seeing things because I would never have thought about it like that myself.*  *R2: So some patients I mean hospitals like their patients to stay the minimalist time possible so that the bed’s vacant for the next sick person coming along. So some people could only be in for say two days and others like [name]’s Mum this is her coming on for her third week. And oncology patients would be perhaps coming and going fairly frequently throughout their illness.*  *I: Yeah.*  *R: So I would think maybe every couple of weeks. It’s kind of hard to know. Yeah I don’t really have a great sense of why you would do.*  *I: Just capturing different cohorts’ opinions what would that do?*  *R: I mean for an individual.*  *I: Oh yeah okay.*  *R: Because every patient I presume would fill one of these out. Is that how it would work the survey?*  *Reference 2 - 0.62% Coverage*  *I: So do you think maybe the timing of when surveys are given is important?*  *R3: I think it would be paramount.*  *Reference 1 - 1.27% Coverage*  *R: Not all the time as I said like probably maybe once every three months or something.*  *I: Okay.*  *18:46:2*  *R: Once every three months just to get to see how everything’s progressing and so like it gives me something to do as well to.*  *Reference 1 - 0.21% Coverage*  *R: At the moment I’d say about every three months.*  *Reference 1 - 3.37% Coverage*  *R4: It’s going to vary seasonally as well based on patient numbers and hospital bed pressures and other things. Like I think if you gave this questionnaire set out to a Covid patient in a Covid ward I think you know they’d probably you know for a lot of them they’re sitting there by themselves, no contact with people and they’re probably report low. If the hospital is under a lot of pressure, people sitting in ED for a long time it’s going to be lower. So I don't know if doing it really frequently would help but I think you’d have seasonal sort of changes based on what’s happening over the rest of the hospital. If it’s winter and the hospital is full and…*  *R: …certainly random seasonally changes like…*  *R1: I was going to say, yeah because I was going to say if it’s too infrequent and it’s something people plan for and it’s expected it’s like accreditation then you know and I don't think that…it’s helpful at all.*  *R: Yeah it certainly can’t be too frequent because then it’s yeah.*  *R1: But yeah I think that not too often but definitely not like something that’s done twice a year that you know everyone is aware of and preparing for. Because I think that will skew sort of…*  *R2: But like are we be missing patients though if it’s like are we supposed to be catching more patients that are on the palliative way? Or is this just for anyone?*  *I: Look I think that …*  *R2: …just thinking about who if we did it say once a month, if there is nobody who would fit the criteria of what you’re looking for on the day that we do it then we’re just not going to gather any data. So I do love the idea that the Q R code and something easy that you could actually just give out and they do. And then maybe a specific day to try again with people who couldn't access the Q R code or…I would use paper on my ward. Because I just would give it to people and tell them to do it.*  *R1: Mmm you can aim for a certain number each month. So with the patient experience they will have ten a month. And each week we’ll try and do at least two and you obviously figure out who you’ve asked and have your list and cross them off. So if you I guess if you have a number in mind and if it’s like everyone is happy with that number you could just aim for that number over a month yeah.*  *Reference 2 - 0.23% Coverage*  *R: …I think ideally it would be you know it’s not going to happen. But the population of the hospital at that time. But not everyone is going to do it.*  *Reference 3 - 2.68% Coverage*  *R: But I think ideally it would be nice to know what everyone was, what their opinions were at that time on that day on the umm you know. Clearly not every single person is going to do it or want to do it or be capable of doing it. But you know I think aim as high as possible for that particular time.*  *I: yeah and…*  *R: ..and your resources can be put to that time, specifically to be done and then…*  *R1: ..yeah that’s right just another thing that has to…*  *R: …it’s not a drag down ongoing…*  *R1: …on the list to …yeah…*  *R: …yeah.*  *R1: Yeah I agree yeah.*  *I: And done within the ward resourcing or you would need external people to do it?*  *R1: to do a like big volume of people like a large number of the in-patients I think we’d need assistance umm but…*  *R: …I think assistance but you could always get that from the wards. I'm sure you would find people on the wards who would put their hand up and say “I’d really like to be a part of that and do that as a one day, two day…” whatever specific. But I think external could help because sometimes the wards are just manic. People don't have the time to give the patient time to explain it, go through it yeah.*  *R1: And we don't have indirect resource in general medicine. I can speak on behalf of the service line probably so like I don't have spare people that can give up half a day or this or that to be able to do something like this. Like the staff that I have run on the floor every day. So even that feeling then is you know yeah so it would be, if it’s something that is put back onto the ward it would have to come second to all of their you know clinical tasks essentially. Which could be problematic on a code yellow or whatever it might be you know. But yeah there’s no one I can pull from a role to assist unfortunately we just don't have it.*  *Reference 4 - 0.66% Coverage*  *R2: Mmm that’s why I was saying what’s the prognosis that we’re looking at to target. Yeah so with that one I agree that it would be good to have somebody externally to be able to do that. I could probably find staff that would be like eager who are wanting to like act up into the different roles and I could get them to do some of the additional surveys. But I couldn't guarantee a high number per month dependent on our acuity level at the time.*  *Reference 5 - 0.17% Coverage*  *I liked the idea of maybe doing it on discharge as well because you couldn't give it too early in the admission.*  *Reference 6 - 0.84% Coverage*  *R2: Would you not do it in an outpatient setting? Like is it not, I think that if you were trying to capture people like twelve months prognosis and especially if you have a patient waiting in an out-patient like they’re sitting in a waiting room and they see a sign. I feel like maybe you’d get a bit more like more responses.*  *R1: Coming to a clinic or something.*  *R2: Yeah so they’d be discharged but they know that they have this twelve month, six month prognosis and they might be seeing the palliative care team or something like that. You may get a bit more…*  *Reference 1 - 0.31% Coverage*  *R3: But I’m looking at it from a very different perspective possibly from a patient who’s somewhat stabilised and has some space to answer some of this. Because often patients don’t have the energy to respond to surveys when they’re in hospital.*  *Reference 2 - 1.14% Coverage*  *R3: In order to drive change I actually think it’s good for the medical profession to know directly the answers to these questions.*  *I: Yes. So the Commission Tool is helpful. I keep saying the tool so that our transcript when I go back I can keep highlighting. But the Commission Tool are you thinking [name] would be better after like someone’s stable or discharged home potentially?*  *R3: Yeah I do when they’ve had time to reflect on their experience.*  *R2: That’s how prems are done in this hospital it’s a certain time period after you’ve got home.*  *R3: You need time you can’t answer anything …*  *R2: To get your brain back.*  *R4: Yes that’s right.*  *R3: You can’t answer any of this when you’re traumatised, dealing with sickness, getting your head around …*  *R4: Sometimes close to discharge you can answer some of that because a lot of this to me is …*  *R2: Depends where you are medically.*  *Reference 3 - 0.20% Coverage*  *R5: I mean cognitive capacity would be an important consideration before you even …*  *R3: That was why I was saying after reflection you’ve had time to stabilise.*  *Reference 4 - 0.32% Coverage*  *R1: The issue I have with just handing these out is I think patients need to be prepared. Communicated in some way about what these surveys mean because I don’t think my mother would have done this properly and she hadn’t lost capacity or anything.*  *Reference 5 - 0.39% Coverage*  *R5: Well you know you still have to be a time when the patient has some cognitive capacity you know and has a conversation with who it might be is it a patient advocate or it might be a carer or whatever it is you know. It’s like a patient advocate if we could afford those sorts of things would be ideal.*  *Reference 6 - 1.66% Coverage*  *R3: I think you should do it when there’s some understanding I guess that the patient has stabilised enough physically and mentally to be able to engage with this.*  *I: Yes.*  *R3: And that assessment I guess can only be done between the patient and the staff I suppose.*  *R4: But if you had to give a generalisation on say once discharge is being discussed your discharge from the hospital is being discussed it means that your medical condition is stabilised to a certain extent. Maybe emotionally you might not be still there.*  *R3: Some patients might want to do it before they’re discharged.*  ***47:29:1***  *R4: I’m saying prior to discharge. Like someone doesn’t come to your bed and say you’re discharged out the door they talk to you about discharge the day or two days or they say we’re looking to discharge you in the next couple of days. And some people’s discharge is a bit longer because they’ve got a bit more paperwork and a bit more drugs to do but once the discussion of being discharged that’s when I think it is. Because physically you’re not being discharged if you’re still really, really sick and you can’t look after you. You’ve not being you know emotionally you might still be suffering but that to me if you had to have a generalisation once the discussion around discharge is happening.*  *Reference 7 - 0.97% Coverage*  *R1: I think you need to do it in the ward as well not just at discharge.*  *R4: Well discharge is that extended I don’t see discharge as 8 o’clock you’re gone, 10 o’clock you’re gone. I see discharge as a process because they start talking to you about it discharge it’s a process someone doesn’t just say you’re discharged today and you’re gone there’s a process that you’ve got to go through in terms of getting out. Getting someone to come and pick you up, ordering the transport ambulance or the doctor giving the final clear or you know we have one more blood test and if that comes back good. It doesn’t happen …*  *R1: You don’t only want these things at the end of the person’s stay because some of these things can be addressed while they’re in here.*  *Reference 8 - 0.48% Coverage*  *R4: But you can’t be surveying people all the time and that’s where communication comes in. If I didn’t get what I wanted I should have felt comfortable asking for it. And I can tell you when my Mum was in hospital we asked for these things and we just hit brick walls or the biggest one was I’m not usually on this ward sorry I can’t help you. I don’t work on this ward …*  *Reference 1 - 1.45% Coverage*  *R3: So at the moment and I think these are mainly very similar these are questions that get asked from a patient’s perspective which are done post hospital and there is a series of questions that we ask in hospital so we do as part of our clinical weekly audit. When we last reviewed that there was some discussion especially with the consumer partner with consumer’s needs I guess in Metro North. Just some discussions around what kind of questions we ask in hospital and outside hospital so that we kind of changed our questions slightly in hospital and took some away that were better asked outside of hospital. So there is I guess there’s some questions that are can be asked at either so there were some where I said well I think it’s important for us to know how you’re feeling about being involved in decisions about treatment and care. That is something that you can ask while you’re in hospital because it’s a day-to-day thing and you could also look back on it and say how did it feel. So there are some that work either way.*  *Reference 2 - 1.55% Coverage*  *R3: I’m sort of like time how would you rate our attention to what you know what you can expect things like illness getting worse or time left to live. I can see some of those nurses at the end of the bed going and somebody saying well the doctors haven’t told me how much time I’ve got left to live.*  *R1: And they’re not going to either [name].*  *R3: No I know. But I do have very inexperienced nurses they’re graduates and that would be I think difficult for them. I’m not sure what the group are saying.*  *I: Well the Nums from your wards yesterday actually it wasn’t [name] but [name] was there and [name]?*  *I: [name]. They were concerned about 6 and 7 being administered without someone to support the patient if that did trigger something for them.*  *R3: Yes I’d feel the same.*  *I: Because if they don’t know and then you give it out and then you’re right like they’re asking questions of the team and the team are busy running around everywhere and no one’s coming back to the patient immediately that’s a concern I guess.*  *R1: It’s very hard isn’t it because you know this gets to the core and the heart*  *Reference 3 - 0.44% Coverage*  *R1: That’s the thing you asked us what we really like everyone in here as gone Considerate and then we’re starting to think about the practicalities of delivering a survey or carrying out a survey with someone and not providing the support or response to it. I don’t know what are we trying to do that’s the thing?*  *Reference 4 - 0.67% Coverage*  *R3: I think it also needs part of the implementation of course having someone like [name] would be fantastic but is going to need to be the discussions with the team with the full team because it’s about the full team’s response. So even if the nurses are there when it’s being administered and maybe supporting the patient if they need to when it’s being administered it’s a whole team response is what we’re seeing from these questions. So we need to have everyone on board.*  *Reference 5 - 0.58% Coverage*  *I: I think the real pearl and I think it’s come through today as well from yesterday was distinguishing this between a QI process or a clinical provision process and the clinicians really felt Considerate would inform care and therefore needed a process that would immediately feed back to the team.*  *R1: Where if there was a mechanism in that that would be wonderful.*  *R3: And that was my concern that that’s not there.*  *Reference 6 - 0.40% Coverage*  *: I guess it sort of depends on what you’re trying to achieve. I guess if people have come out of hospital and then we’re probably okay in asking in general people are probably going to be okay about asking about some of the things and not so good at asking about some of the other ones.*  *Reference 1 - 3.73% Coverage*  *R: Well I’d like to see everybody that has been termed terminal you know like motor neurone disease things like that all the really nasty things. They get when things settle down they get it you know.*  *I: So when you talk about when things settle down like maybe their symptoms….*  *R: ..yeah when they know that for sure and they’ve had time to take it in themselves. And then this can be offered to them as a tool. Another tool.*  *Reference 1 - 1.35% Coverage*  *I: How often do you think this survey should be filled out for in-patients?*  *R: Umm…*  *I: Like how often would you like to see this survey?*  *R: Probably each time I was in hospital.*  *Reference 1 - 5.18% Coverage*  *R: Oh no more than once per visit.*  *I: Okay.*  *R: Depends on how long they’re in for obviously and how often they come in. But you know if they’re only in for one day and they answer, they were in for a week last time and they answered all your questions I wouldn't be asking them. I think there has to be a degree of discretion and smarts. Going into how often you give them the survey because at the moment we’re in a survey culture. Which is not helpful for people like you when I literally you know I phone Vodaphone and I’ve only just put down the phone. And they said “Please answer three questions survey”. I said “Well I don't know if we’ve actually done anything yet how can I answer your survey”? And it’s just it’s irritating. Please tell us about your experience in Myer this weekend? No, go away. You’re a shop I bought you did your job. So you need to be careful.*  *Reference 2 - 0.64% Coverage*  *R: No as I said just use discretion as to how and when you give them out so people don't get over-surveyed.*  *Reference 1 - 5.74% Coverage*  *R: Well my reaction immediately would be “Hey I'm in pain I'm not going to be bothered filling out a survey”.*  *I: Yeah so to you it’s very important when someone approaches you with something like this the timing of it?*  *R: Yes I didn't come into hospital to fill out paperwork.*  *I: So with regards to that would you …if someone asked you to fill it out would you be like I’d prefer to do it at home…*  *R: …certainly…*  *I: …certainly. So that’s something more that if it was sent out to you after you were an in-patient….*  *R: …well I’d be more likely to give it more thought. Yeah because like I'm in hospital for a reason and the reason is not to fill out survey forms.*  *I: Yep so the timing is very important to you.*  *R: Exactly I don't want to fill it out in hospital to be quite honest.*  *Reference 2 - 1.99% Coverage*  *R: Oh everyone is different. I mean I don't know I suppose the more feedback organisations get the better so that means more input from more people. So I suppose you know to get to the logical conclusion there yes, you know people probably should be providing more feedback.*  *Reference 1 - 1.87% Coverage*  *R: Yeah about once every six months.*  *I: Once every six months?*  *R: Every month is too much love. Six months and people would read them. But if you’re getting them every month let’s be honest you’re just chucking them aside. And it’s a waste of your time.*  *I: yeah and you believe we should be giving them to in-patients?*  ***20:59.4***  *R: Yeah.*  *I: Okay*  *R: Yeah I do.*  *Reference 1 - 5.29% Coverage*  *I: And how often would you recommend to be giving the survey out?*  *R: Depends on how long people are in for.*  *I: So do you believe every person should be getting a survey?*  *R: If you’re only in for a day or two you’re not going to know the answers to a lot of those questions. So I think more on long term ones I think.*  *I: And with every one of their admissions?*  *R: No I wouldn’t say everyone because some people come in and go home the next day you know. I don’t know the answer to that.*  *Reference 1 - 2.00% Coverage*  *: I think honestly I think it’s probably after discharge on like on visits to your in-patients especially that’s on chronic care are coming in here on a regular basis even if it’s only if they come in every six months. The problem with surveys is you do it more often they’ll start thinking it’s just a toy and it will be trivialised.*  *Reference 1 - 1.20% Coverage*  *R: I think you should be giving I don’t know how often but I know I would welcome this as a way to say thank you. But how often I don’t know I think it would be perfectly possible to keep enough of a record of who got them so they didn’t get it the second time if they were still here sort of thing.*  *Reference 1 - 2.73% Coverage*  *I: And if do you think we should be asking inpatients to complete surveys like these?*  *R: Yeah.*  *I: How often would you think we should ask someone to complete it?*  *R: Oh just once maybe just once on discharge.*  *Reference 1 - 3.60% Coverage*  *R: Oh definitely. Oh yes.*  *I: So that something there at the back survey that you don’t mind having there or you think oh it’s good having the option in case I wanted to use it?*  *R: Oh yeah I think it is good to have options oh definitely not everybody has a say in and the same opinion yeah so I think we should all be able to write down our own experiences and whatever.*  ***07:43:3***  *I: Okay. But you necessarily wouldn’t always use that?*  *R: Probably not.*  *Reference 2 - 1.57% Coverage*  *R: I’d say at least once a year. I think once a year would be enough.*  *I: Okay. And why once a year?*  *R: I think otherwise the questions would be too much similar to what they’ve answered before.*  *Reference 3 - 1.34% Coverage*  *R: Some people would probably say you know six months or something but no that’s too early.*  *I: Okay.*  *R: I think if you know it was yearly you would know it was coming.*  *Reference 1 - 2.14% Coverage*  *If it helps you. If it helps you with your stats and demographics I think it’s probably a good idea. Just that someone who’s come into the system might have less expertise on the system than someone who’s been in it for a month or two.*  *Reference 2 - 2.71% Coverage*  *R: I would have a short survey about that length and I would do it on a regular basis. I would do it every maybe two or three weeks and just see if there are any changes at any stage.*  *I: Okay.*  *R: And then at least you could identify you know where the change was and perhaps identify an issue there.*  *Reference 1 - 1.44% Coverage*  *R: Well I mean to say you can’t go out to the inpatient and say are you well? Are you good, are you fine, can you do this, are you sure you can do this? You know you’re not going to hit them with that. I don’t think so it’s just that if they accept you they accept you if they don’t they don’t.*  *Reference 2 - 2.11% Coverage*  *R: Well that’s a toss of the coin really because you’re just going to take a stab in the dark ourselves.*  *R2: Here we’re putting you in hospital but you have to do this survey.*  *R: That’s right.*  *R2: They’re going to say bugger off. It should be random maybe.*  *R: I think it might be a random thing I suppose it could be too that’s a point.*  *R2: Surely you wouldn’t do it every time.*  *R: If it was compulsory it would be mayhem very much so.*  *Reference 1 - 10.56% Coverage*  *R: That’s a tricky question in the sense that a lot of that will depend on the particular circumstances of that inpatient and the timing of when it actually occurs during their stay. I mean for me like I’ve been here two weeks and I mean I could answer this relatively easily and comfortably but somebody who’s only been in for like two days may have a very different view because they’re not understanding how everything works. It’s almost like have you been in long enough to understand how the institution works. So for me I have a relatively good knowledge of how the health system works and all of that sort of stuff and what doctors and nurses are required to provide to you and things like that. But the majority of people in the community don’t.*  ***21:38:3***  *I: So it’s finding that right time in that patient’s admission to then say hey would you like to complete this for us.*  *R: And from a practical point of view I mean if you gave this to a patient who had been particularly upset about something that’s just occurred you’re going to get a very different response to one that you know has had some time to take in how the system works and works with you.*  *I: How often do you think that we should actually be like how often would you like to see a survey actually given out?*  *R: In terms of the stay in hospital?*  *I: Yeah so should we be doing it every three months or with every admission that someone comes in, or once a year?*  *R: No you only need a sample. I wouldn’t be going that’s a hell of a lot of data if you’re doing it with every admission. No you only need sampling.*  *Reference 1 - 2.73% Coverage*  *Not when you first come in because you’re all emotionally upset. The ambulance and the lights and it’s all pretty dazzling, where are they taking me? You’re going down a corridor laughing like off your face. I wouldn't give it straight away no way. And I wouldn't do it nowhere near the end because you’re excited to go home. You’ve got to get somehow where people are more relaxed and more of a positive feel. And then bang them with this.*  *Reference 1 - 0.82% Coverage*  *R: Oh every six months.*  *I: Every six months?*  *R: Easily yeah.*  *Reference 1 - 0.52% Coverage*  *R: Once every six months or every twelve months I don't know.* |
| **Give patients sufficient time to complete this** | *Reference 1 - 2.77% Coverage*  *I: So you’d need a bit of time if you were to do the survey?*  ***04:07:8***  *R: Yes.*  *I: If someone was to give you that survey it would be like if you can come back the next day type thing and not put you on the spot like I need it in five minutes time?*  *R: Yes.* |
| **Happy to complete a PREM** | *Reference 1 - 2.87% Coverage*  *So if I was to come into you and [name]’s room today for instance and say oh hi [name], hi [name] I was just wondering if you might be able to participate in a survey what would your reaction to that be?*  *R: I’d say yes.*  *I: You’d be more than happy to?*  *R: Definitely.*  *Reference 2 - 1.44% Coverage*  *I: Okay. And if we were to give this for one do you think we should be giving this survey to inpatients?*  *R: Yes I think it’s great.*  *Reference 1 - 0.40% Coverage*  *: But you’re in agreeance that we should be doing surveys for inpatients?*  *R: I think in any business.*  *Reference 1 - 0.80% Coverage*  *I: You’d be happy to complete the survey?*  *R: Of course.*  *I: You’d have no reservations at all?*  *R: No not at all.*  *Reference 2 - 0.57% Coverage*  *: I wouldn’t think that it would hurt anybody to be asked to complete a survey.*  *Reference 3 - 1.40% Coverage*  *R: I think every time.*  *I: Every time.*  *R: Well you know unless they’re here every week they won’t want to fill it out every week but you know it’s just it keeps everybody on their toes doesn’t it.*  *Reference 1 - 0.17% Coverage*  *R: Yeah happy to do.*  *Reference 2 - 4.53% Coverage*  *R: Umm…I would. I think it would be interesting to see a third party that could you know just provide an overall rating. Because it needs to be taken not just once I guess you know so that there’s different parts of when you are an in-patient. There’s when you’re admitted, so when you’re admitted the answers might be different to when you’re moved to a ward and being treated to when you’re discharged. So if it was to be done to an in-patient the timing of the in patient’s road map would need to be considered in that as well.*  *Reference 1 - 0.56% Coverage*  *I: and so as, and then we’ll let you go. As nurse leaders in your various wards and your specialist palliative care team how, do you feel it would be important to implement some sort of experience measure for your patients generally? Chorus of yes, absolutely, definitely. Yeah? Okay. And it sounds like you’re doing it in oncology already but are you doing it in general med?*  *Reference 2 - 0.12% Coverage*  *R1: I think it’s great and like really excited to be a part of it so thank you.*  *Reference 1 - 0.79% Coverage*  *R2: If you’re after proper data let’s look at these research finds if you’re after proper data then you’ve got to cater for all the cultural groups. So if we’ve got reading problems, if we’ve got cognitive problems, we’ve got language problems or even I was burnt you might have physical because my hands are bound up.*  *R3: There’s a lot of people that can’t read.*  *R4: Literacy let alone health literacy just normal literacy.*  *R2: So it’s hard to get that data right because otherwise you’re sampling that one little group all the time or the group that bothered to send it back.*  *R3: You’re just going to get token feedback.*  *Reference 2 - 0.17% Coverage*  *R3: Particularly for someone who is seriously ill they’ve barely got enough energy to keep themselves alive let alone respond to this.*  *Reference 1 - 1.46% Coverage*  *R3: It kind of depends a little bit on who you’re giving it to. So if you’re trying to get an overarching view of how things are then these are good questions. It just gives you a high-level view and then you the way that that works is you then look at that and say where are we doing well, where are we not doing as well. We choose the ones where we’re not doing as well and we look into them more deeply. Or do you want a very specific group of patients from that palliative care perspective and look at those questions that relate to all of those things. Where the pathways have actually been generated out of a lot of work in understanding what people’s needs are and so we can measure them from a process point of view or do we want to measure them from a patient perspective point of view. So it really depends on how you want to use the tool. So these are quite I find these quite useful from a high-level perspective where you can ask a broader number of people and then be able to focus on the things that you don’t do as well.*  *Reference 2 - 0.42% Coverage*  *R1: So I think having something like do you have any comments positive or negative that you would like to add about your experience of being in the hospital as a patient or carer I think that’s important. Because often they don’t get the opportunity to feedback unless you get a letter or whatever.*  *Reference 3 - 1.28% Coverage*  *R: I think yes and no. I think you know things like the patient’s perception of are they being listened to, their surroundings, their confidence in human things are all good feedback for the individual team. I guess some of the more complex things like you know that sort of thing and communication about your plans are fairly complex questions and I just on first thoughts if you get some that are good and some of the bad what do you do at a system level to then. I mean at an individual level if you’re going to say are you looking at these following a patient’s discharge and go oh look we didn’t do very well on that and then you could follow up. Or are you going to say of the 100 who went through some said good and some said bad but how do you interpret that in terms of making a change in your practice that might be a bit harder. But I guess this is a screening tool rather than a more detailed tool.*  *Reference 1 - 7.40% Coverage*  *: How would you react if someone came to you just on a normal day when you are in hospital and asked you to complete the document?*  *R: Depends on what condition I am in. Today I'm in a very good condition. When I came in on Thursday I was in a very, very bad state. So if someone was to come to me on that day I would have probably told them go away.*  *I: Yeah, yeah,. Yeah fair enough. So if you were feeling really unwell you’d totally not be interested.*  *R: No, absolutely. Nobody would be interested.*  *I: Yeah, yeah but if you’re on a day which is not so bad…*  *R: ….yeah I don't mind.*  *I: Yeah. Do you find like you’re saying you don't mind, would it be, is it something that you find would actually be helpful or you would like to do or it’s…..?*  *R: Yeah if it’s providing input that will help the facility then why not? Yeah.*  *Reference 1 - 1.58% Coverage*  *R: Umm…I wouldn't react to it in any special way. It’s just something that hospitals do so it certainly wouldn't upset me.*  *I: Okay so you’d be happy to fill it out?*  *R: Yeah, yeah.*  *Reference 1 - 0.19% Coverage*  *R: Yeah if you want me to.*  *Reference 2 - 1.08% Coverage*  *R: Oh yeah, yeah. Because the answers are there you don't have to like I don't have to think about it you know because they’re just lovely people.*  *Reference 1 - 1.90% Coverage*  *R: I'd be fine, I understand that you’re…things are trying to get better. So this is a way, possibly, of finding out some of the information that you want in order to continually improve. Hopefully find gaps where if you’re not improving where aren’t you improving. That sort of thing, so I understand the reason for it.*  *Reference 1 - 2.02% Coverage*  *R: Well my reaction immediately would be “Hey I'm in pain I'm not going to be bothered filling out a survey”.*  *I: Yeah so to you it’s very important when someone approaches you with something like this the timing of it?*  *R: Yes I didn't come into hospital to fill out paperwork.*  *Reference 2 - 6.19% Coverage*  *R: Well that’s what I said in the first place, there’s a lot of motherhood sort of statements you know that the sort of all encompassing. You need a bit of time to put your thoughts together.*  *I: Yeah so motherhood you’re talking about ahh very…*  *R: All-encompassing sort of…*  *I: So quite broad questions where…*  *R: …well that’s right.*  *I: …it’s not specific.*  *R: No my individual needs were met? Well some of them might have been some of them maybe not I don't know I mean I’d have to think about what you mean by individual needs were met.*  *I: Yeah so when you’re saying they’re quite broad are there any important questions missing from your perspective?*  *R: Not that I can see I mean I'm just glancing at this I can’t really you know to do it…*  *I: …you can take your time.*  *R: ….to do it properly I’d have to have a good sort of look through it.*  *Reference 1 - 0.37% Coverage*  *R: Well if I had someone helping me it wouldn't matter it would be great.*  *Reference 1 - 0.42% Coverage*  *R: I don’t see why not if it’s helping.*  *Reference 1 - 2.49% Coverage*  *R: Umm yeah umm…it’s quick, to the point, there’s not a lot of detail that’s required, I think it’s a good way to do that. It gives you the information……oh well a trivial amount of information very quickly. It’s easy for the user to survey with the survey because it’s just a circle and answer. It’s a great, it’s a good idea.*  *I: Okay so you wouldn't have any issues about completing the survey on behalf of…*  *R: …no…*  *Reference 1 - 0.70% Coverage*  *yeah actually I was a bit surprised as how deeply they are looking into things like this. Yeah. Quite good.*  *Reference 2 - 2.15% Coverage*  *R: I don't know. Laugh I’d have to go through it a bit more thoroughly than I have. To make up my mind about it all.*  *I: Okay. So you’d want, if you were given a survey such as one of these to complete, you’d like to be given time to maybe go through and decide as to whether you’re going to complete it or not?*  *R: Yes, yeah.*  *Reference 1 - 0.22% Coverage*  *R: Alright.*  *I: You’d be happy to complete it?*  *R: Yeah.*  *Reference 2 - 2.05% Coverage*  *R: You see what bothers me is very bad, bad, good, very good, doesn’t apply. I’d like to then have another line underneath where it said something like write down the three words you can think of that would most apply that you would most apply to this thing. Even if it’s just three words that said that you know said brilliant, wonderful and fabulous you’re in a different area to very good.*  *I: Yep.*  *R: Because there are people including me now who really do want to express just how good some of this is*  *Reference 3 - 1.20% Coverage*  *R: I think you should be giving I don’t know how often but I know I would welcome this as a way to say thank you. But how often I don’t know I think it would be perfectly possible to keep enough of a record of who got them so they didn’t get it the second time if they were still here sort of thing.*  *Reference 4 - 0.33% Coverage*  *R: I think it’s very important actually and I think I wouldn’t put those things.*  *Reference 5 - 5.68% Coverage*  *R: Because and I have met some people here including myself you know I don’t know if you’ve ever had a serious illness but I can tell you that when I started walking around without pain, with a totally clear head and every now and then I still even after a year of a totally clear head I haven’t had any pain or painkillers for 17 months. And every now and then I have to go dear God had did I get this lucky sort of thing. And there’s a lot of people I think like that and a lot of people who want to say thank you without it becoming mucky or mirky or you know you can see I tear up at the slightest thing nowadays although I was always a bit teary I must admit. And I know that they would see this as a way of saying thank you. On the other hand if you’d had some bad experiences I think it’s also that idea of putting a phrase or something is also an outlet for people who might go well this was alright and that was alright but they’re a cold bunch or something like that you know.*  *I: Yeah.*  *R: To let off steam without necessarily saying that you’re all mad and bad and to let off steam in different areas as well. Sometimes it’s only someone like that nurse of mine who although she was being truthful because I said it to another nurse and she said well unfortunately it is true but she said but it’s not what causes or stops it. It is true sometimes people die and we haven’t noticed immediately.*  *Reference 1 - 1.44% Coverage*  *R: I would do it.*  *I: You’d be happy to do so?*  *R: Oh yeah.*  *I: Would there be any reservations at all?*  *R: No.*  *Reference 1 - 0.70% Coverage*  *R: I’d say yes.*  *I: Okay. So you’d be happy to do so?*  *R: Yes.*  *I: You’d have no reservations at all?*  *R: No.*  *Reference 1 - 1.31% Coverage*  *R: Yeah there’s no reason why I wouldn’t want to complete it.*  *I: Okay.*  *R: No.*  *I: Alright.*  *R: Because I think it’s the only way you get through to solving problems.*  *Reference 1 - 4.46% Coverage*  *R: Well I’d certainly do it. What would my reaction to the survey be or to the care at the hospital?*  *I: More towards the survey and having to complete one.*  *R: I think if it comes to the quality of patient care I think most patients are pretty satisfied with the sort of care they get and they wouldn’t mind answering a yes to that because I think most of the time it’s going to be yes I’m quite satisfied with the quality.*  *I: Yes. So you’d be happy to complete the survey?*  *R: Yes I think so.*  *Reference 2 - 2.14% Coverage*  *If it helps you. If it helps you with your stats and demographics I think it’s probably a good idea. Just that someone who’s come into the system might have less expertise on the system than someone who’s been in it for a month or two.*  *Reference 1 - 0.46% Coverage*  *R: If I read the contents and it’s about the patient not about hierarchy or whatever yes I would.*  *Reference 2 - 2.14% Coverage*  *R2: Well now alright he probably wouldn’t have last week.*  *R: It depends on ..*  *I: Well it really depends on where you are in your admission?*  *R: It depends yes that’s right yes very much so. It’s like I’ve been here since last Thursday and it’s the situation where it depends on what stage you’re in you know what I mean. I mean to say if you were to come on Friday I’d say see you see you later flick you off. No I wouldn’t have bothered.*  *Reference 3 - 1.44% Coverage*  *R: Well I mean to say you can’t go out to the inpatient and say are you well? Are you good, are you fine, can you do this, are you sure you can do this? You know you’re not going to hit them with that. I don’t think so it’s just that if they accept you they accept you if they don’t they don’t.*  *Reference 1 - 0.28% Coverage*  *R: My personal reaction would be yes okay.*  *Reference 1 - 1.78% Coverage*  *R: Why wouldn't I, I can't understand why would someone say “Fuck off mate and get out of my face because I don't want nothing to do with your shit” why would you feel like that?*  *I: Yeah I suppose…*  *R: …because you’re trying to help other people and improve the system why wouldn't you?*  *Reference 1 - 1.72% Coverage*  *R: How I would now. Because I can only say how I was treated and I’ve been here before.*  *I: Yeah would you be happy to complete it or you’d prefer not to complete it?*  *R: I’d be happy to complete it.* |
| **Feels like criticising staff** | *Reference 1 - 0.28% Coverage*  *R3: I actually think it’s important to give direct feedback but I agree you’re not going to get that from patients unless they feel confident that they can be authentic when they answer this. I think that’s the big barrier.*  *Reference 2 - 0.37% Coverage*  *R3: But I think patients need help to understand that it’s okay to be honest and they need help and support to be guided that they’re not going to be judged by what they’re saying and it’s not going to come back at them. It won’t affect their treatment but I don’t know that they feel that.*  *Reference 3 - 0.21% Coverage*  *So she shut down and just sat there like the good patient but she would tell me that but she would not have felt confident as a patient a vulnerable patient saying that.*  *Reference 4 - 1.58% Coverage*  *R3: Yeah. Fearful to be genuine about your experience.*  *R4: Because the nurses and doctors they’re too important in your care and you start complaining.*  *R3: They worry about the treatment being affected or shut down.*  *R2: I would say there it’s okay to express to us we want to understand both the positive and negative things that you want to share with us to improve our experience.*  *I: Okay.*  *R2: So that sort of lets them down to do that and then you can have a positive and negative so that it’s split into two parts. So the people that are older that think oh you never criticise a hospital, I’ve got parents like that they’ll do anything if you say jump they’ll just jump you know.*  *R3: But a lot of young people think that way to.*  *R2: So say it’s alright to share we want you to share with us both your positive and negative feelings and then have the word positive and the word negative so they can do it under both and that way we’re getting a more balanced approach to it just one thing that they’re remembering at the time. So there is positives about everyone’s experience because you actually made it to hospital and there are negatives as well and I think that better reflects the whole thing when they comment on them both.*  *Reference 1 - 2.06% Coverage*  *In my experience at the moment if I was filling this out right now I would actually put a comment in there and the comment would be positive and it would be that I was very impressed with the way departments have worked together.*  *Reference 1 - 4.15% Coverage*  *R: …what I mean is you’re jumping from …you’re jumping from …it seems to me like you’re looking for a way to put shit on the staff. You’re knocking the hospital, you’re knocking the staff, you’re knocking the tea lady. You want to find something to improve right?*  *I: Yep*  *R: You don't necessarily want to knock them because they’ve only got protocol they’ve got to go by what they’re told to do. But it just….my individual needs were met, my views and concerns were listened to well that’s when the need, could not (member of staff?) explain why? The full ..reading to himself….yeah. Yeah see I’ve done all that and I am fully involved in all of that sort of stuff.* |
| **Hard to be accurate** | *Reference 1 - 1.20% Coverage*  *Umm…my only thought say like on question eleven, my harm or distress was discussed with me by staff. Maybe saying which staff might identify more of who is involved. Because often it is the front line nursing staff or, but it would be nice to know who is doing that more often if there was a you know if there was any form of altercation whatever, who is it that’s commonly doing the, that. Well that sort of discussion as such. And the only other thing I thought was on question eight received pain relief that met my needs. Certainly coming from a palliative care point of view pain is not the only thing. So whether we could broaden it to ‘were my symptoms such as’ because we get patients with chronic nausea and all sorts of other things. So pain is just like one snippet, whether we could broaden that?*  *Reference 2 - 0.69% Coverage*  *R2: I think that could be addressed when you’re looking at question three where it’s asking you to, if your individual needs are met and how it’s saying when not what staff weren’t meeting that need? Because that ambiguous use of the word staff could be nursing staff, could be palliative care. So it might be there’s some space to distinguish which team they felt their needs weren’t being met or who they were getting met by. Because that’s a communication barrier.*  *Reference 3 - 0.39% Coverage*  *because staff they could be angry that the medical team haven’t done it but they love the palliative care team you know. So there is that distinguishing factor of why weren’t their needs met? And it could be at that specific moment that they are doing this one.*  *Reference 4 - 0.34% Coverage*  *if they say my needs are always met, always, they don't need to distinguish because that’s everyone. But in question three where it’s like please elaborate it would be like please elaborate on why you felt, like who was lacking?*  *Reference 5 - 0.71% Coverage*  *R4: Yeah and it may it sometimes as you were saying it may be that the doctors and the nurse and the Allied health are all talking but they’re talking separately. One because it I mean just not be logistically possible for everyone to talk in the same place or two they may be talking outside for, because they don't want to include the patient in that conversation sometimes. So…but I don't have a big issue but I just think that question can be difficult for patients to answer.*  *Reference 1 - 0.55% Coverage*  *R4: One thing I was trying to say earlier there’s always any hospital experience it’s not all bad and it’s not all good and somehow or another you’ve got to get people to reflect on there are some good things that happen and sometimes it is all bad you know. But sometimes you know there are valuable things that hospitals need to feedback they are doing it right rather than investing in areas where everyone thinks they’re okay.*  *Reference 1 - 2.54% Coverage*  *R: I think assessing you know it’s not an unreadable tool I guess remembering now what Elise and I were talking about because I guess what in reality if you get ten of those for our adult patients who are seen the ward. On the whole spectrum from the person who’s dying but still wants to go off and have experimental treatment in general there’s one sitting in here at the moment, he’s been like that every time he comes in, to others who are sort of very accepting. And then others particularly when we’ve had lockdown where we’ve had family members who have been unhappy with communication even though the patient is well aware and one of their family members is their designated contact is aware but that hasn’t been passed on. And so I guess some of it does come down to individuals. How do you know what that patient and their family want and how can we individualise it or in a busy ward is it a bit of this is what we do, we think that’s good for the majority without having that ability to individualise too much? I think people try and individualise but I think sometimes it’s probably hard to know exactly what that family or the person and their family or the carer’s specifically want. You might have a patient who hasn’t told any of their family so the family get really annoyed when they come in and find out that they’re dying but that wasn’t the team’s decision that was the patient’s decision so that’s an extreme. I guess trying to get that useful information which is probably partly system in terms of surroundings and communication and other things. But part of it is a bit of how do you individualise how you manage that person to ensure that if that person wants things done in a certain way that everybody in the term for 24-hour shifts actually knows that and then can do it that way.*  *Reference 1 - 9.25% Coverage*  *I: Yeah and can you point to any of those like specifically or… it just, on the day you might need a little bit of assistance?*  *R: pause See like this. When you need, you could not meet staff explain why. You know umm to me …and about and the decision about my treatment. You know something you have to think about you can’t just say oh whatever. Because you need time to think about it.*  *I: Okay so it takes that time. So for the commission tool with a couple of the questions specifically three and five perhaps that you need that extra time to actually think about those questions and maybe your experience that you have had in hospital.*  *R: Yeah. Yes I …I mean I’ve…I nearly died here two months ago. I was in here for five weeks, three weeks I didn't know who I was. Through chemotherapy the chemo just didn't go for me. And so and I mean they asked me questions and I couldn't tell them anything because I couldn't remember but oh you must remember something you know. It felt like I was being pushed to give an answer that I really couldn't give yeah so…*  *Reference 1 - 0.65% Coverage*  *Pretty much motherhood statements aren’t they, all-encompassing sort of things so yeah.*  *Reference 2 - 6.19% Coverage*  *R: Well that’s what I said in the first place, there’s a lot of motherhood sort of statements you know that the sort of all encompassing. You need a bit of time to put your thoughts together.*  *I: Yeah so motherhood you’re talking about ahh very…*  *R: All-encompassing sort of…*  *I: So quite broad questions where…*  *R: …well that’s right.*  *I: …it’s not specific.*  *R: No my individual needs were met? Well some of them might have been some of them maybe not I don't know I mean I’d have to think about what you mean by individual needs were met.*  *I: Yeah so when you’re saying they’re quite broad are there any important questions missing from your perspective?*  *R: Not that I can see I mean I'm just glancing at this I can’t really you know to do it…*  *I: …you can take your time.*  *R: ….to do it properly I’d have to have a good sort of look through it.*  *Reference 1 - 1.28% Coverage*  *R: I suppose easy enough to fill out but a bit hard to be very accurate.*  *I: Okay and what do you mean by that a bit hard to be accurate?*  *R: Oh well it differs so much from individuals I suppose.*  *Reference 1 - 0.92% Coverage*  *R: Oh I would use that right to the very brim. But also there’s no room for fine lines in a way and there’s you know always, mostly, well sometimes the one thing that isn’t all that great can overwhelm the ten things that were.*  *Reference 2 - 0.46% Coverage*  *: And one of the problems with that is that that’s 24 hours a day and to answer so simply would bother me you know.*  *Reference 1 - 2.10% Coverage*  *R: What about if I didn't have any views or concerns?*  *I: then….*  *R: You know like if I wasn’t umm….it would have to be unhappy to have a view or concern so I couldn't say either, everything here has been brilliant so I can’t you know what I mean?*  *Reference 2 - 5.62% Coverage*  *R: …well how to rate your attention to your physical problems like things like pain, dry mouth or trouble breathing. Very bad, good, very good, doesn’t apply. Well…why can’t you write what really you think your problem is? You know sort of I have trouble breathing because I smoked. It doesn’t mention anything like that you know.*  ***12:54.7***  *I: Yeah*  *R: You know people know that they’re going to have trouble because they smoked, I smoked. You know I know I'm going to you know if the doctor told me “Gee your liver has been playing up you know” like I’d say “Well I gave it a good lashing you know”. So but that doesn’t cover anything you know to me. Alright.* |
| **Subjective nature of PREM data** | *Reference 1 - 5.21% Coverage*  *R3: Yeah so to me there’s it’s working pretty hard when there’s such a vast range of things that people might or might not say with regards to the condition that they’ve got and I’m just wondering how that can be overcome in this. You know some people say the intense pain I had a woman up just the other day while we were here this morning crying and sobbing and then having other reaction to people with her, her relations. I didn’t ask to hear it but I couldn’t help but hear it and had been doing it every day for what about three weeks now not just from my wife at the time so to put that sort of thing into this sort of thing is very hard I think. Not that I’m against it.*  *R: It’s like it’s hard to for people to separate out how the service they’re getting or whatever you want to call it in the hospital the care they’re getting in the hospital from their own pain and illness and those sorts of things. Is that what you mean?*  *Reference 1 - 0.26% Coverage*  *R2: They generally send it out later. A lot are sent out later when you’re not in the actual heat of the moment.*  *R4: But even after her recovery and then she’d probably forget it if you know what I mean.*  *Reference 2 - 0.33% Coverage*  *R1: I feel it’s more person focused even though it’s got those things because if you’ve got physical problems feeling, surrounding, respect, communication those sort of things. It’s more open ended yes.*  *R3: That can make it hard to answer the question.*  *Reference 1 - 2.08% Coverage*  *R1: And a lot of these are fairly generic like how do you know that the staff involved in your care are communicating. You see some communication at bedside. There’s other team meetings held elsewhere that the poor patient isn’t always aware of the discussions well they should be fully aware of what’s being discussed but sometimes a lot of it is generic I suppose.*  *R3: You’ve also got the confounder of patients who’ve got you know underlying psychiatric diseases, personality disorders, splitters. Obviously if you’re doing enough they’ll be a (??) you know like we have that all the time and how that plays into their relationships and whether it influences nurses in the care we have.*  *R1: Increasing level of expectation of care.*  *R3: It’s extraordinary as I said to someone the other day you know we have to go to the bedside and negotiate every milligram of drug that’s being used.*  *R1: And that’s pain relief.*  *R3: And they’re not the experts.*  *R1: It’s never …*  *R3: And it’s so anyway.*  *R1: Some people will never be comfortable with the amount of pain relief. I’m not saying cancer patients but patients generically on some of the wards.*  *I: So from your perspectives that sort of bias that comes through in patient experience data is that do you still think it’s worth pursuing collection of experience data to guide improvement?*  *R1: Absolutely I think the consumer view is absolutely critical. All I’m raising is the day-to-day work that we do there are confounders in that.*  *Reference 2 - 1.14% Coverage*  *R1: So there’s a lot and as the palliative care team we do a lot of very careful communications. So things had been said by one team and they would get to the bedside they don’t really understand we go back over all of that again. Then there are the patients that get information don’t get any further explanation. It creates difficulties. And then you know there’s so many confounders in here and a sense of people being entitled and all this individualism and you know this wasn’t said and that was said. And one member of the family gets a piece of information but the other person doesn’t so you know it’s just not a straight forward sort of reflection. I don’t know whether you couple the interviews with a chart review or something just to be sure that you’ve got some other background information.*  *Reference 3 - 0.55% Coverage*  *R3: Look it’s a useful tool but it’s one of a set of things that I would use. I also think [name] would see complaints. So the consistency of what I see about communication or you know you might get a complaint that my needs weren’t cared for, I was in pain. You know I couldn’t get someone to answer my buzzer and that so it is a set of things that are important that I want to know about.*  *Reference 4 - 0.37% Coverage*  *R1: And so I think it’s good and it’s a screen isn’t it? This is a screen that’s all I see it as it’s a screen you’re obviously going to have most people fall sort of in the middle and then you’ll get the people you’ll identify who will come with distress or whatever.*  *Reference 5 - 0.31% Coverage*  *R: It’s a little bit of perception. I guess one of the issues of those sort of “how do you rate attention to affairs” and things may be rated quite differently by different people. I presume this had been validated has it?*  *Reference 1 - 4.65% Coverage*  *Did you find if you were to fill it out like obviously we don’t require you to fill it out for this phase of the study but if you were to fill it out would you find it relatively easy to fill out?*  *R: Yes although I’m not sure it covers the situation for me.*  *I: What do you mean by that?*  *R: Well for example there are aspects to feeling well and to feeling treated well that may be questioned about. Like “I experienced unexpected harm or distress” seems somewhat harsh but also a lot of the significances to me in a hospital have to do with the ambiance. With for example I think a lot has been lost with masks because for me coming to the hospital especially one time I came 35 times in seven weeks for radiation and when you are having something difficult people smiling at you is very I mean people walked by and smiled, people who were sweeping floor would smile at you. It seemed a bit policy you know.*  *I: Yeah.*  *R: And I’ve been astounded by the maintenance of the morale and sense of humour of the place as well. I don’t know that some of those sorts of things I don’t think are asked about.*  *I: Are asked about.*  *R: It’s very it’s clinical.*  *Reference 2 - 2.70% Coverage*  *R: No because if you read this question if I was the carer and you were the patient and I read “how would you rate our attention to your physical problems” maybe instead of our it should say the hospital’s treat the hospital as a body.*  *I: Yeah.*  *R: Our I must admit even when I first saw it I wondered why it was “our” and that makes it and certainly if someone was reading the question to you and said “our attention” well you’re going to say oh if I say something negative it’s going to insult this person. So I think the “our” should go even if it’s not you know only the patient answering because it’s easier it is easier to be negative to a body of an organisation.*  *Reference 1 - 5.56% Coverage*  *R: I mean to say is “how would you rate your attention to your surroundings like things like noise, light and warmth” to me that’s stupid. A lot of people out there are pretty they don’t like a blind open. I love a blind open. It is what it is you know “physical problems things like a pain, dry mouth or trouble breathing” well that’s me trouble breathing. “Rate your respect to what matters to you” well it does but you know there’s things that get on people’s nerves, niggle things whatever. “Communication” I try to communicate the best I can you know things like that I’m only going through these. “Attention to affairs” I think that’s I think if the person asks about “affairs” depends on what affairs like financial you know with people other side of it you do ask yourself, you ask someone you know to say look I’m in hospital I can’t afford this, my rent’s due this and that that’s affairs. But that’s family and then from there if family can’t help then you go from somewhere else. “How do you rate your attention to what you expect” things like getting worse like time to live and die that what I just explained well that’s my opinion.*  *Reference 2 - 1.99% Coverage*  *R: Well I mean to say that kind of outlays it a certain degree. Like “my needs were met” you know say if I asked for you know I want a coffee, I always got a coffee, mostly I got a coffee, sometimes I got a coffee, rarely I got a coffee, I never got a coffee but I asked the question could I have a coffee. And it’s up to the person that I asked to get me the bloody coffee and it might be different each time.*  *Reference 3 - 0.78% Coverage*  *R: And then their experience while they’re in here and if they’re what do I say? Well everyone’s different you know it’s just a situation where it’s bloody hard.*  *Reference 1 - 8.14% Coverage*  *R: I think the only one that I sort of would question a little bit is “that I felt cared for”. It’s one that’s open to a lot of interpretation.*  *I: Okay.*  *R: And I don’t have a suggestion as in terms of a change but it probably just it’s probably something more along the lines of clarifying what that care is about. So whether it’s about you know I felt that they cared for my welfare or you know that kind of stuff. It’s a hard one because it’s a really tricky one.*  *I: Okay. So along the lines …*  *R: And I’m just thinking of that in terms of you know the variety of people that you’d be asking that question to and their current state of wellbeing and stuff.*  *I: So their interpretation of care can differ quite significantly from somebody who is coming in for like for instance like an appendectomy where they come in via Emergency because they’ve got a bit of pain and they end up having their appendix removed and they go home relatively quickly.*  *R: Well I mean it’s a very broad range of sort of stuff. For me I read it as being I felt cared for in that while all of these other things are going on that they still had my best interests at heart kind of thing.*  *I: We added that …*  *R: In the clinical environment.* |
| **Identifiers for patient groups** | *Reference 1 - 2.40% Coverage*  *R: So you wouldn’t know if they’re an Aboriginal person or not or anything like that?*  *I: So it’s not so much looking at demographics or for instance like looking at location geographics.*  *R: It’s more just how they feel about …*  *I: It’s about the experience that everyone has.*  *R: Because obviously that would be interesting with different groups feel quite differently about their experience but that’s a different issue I suppose.* |
| **Need assistance to complete** | *Reference 1 - 0.25% Coverage*  *Umm… I’d probably need a bit of a hand to go through it properly.*  *Reference 2 - 0.60% Coverage*  *I: And would you want assistance in completing it or are you happy to complete it…*  *R: ..no I’d be happy…*  *I: ..on your own?*  *R: …to do it myself yeah.*  *Reference 1 - 1.24% Coverage*  *And would you need assistance in completing it or you’d be quite happy to do it yourself?*  *R: Quite happy to do it.*  *Reference 1 - 0.08% Coverage*  *R: No that’s fine.*  *Reference 1 - 0.27% Coverage*  *R: Oh no I wouldn’t want assistance.*  *Reference 1 - 1.34% Coverage*  *R2: I think if someone had a serious illness they’d find it hard to engage with a survey period. I can’t think of some of the patients in [name]’s Mum’s bay I don’t think any of them would be able to engage with this because they’re so sick.*  *Reference 1 - 1.58% Coverage*  *R: It was pretty hard because I didn’t know what I was expecting so pretty hard but at least now what I’m expecting it would probably make it a bit easier next time. Like if you said to me come down on Friday and I was to come back again I’d probably understand what I was trying to complete.*  *Reference 1 - 0.95% Coverage*  *R1: And yeah I agree as well but by the time like the screening process for who completes this like by the time a lot of our cohort on my ward and probably everywhere really, but at that terminal phase on the clinical guidance the dying patient, they’re not necessarily in any position to be…so it will have to be earlier on.*  *R: ….yeah it would have to be earlier on in the journey. Like once yeah we know when that terminal phase that that communication is going to diminish. Sometimes we have it for a long time but depending on the condition, yeah we may lose the capacity weeks in advance. So it’s really it’s not always a clear yeah.*  *Reference 2 - 1.07% Coverage*  *R: It would be a mix. I think.*  *R1: Yeah it would depend on the patient.*  *R2: Yeah it would be dependent on the patient. Some of these could be triggering to them if they’re very upset. Just from like an actual physical ability to fill out any form I would have a limited amount of my cohort that could do any of this. Umm…I think one page is always easier and I think that having the prompt of you know taking a bit of the ambiguity out of the question by then giving it, the patient the prompt afterwards of the types of things we’re looking for will help as well. Exactly how you said [name] about I felt cared for. Like…I don't know I just didn't or it was great but like they can’t…*  *R4: …depends on which day…*  *Reference 3 - 1.31% Coverage*  *R3: From a clinical point of view this one.*  *I: Considerate? Yep.*  *R3: Yeah but for the reasons I mentioned before I think. You know some patients will not highlight you know their concerns about what’s happening and sometimes they’ll be more willing to do it in a form. But I think as well you have to think about who would help them fill out the form. Because if they’ve got you know medical staff or the people that have been caring for them and then they’re saying you know very bad for attention to their feelings. So maybe a little bit less willing to write that if the person caring for them is sitting in front of them. Particularly if there’s you know if the information is going to be used at a higher level and you know..for those sorts of things. So does it need to be someone independent or do they need to fill it out themselves? I don't know but that might be an issue.*  *Reference 4 - 0.18% Coverage*  *R1: So they have a 12.30 staff who actually, their job is to get that survey, our ten surveys in a month get them done.*  *Reference 5 - 1.77% Coverage*  *R2: So I do know for the [name of Hospital] that they are sort of in the intervention or not intervention stage because they’ve started rolling it out, the prems and proms across Queensland health. So the [name of hospital] for outpatients with gen-surg which has come on board as of I think it was September this year. So and that’s voluntary by patients who have attended, they’ll get a text message. And if they reply to that then obviously they complete the survey if they’d like to. And gen-surg in-patients have had the same well the same set of measures since I think last year. So I assumed that that’s something that’s going to get rolled out across other divisions as well against the like patient experiences, so the prems and proms. So reported experiences, reported outcomes.*  ***59:54.7***  *R1: I think that has rolled out. Is that on discharge the sort of text they get to then…*  *R2: Yeah so it’s through Quest Link. Yeah so but for the [name of hospital], as far as I'm aware because I’ve looked at the protocol, it’s only been gen-surg.*  *R1: It’s only oh okay maybe it’s just…*  *R2: …outpatients and in-patients.*  *R1: Yeah okay.*  *R: I think [name of childrens Hospital] is doing it.*  *Reference 1 - 0.32% Coverage*  *R1: The issue I have with just handing these out is I think patients need to be prepared. Communicated in some way about what these surveys mean because I don’t think my mother would have done this properly and she hadn’t lost capacity or anything.*  *Reference 2 - 0.11% Coverage*  *R4: I really think you need someone to go through them with you.*  *R3: It needs explaining.*  *Reference 3 - 0.25% Coverage*  *R4: If it has to be a hospital volunteer because there’s no one else but you know maybe that but I think someone needs to talk to people about this to help them focus on what they’re really feeling.*  *Reference 4 - 0.60% Coverage*  *R4: You get a different response if you’ve got a family member or a carer answering that with the person. You’ll get a different response even if you’ve got a volunteer or someone the person doesn’t know it’s that trust I can say this can’t I or what about this and I can put that in if there’s someone who’s been though the experience with you like your carer or your daughter or whatever. I think it’s really important that it is someone you how else can that happen?*  *Reference 5 - 1.06% Coverage*  *R5: OT’s doing an IT test on you they don’t just throw you three sheets, they work though each question with you to work the sheets down when they’re doing it to see if I’ve got good cognitive impairment and that’s that explanation helps elevates the fears, helps get the right question in. So people understand and they can ask questions about the question and then get the feeling that they’re in a space where they can give an honest answer.*  *R3: Because people can read and interpret questions differently as well depending on their background and their mental capacity. They can completely misunderstand what is being asked. So I’m like [name] so prepping someone to go through if you’re going to use this one to go through it step by step with each question so that they have a good understanding of what they’re responding to.*  *Reference 1 - 0.94% Coverage*  *I can’t even write steadily because of the chemo. So… I don't know if you will be able to read my writing.*  *Reference 2 - 1.47% Coverage*  *Do, would you feel any need for assistance from a nurse or from somebody like a volunteer or you’d be happy to do it by yourself?*  *R: No I’d be happy doing it myself.*  *Reference 1 - 3.09% Coverage*  *R: No I can complete it but maybe there’s…my education is not very high. Turn around and I’d probably ask John “Oh what does this question mean” ?*  *I: Okay so just to clarify it. So with regards to so the words you don't find confusing but maybe some of the questions you might find difficult…*  *R: …yeah…*  *I: …in grasping…*  *R: …yeah the meaning of them.*  *Reference 1 - 0.34% Coverage*  *R: No I’d be happy to complete it on my own.*  *Reference 1 - 0.09% Coverage*  *R: I’d be fine.*  *Reference 1 - 1.00% Coverage*  *R: Yeah I’ve got no problems with writing and expressing my thoughts. I’ve filled out many surveys along with feedback forms in the past.*  *Reference 2 - 0.18% Coverage*  *R: Oh I’d do it myself.*  *Reference 1 - 3.98% Coverage*  *R: I would just ask you to help me.*  *I: Okay so just you’d need a little bit of assistance but you’d be more than happy to complete it?*  *R: Oh yeah, yeah. Oh yeah.*  *I: Okay. Now with regards to that survey would you find it relatively easy to fill out? You said you’d need someone to help you…*  *R: …me no love I need help.*  *I: Okay*  *R: Because I can't spell much. What I do but I'm teaching myself and I am getting a little bit better. I break it up into syllables.*  *I: Okay so…*  *R: …sometimes I spell it right.*  *I: Yeah. So if you were to have assistance and someone just helping you with that survey…*  *R: I would do it.*  *I: You would do it and you’d find it relatively easy? Like do you prefer to have…*  *R: Well if I had someone helping me it wouldn't matter it would be great.*  *Reference 2 - 0.77% Coverage*  *R: I couldn't write no.*  *I: Yeah because of like you are limited…*  *R: ..the only way I can do it is yeah, yeah. It’s a bit hard to write at times love.*  *Reference 3 - 0.29% Coverage*  *R: Yeah well they would have to I couldn't do it love.*  *Reference 1 - 0.25% Coverage*  *R: No I’d do it myself.*  *Reference 1 - 0.17% Coverage*  *R: No happy to do it myself.*  *Reference 2 - 2.38% Coverage*  *And that’s you know embarrassment in regards to hand writing skills and embarrassment to spelling and the embarrassment of you don't know what’s available. Education is probably a big thing, you don't know what is available through Queensland Health. Obviously …*  *I: So having that as an option of these are the services that we can offer you…*  *R: …,yeah do you feel like you need more assistance.*  *Reference 1 - 0.68% Coverage*  *R: Ahh…well no I most likely to complete it myself just get somebody to give me a hand when I needed it.*  *Reference 1 - 2.38% Coverage*  *R: I think I’d be I wouldn’t want anyone else to help me because each of these questions how do they start? Actually that’s the other huge difference I should have noticed immediately every one of these questions asks how would you rate, how would you rate, how would you rate, how would you rate not how would you and your friends or how would you and the buddy in the bed over there rate but how would you rate? And for me I would feel compelled to answer it myself even if I was buddy with someone in the next bed and saying to them did you have some trouble with that nurse that time.*  *Reference 1 - 1.74% Coverage*  *I: And what about would you like assistance in completing it or do you feel you’d be okay doing it yourself?*  *R: I’d be okay doing it.*  *Reference 1 - 0.41% Coverage*  *R: No, no, no I can …*  *I: You can do it yourself?*  *R: Yeah, yeah.*  *Reference 1 - 0.16% Coverage*  *R: Yeah I’d be okay.*  *Reference 1 - 0.14% Coverage*  *R: I’ll be fine.*  *Reference 1 - 1.91% Coverage*  *R: No I couldn’t have done it no helpless and you know it depends on what state I’m in to you know it’s my state.*  *R2: Depends on how sick you are.*  *R: Yeah depends on how crook because I have chemo every three weeks to and that destroys you and you’re really not thinking on a level playing field you’re really not.*  *R2: You would need a partner.*  *R: I’d need a partner to do something like that.*  *Reference 1 - 0.82% Coverage*  *R: Personally not necessarily but that’s obviously something that comes down to physical ability and all that sort of stuff.*  *Reference 1 - 0.24% Coverage*  *R: I prefer to do it myself.* |
| **Paper Vs Electronic completion** | *Reference 1 - 0.61% Coverage*  *: ..No, no, no, no. Now if I was to give you that survey to fill out would you prefer it in paper or online?*  *R: On paper.*  *I: Paper?*  *R: I'm a paper girl.*  *Reference 1 - 0.63% Coverage*  *Would you prefer it in paper or electronic?*  *R: In paper.*  *Reference 1 - 0.04% Coverage*  *R: Paper.*  *Reference 1 - 1.03% Coverage*  *R: I like the old paper one.*  *I: You like paper.*  *R: Yeah although I’ve done plenty of format on digital.*  *I: But you do prefer paper?*  *R: Yeah.*  *Reference 1 - 1.06% Coverage*  *R: Well I mainly work on a laptop so an editable pdf where you have radio buttons for the selection would be my preference.*  *Reference 1 - 0.90% Coverage*  *R2: I would prefer tablet but [name] you might prefer it paper based rather than …*  ***16:02:6***  *R3: I think I would actually.*  *R: Yeah some people would definitely.*  *Reference 1 - 0.65% Coverage*  *R: I like the paper form so it’s easy but the tablet’s fine. I’ve got an iPad at home. I’ve got a new laptop at home.*  *Reference 1 - 0.40% Coverage*  *R: You’d better give it to me in paper love.*  *I: Paper.*  *R: The tablet form is out of the question.*  *Reference 1 - 0.82% Coverage*  *R1: I don't want any more paper. Laughter*  *R: I think a tablet would be certainly like you know most people can tap away on them pretty easily. Writing on a tablet though is a pain in the backside. You know I hate it.*  *R2: Or a (?) if that’s possible.*  *R1: to do it on their phone?*  *R2: Yeah to do on their phone, there’s a few websites that….*  *R: …just link straight to it that they can…*  *R2: …where they’re using Q R codes.*  *R: …do it yeah.*  *R1: Yeah like I mean I don't really mind but I think if we can try to go paper free that would be ideal.*  *Reference 2 - 1.65% Coverage*  *R: I think they have different, I think there’s some different benefits in each of them…*  *R4: ..it depends on what you’re aiming for…*  *R: …yeah in what you’re aiming for.*  *R4: Like in terms of the information that you are wanting to get I think considerate would be useful and you could implement it at whatever stage they are in. This would be easily accessible through an iPad like easier to tick than this kind of one. So it could be used easily through someone with an iPad. And I like the family being able to be involved without skewing too much with the ambiguity of the commission one. So the considerate would give you specific and user friendly things and I think it doesn’t like it’s pretty objective data versus subjective which you’re going to be getting from the commission based. I like this one.*  *I: You like considerate okay.*  *R: I think it’s got a few more palliative driven sort of questions.*  *I: Very much so.*  *R: Which from a palliative care point of view we would find of value yeah.*  *R4: This one would be good for every other patient that I have. That one would be better for my palliatives.*  *Reference 1 - 0.74% Coverage*  *R2: I think you’ve got to cater for all the different generations so it’s got to be a mix.*  *R1: So many people who don’t even have phone or a tablet.*  *R3: And there’s a high percentage that couldn’t complete it.*  *R1: There’s nothing like paper you know. It’s like people I know that we come around and we ask them what they want to eat but people used to like filling out the meal things. So if people have the ability to do it or someone at the bedside can help you know it’s hard to get compliance isn’t it with these things.*  *Reference 1 - 0.79% Coverage*  *R: No.*  *I: Happy either way? So would you be comfortable with a tablet that came around?*  *Reference 1 - 1.61% Coverage*  *: Well paper.*  *I: You prefer paper?*  *R: It’s more in my face to do it rather than umm a computer. Because a computer means nothing to me.*  *I: Okay you prefer paper form.*  *R: Yeah,. Yeah.*  *Reference 1 - 1.46% Coverage*  *R: Umm…electronically I think because you can just get your pen and go doop doop doop and you’re not trying to get the paper on a hard surface you know.*  *I: Yep so just tapping the screen?*  *R: Yeah*  *Reference 1 - 1.23% Coverage*  *I: Okay. So at the start of the interview we spoke about well you asked about paper or electronic. So if you were given this survey to complete would you prefer it in electronic then?*  *R: I probably would yes.*  *Reference 1 - 1.04% Coverage*  *: Oh well probably paper I mean I can use a computer but I don't necessarily think that these sort of things have got to all be computerised.*  *Reference 1 - 0.47% Coverage*  *R: ..well I don't know how to use them love.*  *I: Okay so you’d prefer the paper form?*  *R: Mmm*  *Reference 1 - 2.13% Coverage*  *R: In paper. I know it’s done electronically because I mean to say I do have an iPad and everything but I’m not …*  *I: Up to par with that?*  *R: Yes. I mean to say I can do lots of things on there.*  *Reference 1 - 1.36% Coverage*  *R: Oh I’d like to have an option but more than likely myself as a text or an email for myself personally. Obviously people, older people prefer paper but it’s easier for me to do it as a…*  *I: So being given that option?*  *R: Yeah*  *Reference 1 - 0.59% Coverage*  *R: Most likely paper.*  *I: Paper?*  *R: Mmm I'm not a big computer person in the first place.*  *Reference 1 - 4.19% Coverage*  *R: Given that a lot of well see I’m strange and bias because I’m a paper person now a lot of people who are going to answer this one I think would be round about my age. I also think devices have a even when you like them and you get used to them they have a slightly cold feeling.*  *I: Are they not as personal you mean?*  *R: Yeah and a piece of paper and the other thing with a piece of paper is that or two pieces of paper is that you can easily turn them over, you can do what I did is just look at all these questions and say what are these people asking me. It’s not so easy on a device especially if it’s a phone because they’re smaller and I have to tell you I never take surveys on the computer. I certainly wouldn’t on my phone.*  ***32:04:3***  *I: Yeah.*  *R: Simply because I thought of think they’re connected to 10,700,000 people out there why do they want my opinion? No thanks. But a piece of paper handed to you is it’s a bit more tactile. I know it’s old fashioned but even so I don’t think even a kid minds a piece of paper.*  *Reference 1 - 1.44% Coverage*  *R: Electronic easily.*  *I: You’re happy with electronic?*  *R: Yeah because you can think about it and rub it out.*  *Reference 1 - 0.72% Coverage*  *R: It doesn’t matter, I don't mind yeah…*  *I: ….you don't….either way for you…*  *R: … I can just tick this one yeah.*  *Reference 1 - 0.67% Coverage*  *R: It’s probably just easier to do it on the form.*  *I: On the form in paper?*  *R: Yeah.*  *Reference 1 - 3.60% Coverage*  *R: I actually quite like the paper.*  *I: Okay.*  ***12:37:5***  *R: But I don’t think it matters either or.*  *I: Okay. And would you like assistance with …*  *R: Only because it’s here and someone’s just given it to you and a pen and it’s like okay it’s easy do it yeah. I think a lot of people would like that to. A lot of people and certainly a lot of people that aren’t as digital would probably like that.*  *Reference 1 - 0.79% Coverage*  *R: Paper.*  *I: Paper.*  *R: I’m not savvy. I try to be.*  *R2: Well what if they can’t write? He can write.*  *R: Yeah. I can print. I can take my time printing.*  *Reference 1 - 0.14% Coverage*  *R: Personally digital.*  *Reference 1 - 1.36% Coverage*  *R: Because I'm old fashioned I could go either way but I know that a lot of people want it electronically. I know my Mrs would, she does everything…everything that we’ve done in this hospital…*  *I: …has been electronic?*  *Reference 1 - 0.54% Coverage*  *R: No on paper.*  *I: On paper?*  *R: yeah*  *Reference 1 - 0.66% Coverage*  *R: …I’d do it on paper because I don't know what you are writing. No I don't.* |
| **Patients need to feel this information is for a reason** | *Reference 1 - 2.11% Coverage*  *R1: …because those forms are probably getting not filled out because people with chronic care chronic illness like myself either are too sick to do it or can’t be bothered. Because your voice isn’t heard. So I know listening to patients at renal that are on dialysis I'm there, a lot of the time I say with mum, sitting in the waiting room waiting to go on dialysis. They all vent to me and I speak on their behalf because they’re elderly and they just can’t, nothing changes. So why, what’s the point, it never has changed it won’t change.*  *Reference 2 - 3.62% Coverage*  *…you don't know where they go from here.*  *I: …You don’t know where they go and the results from those surveys and..*  *R1: Well yeah like you don't hear back or you don't…*  *R: …no and we’ve done many.*  *R1: We’ve done many and you know the nurses say if you have an issue here’s a leaflet. And they hand you a leaflet and you know you go onto the….and most of them you have to go onto a web address. Most like come on most patients well in my experience over in dialysis, are elderly. Aren’t going to go onto a http.w.gov/ you know like a thirteen letter web page to fill out a survey.*  *I: So that was a comment so what you just said is something that someone has already said to me today is that whole aspect of it would be nice to be given the choice to receive feedback from my feedback. Like what’s happened from these surveys…*  *R: Yeah*  *R1: Yeah that’s right you never know because they just collect them all and that’s it.*  *Reference 3 - 1.37% Coverage*  *R1: I would be happy to put my name to it and not be anonymous if I knew that I was going to get a text message….*  ***12:17:9***  *R: …and even if you tick the feedback you never get the feedback.*  *I: Okay.*  *R: We’ve done that before.*  *R1: You know if you want to learn…*  *R: …and never heard nothing.*  *I: Yeah*  *R1: …remain anonymous let that be an option.*  *Reference 4 - 0.93% Coverage*  *R: Like I’d like to know once the survey is all done what is going to be the action after that, like what is the process after that? Is it just done and then just swept under the carpet onto somebody’s desk and then it’s just forgotten?*  *Reference 5 - 1.18% Coverage*  *R: No sense doing a survey that is going to be…*  *R1: ..and even spoken to like not just…*  *I: …filed away…*  *R1: ….sent a generic text. You know thank you for your like I can understand getting that first. But then if you are raising serious issues a phone call. So you could speak to someone you know.*  *Reference 1 - 3.86% Coverage*  *R: So again with this one you know this might prompt people to want to say more about what’s worrying them maybe from the patient’s perspective here if someone said you know how do you rate our attention to your physical problems? And you know if I said very bad …*  *R2: Wouldn’t you want to know what that was.*  *R: It would be good to be able to express that to the staff who could actually do something about that and it might be for a really specific thing you know like my heel was rubbing. Like a guy in Mum’s other ward you know just his heel was rubbing on the bed a certain way that was painful or whatever and he carried on for a really long time and then that was fixed it was so easy.*  *Reference 2 - 0.47% Coverage*  *: So how would the information be used? Would it come back as data to the hospital?*  *Reference 1 - 0.24% Coverage*  *R5: I’ve been on a few surveys. My experience was once you have a communication process going you get a lot more out of the questions.*  *R3: I’m big on communication.*  *R5: A lot more value.*  *Reference 1 - 0.81% Coverage*  *R3: I like this and I don’t have a problem with qualitative I just think that when you want to do it it’s very rich. I mean I love qualitative data but it takes it depends on how you’re going to use it because if you want to respond to that because you don’t want to collect it if you’re not going to respond to it. It just needs to have a lot of thought about how you’re going to do that.*  *R1: You’re talking about the ethics of getting someone to write?*  *R3: No I’m talking about how do you use the data looking at it from a data analysis.*  *R1: Okay.*  *R3: Not from the individual.*  *Reference 1 - 4.73% Coverage*  *R: Well I’d like to…get some sort of ahh responses to what actually was the result of the survey.*  *I: Okay*  *R: So that you’re not just ticking boxes or making comments and they’re going into some deep dark hole somewhere.*  *I: Yeah so you actually want to see what action came about from that survey?*  *R: Yeah, yeah.*  *I: So is that something like having the option of being informed as to like quality improvement initiatives or…*  *R: Yeah, yeah something like that…*  *I: …and being kept in the loop as such?*  ***15:32.9***  *R: Yeah.*  *I: Okay.*  *R: Just so that you say ‘oh well I completed that survey I was involved in that survey and this is what happened’.*  *Reference 2 - 9.31% Coverage*  *R: …well I wouldn't be worried about any comments that I make being used against me. Because I would expect that any comments that I made would be a fair assessment from my point of view. Therefore you know if you don't like what I'm saying well too bloody bad. But I wouldn't expect it to be used against me. Anyhow it’s a pretty poor set-up if that’s what happens. But no I would like to think if I’ve devoted half an hour or an hour to filling out a survey or whatever that I would get some sort of return feedback to say well this is what resulted from participance in this survey. This is the actions that we’re taking or this is what people didn't agree with or whatever you know. Just some sort of …I mean I’ve filled out like everyone else filled out surveys in the past. But I mean you send them away and that’s the last you ever hear of it. So did I waste my time? You know and with all this sort of end of life stuff you’re talking about people that really haven’t got a lot of time to spare. So we don't want to be spending hours and hours bogged down on paperwork. But I mean I would agree with trying to help somebody in the future if we can improve the services. But I'm not going to fill out a survey if it’s just going to be thrown in a too hard basket somewhere.*  *Reference 1 - 5.10% Coverage*  *R: Yeah look I find the frustrations about surveys not so much in regards to the hospitals. But most of the time the feedback from surveys is a mucky process and you get sick of getting surveyed about how good their service is because it’s just a marketing thing and it just gets your email address. Obviously a hospital is not like that and I understand that but yeah that’s the, you get frustrated the amount of surveys you have to have been asked to do. And I don't do it anymore because of that.*  *I: Yeah so it’s you want to actually see that there’s going to be some sort of benefit from the survey?*  *R: Yeah that’s right and feedback is not so much sort of thank you for giving us a good mark or a bad mark and just you know we’ve taken account of your response. And we’ll keep in touch with regards to how it’s progressing or something like that.*  *Reference 1 - 1.76% Coverage*  *R: Oh ahh no I suppose if you get a good survey they do a world of good yes. So from that point of view yes they should be. Just the sort of surveys we’ve filled out in the past you find there’s no result comes from them. You wonder whether it’s a waste of time or not.*  *Reference 1 - 1.87% Coverage*  *I: So are you suggesting having questions like if you’d like further information on this, this and this like sort of ticking the boxes?*  *R: There should be yes.*  *I: So we can sort of refer you on to those areas of your interest?*  *R: Yes.* |
| **Perspectives on survey for all Vs for palliative care** | *Reference 1 - 0.55% Coverage*  *I think anybody that comes into hospital is entitled to it yeah doesn’t matter how sick they are or what they’re here for I believe you know.*  *Reference 2 - 0.49% Coverage*  *Honestly I think the main issue is they need to listen to what these patients and what they’re saying that is the main issue.*  *Reference 3 - 3.94% Coverage*  *Like [name of patient] can ask for something a half a dozen times or push a buzzer half a dozen times but no one listens. And then you get the same old story.*  *I: So communication is quite…*  *R:. …communication number one.*  *I: So in the commission tool sorry I’ll just, so there is a few questions around communication. So for instance ‘I was involved in wanting to make decisions about my treatment and care’ so communication between treating team and the patients. When a need couldn’t be met staff explained why so that communication again. Um…*  *R: But that’s where it lacks…*  *I: …or my fears and concerns were listened to…*  *R: …yeah but that’s ….*  *I: …so those questions so you feel those questions are okay but in your experience and in [name of patient]’s experience that’s where we’re lacking?*  *R: Yeah.*  *I: Okay. But those, you’re happy with those questions there’s nothing sort of additional that you’d want added when it comes to communication questions?*  *R: No. I just listening and following through…*  *Reference 1 - 7.50% Coverage*  *So this survey specifically so the Commission Survey isn’t designed for people specifically living isn’t designed specifically for people who are living with a serious illness do you think that matters at all?*  *R: No.*  *I: No. So the survey is designed just for the general public so in general. For a carer for someone who does have a serious illness are there any questions that you think could potentially be missing from that survey that you would like to be seen in there or do you think it covers everything from your perspective?*  *R: You’re just as involved as you can be involved. So I find that the doctors have been phoning me and telling me what’s been going on so I’ve felt quite involved.*  *Reference 2 - 6.82% Coverage*  *R: I mean he’s been pretty good but he is very unwell and he’s quite tormented you know. They haven’t discussed this with him.*  *I: Okay.*  *R: And I know that he hasn’t got a long time to live.*  *I: Because of your experience?*  *R: Yes and I’ve seen him going downhill and I was talking to him this morning and I said look if you had a heart attack or if something that you want to be in there for you know do you want to come in and …*  *I: Resuscitate?*  *R: Resuscitate. Would you want him just to leave you? And he says no I don’t want them to resuscitate and that’s what I would like to discuss with someone. I don’t want him to do that.*  *Reference 3 - 4.26% Coverage*  *I: With regards to the survey itself if you were given the choice to complete one of the surveys which one would you prefer to choose?*  *R: This one.*  *I: So the Considerate?*  *R: Yeah.*  *I: Why would you take preference over the Considerate Tool?*  *R: I think it just I just find it more that it the other one’s quite general. This one tends to focus more on really the critically ill people.*  ***08:04:5***  *Reference 1 - 0.95% Coverage*  *R: No not really for whatever reason you’re in hospital. Even if he was in here to have a baby no matter what it is if you’re in hospital, you’re being cared for the questions basically are the same. I don’t think serious illness comes into it.*  *Reference 2 - 1.26% Coverage*  *R: No I think if there was something it would be a very individual thing. You know sort of for every 50 patients you might find someone who’s got another question they could add to it if they really put their mind to it but everyone’s case is different by the same token. Everyone’s needs, everyone’s wants, everyone’s case.*  *Reference 3 - 1.12% Coverage*  *R: Well when you think the format could have had those words there cross ways with a slash or whatever I reckon it’s been put there for that. But if you’ve got something that’s really a lengthy comment or concern yeah that page is fine but for the most part I think I’d go on the front one.*  *Reference 1 - 3.89% Coverage*  *So it’s not designed for people who are living with serious chronic illness. Do you think that matters at all?*  *R: I think it does.*  *I: And why is that?*  *R: Well I mean he’s got a serious illness and naturally you know we want to make sure that he’s well cared for and the people that are caring for him are sympathetic to his needs.*  *I: Okay. So do you think there are questions that might be missing from the survey themselves that could sort of look at that side of things?*  *R: No I don’t think so because it really covers you know everything.*  *Reference 2 - 1.87% Coverage*  *R: This is really I think all those questions they cover pretty well everything that you have to think about.*  *I: And that’s what you’re referring to the Considerate Tool there?*  *R: Oh definitely yes. I definitely like the set up of that one a whole lot better.*  *Reference 1 - 3.01% Coverage*  *R: In terms of would this could this be improved for people with a chronic illness or serious illness you know I was just thinking they’re often depending on where they’re at with their illness very engaged with the institution of the hospital. Like they kind of know something of the routines and those sorts of things if their illness means they’re coming in and out of hospital and all of this is still relevant to that. But I do wonder you could actually sort of you know this is about a single I suppose it’s about what could be improved.*  *Reference 1 - 3.10% Coverage*  *R: And I think it should have been delved into a bit more. I mean I know he’s not the easiest patient in the world to get on with like for going to do things he puts them off. But yeah I think I suppose you’re dealing with different people you know. You’ve got him and then you’ve got another bloke who says oh this is what’s wrong with me you know can they make it quicker that they find out this things. I mean I’m going to lose him and I know there’s not one thing in this world that I can do to stop it.*  *I: So with that being said do you think there’s a question there that’s potentially missing around the communication side of things?*  *R: Yeah I do. I mean it’s not you can’t blame an organisation all the time but in his case yeah I can’t work that out.*  *Reference 1 - 0.51% Coverage*  *R3: I think they will view it when they look at it in the seriousness of their condition I think it is ambiguous enough that it allows for that.*  ***32:50.7***  *R: Yeah I think if you tried to get a bit more specific about their condition or prognosis or, I think that could get a bit messy, I don't know. I don't …I don't know how I’d approach that.*  *Reference 1 - 0.12% Coverage*  *R3: And I would say probably not because I think as [name] said it’s fairly generic.*  *Reference 1 - 4.34% Coverage*  *I: No? So this is an important question this particular survey was not developed for people with serious illnesses, it was developed for any person in hospital. Do you think this matters?*  ***4:33.6***  *R: No.*  *I: No? Are there any important questions from your perspective that are missing?*  *R: I don't think so. Flips pages no it seems to cover most of the aspects.*  *I: Good. And none of the wording seems confronting or…*  *R: …no…*  *I: ..or difficult?*  *R: No it’s pretty straightforward yeah.*  *Reference 1 - 6.14% Coverage*  *R: No, yes I do in I think that people that have you know like life threatening things need that extra little bit of support. If you’ve just got appendix that’s going to be better in a couple of weeks you get like me terminal cancer. It’s a big, big, big worry and I think more support, more support talking support you know would be probably a bit more handy. I know the services are there but to get into the services is very difficult.*  ***5:29.4***  *I: Okay so from your perspective just by what you said there, an important question which might be missing in that survey is around the support that you’ve received potentially?*  *R: yes, yes.*  *I: So and maybe going a bit specific into that support?*  *R: Yes*  *Reference 1 - 5.92% Coverage*  *R: No I …oh…I know probably sounds silly but I am aware I have a serious illness. I'm aware that people are aware that I have a serious illness and I'm not sure how much more emphasis I need to give it. You know this, my needs will be met through this survey just as much as, just because it doesn’t say and you’ve got a serious illness or I don't believe my needs are not being met.*  *I: Okay so you believe that from your perspective all the questions on that survey would cover your experience here in the hospital that they’re not missing anything?*  *R: If you wanted to go down to the like the end of the survey and then said now if you have a serious illness do you want to….you could then maybe involve a couple of palliative care questions. And that would be a way of, you know when you have a click here for more?*  *I: Yes, yes.*  *R: That could be a way of then…*  *I: Yeah branching off with the survey…*  *R: …incorporating some of that extra depth without necessarily making it necessary for everybody.*  *Reference 1 - 3.94% Coverage*  *R: Probably there’s different answers to like if you’re chronic I think there’d be different if it was just something normal they’d be different to what the chronic would be.*  *I: Okay. Do you think that there are questions that are potentially missing from a person with a serious chronic illness perspective?*  *R: I’m trying to think of one but I can’t think of one.*  *Reference 2 - 1.42% Coverage*  *I: You think that there is potential to be other questions that could be more important or more specific to that population?*  *R: Yes.*  *Reference 1 - 11.35% Coverage*  *R: Yeah ….I think you’re right it’s telling me about a point in time when I'm in hospital. And it probably doesn’t cover in regards to the progress with my chronic condition and it’s probably going to come up in a question later, I find with my chronic condition is that I'm doing these surveys but I'm not getting answers in regards to, and obviously there’s a reason for that. But every individual case is completely different. But I'm not getting answers of what I'm expecting next and that sort of can be frustrating yeah.*  *I: Okay with regards to that do you think there’s questions that are missing that you would like to see on that survey?*  *R: I would umm…*  *I: …can you think of anything like you said about your expectations would you like a question that sort of focuses on what the future might…*  *R: .,..okay yeah probably as a present situation and is possibly you know umm with myself like wanting to speak to someone about palliative care. And having that as an option in the questionnaire. Then they can come to you. I’ve got advice from people like I’ve got from the social person here that sort of gave me connections to palliative care. I'm sort of a little bit sort of shy a little bit sort of not up front in actually ringing up a person and saying “I want to speak to someone” but I prefer to be approached in regards to that sort of thing. It would be nice to have that as an option in a way. And that’s only just a slight example not a criticism and that sort of thing.*  *I: But it’s something like you would like to have that option?*  *R: Yeah, yeah and I know how busy you are and it’s a public service and I understand that. I know it’s not going to happen straight away but if I get on the list at least I can get someone to knock on the door at one stage.*  *I: Because your preference is that you would like them to approach you not having to approach them?*  ***6:55.7***  *R: Yeah, yeah.*  *Reference 1 - 2.76% Coverage*  *R: No I think it’s a good general one yeah.*  *I: It’s a good general one?*  *R: Yeah*  *I: Do you think that there’s any important questions that might be potentially missing from that perspective though? When it comes to a person living with a serious chronic illness?*  *R: Oh I’d have to think about that one a bit I think. Umm….pause…well it covers a very wide area of it anyhow doesn’t it? So I think it’s fair enough yeah.*  *Reference 1 - 2.27% Coverage*  *: Well I think it feels much more like a survey for people who aren’t necessarily having a lot of involvement. I think say for people like me you’d need another survey.*  ***13:12:3***  *I: Okay.*  *R: With more nuances in it and people are starting to get they’re starting to get used to and sick of nuances in those Covid reports constantly where journalists ask questions that pivot on one word or something. But I think also if it touches on your experience more well for me I can only speak for me even words like “needs” doesn’t quite sit with me I don’t know why.*  *Reference 1 - 12.49% Coverage*  *I: Yep. As I did mention it is not specifically designed like it’s designed for the general population so it’s not specifically designed for a person who may be living with a serious chronic illness. Do you think that matters at all?*  *R: Yeah.*  *I: It does matter. Why is that?*  *R: Because it’s not always chronic care that’s important it’s care in general.*  *I: Okay. So you believe care in general so it being a very general survey is a good thing or is there something missing from the perspective of a person with a serious chronic illness could be added in there?*  ***06:15:5***  *R: Oh yeah and in fact I think it should be.*  *I: So what should be so there should be something added in?*  *R: Yeah.*  *I: That’s specific to a person with serious chronic illness?*  *R: It should be yeah.*  *I: Can you think of anything that should be added from your prospective that you might feel just from your care journey thus far might be missing from that survey?*  *R: No.*  *Reference 1 - 3.71% Coverage*  *I: Do you think that that matters at all that it’s not designed for a person with serious chronic illness?*  *R: I’m not sure. I don’t I think it is.*  *I: Okay you think it is like it does cover all of the things even for a person with a serious chronic illness needs to worry about?*  *R: Yeah.*  *I: And their experience?*  *R: Yeah.*  *I: Okay so you don’t think there’s anything missing from the perspective of someone with a serious chronic illness?*  *R: I don’t think so. No.*  *Reference 1 - 5.35% Coverage*  *R: I think everyone’s going to be concerned about their care whether they’ve got a serious illness or not so I think all the questions are relevant.*  *I: Okay. You don’t think there’s any potential questions that are missing from the prospect of someone with a serious illness?*  *R: For people that it matters to you could possibly have questions in there about I don’t know compassionate care, end of life expectations and that sort of thing.*  *I: Okay.*  *R: Some people may or may not appreciate that. Whether or not the end-of-life expectations have been discussed with them that sort of thing.*  *Reference 1 - 1.57% Coverage*  *Yeah I feel probably an old hatter I don’t know if you’re ill you’re ill, you go to hospital no matter what part of the hospital you know just so long as you’re treated and your doctor that treated you in the beginning at Emergency or whatever you know follows through and gives you adequate treatment for yourself and whatever.*  *Reference 1 - 6.56% Coverage*  *R: Yeah chronic condition because there’s a whole lot of other stuff that comes in in terms of chronic conditions. I mean this covers off you know the higher-level stuff that you might experience but when you’re getting into chronic conditions there’s an expectation as in personally now that you want there’s an expectation about the knowledge I guess that your carers are having and all that sort of stuff.*  *I: Is there anything specific that you can sort of think of from your own experiences that you would or questions that you would like to see in there?*  *R: As a quick general survey no I don’t think so. It covers off pain relief, it covers off individual needs stuff. Probably it would be good to have something in there about cultural aspects. I mean while that could be under individual needs having something more specific about meeting your cultural needs is important particularly around indigenous populations. Actually that is actually an oversight in this I think.*  *Reference 1 - 0.72% Coverage*  *R: …if you have bad disease if something goes wrong and they stuff up you can really go down the shit tube can’t you?*  *Reference 2 - 2.30% Coverage*  *R: Umm well yeah there’s some different sorts of questions when your life (?) when you’re life’s…*  *I: …yeah and what would those questions …*  *R: …like they just give me a couple of months or something. Oh well a couple of weeks or whatever.*  *I: So what would those questions be can you think of any examples?*  *R: Well I sort of want to talk to a more professional person.* |
| **Preference for ConsideRATE** | *Reference 1 - 3.10% Coverage*  *I: So if I gave you both of these surveys and said okay because now you’ve had a chance to look at both of them and read both of them, which of those surveys would you prefer to complete given the choice of one?*  *R: Probably that one.*  *I: So the considerate?*  *R: Yeah*  *I: and why would you choose the considerate over the commission?*  *R: oh I don't know I just think it’s set up better.*  *R1: What questions may I ask, what questions?*  *R: Yeah doll it’s got like how would you rate our attention to your physical problems, things like pain, dry mouth, trouble breathing. And you’ve got very bad, bad, good, very good or doesn’t apply. So that’s the same to all the questions you have that choice of like very bad, bad, good, very yeah. And then you’ve also got, you can write extra on the back.*  *Reference 1 - 6.82% Coverage*  *R: I mean he’s been pretty good but he is very unwell and he’s quite tormented you know. They haven’t discussed this with him.*  *I: Okay.*  *R: And I know that he hasn’t got a long time to live.*  *I: Because of your experience?*  *R: Yes and I’ve seen him going downhill and I was talking to him this morning and I said look if you had a heart attack or if something that you want to be in there for you know do you want to come in and …*  *I: Resuscitate?*  *R: Resuscitate. Would you want him just to leave you? And he says no I don’t want them to resuscitate and that’s what I would like to discuss with someone. I don’t want him to do that.*  *Reference 2 - 4.26% Coverage*  *I: With regards to the survey itself if you were given the choice to complete one of the surveys which one would you prefer to choose?*  *R: This one.*  *I: So the Considerate?*  *R: Yeah.*  *I: Why would you take preference over the Considerate Tool?*  *R: I think it just I just find it more that it the other one’s quite general. This one tends to focus more on really the critically ill people.*  ***08:04:5***  *Reference 1 - 4.90% Coverage*  *So if I was to ask you to fill out either of the surveys which one would you prefer to fill out on behalf of [Patient name]?*  *R: This one.*  *I: The Considerate Tool. And why is that?*  *R: It really asks a lot more in depth as in like the first question you know “physical problems” “pain, dry mouth, trouble breathing” I mean it gives you …*  *I: It gives you that little bit of an example?*  ***09:03:4***  *R: An idea of what they’re asking.*  *I: And you find that’s important?*  *R: I do.*  *I: As a guide when answering questions?*  *R: Yes definitely. And like this “how are you feeling sad, worried or like a burden”. It’s all you know they are really important things that you’ve got to think about.*  *Reference 2 - 0.93% Coverage*  *R: No I like this one I think it’s concise.*  *I: So the Considerate.*  *R: Yeah I think it is. People are to the point on this one.*  *Reference 3 - 1.87% Coverage*  *R: This is really I think all those questions they cover pretty well everything that you have to think about.*  *I: And that’s what you’re referring to the Considerate Tool there?*  *R: Oh definitely yes. I definitely like the set up of that one a whole lot better.*  *Reference 1 - 1.54% Coverage*  *R: Umm…yeah I think the seven questions cover enough of the care plan that you would get in hospital. And the free text is a good thing to have. And the last one is fine as well.*  *Reference 1 - 0.89% Coverage*  *So I think the design of this is more user friendly definitely and for a person who’s perhaps in more pain than not this is definitely much more user friendly.*  *Reference 2 - 2.11% Coverage*  *R: Anyway I think this one’s much more user friendly.*  *I: So that’s the considerate you’re talking about?*  *R: Yeah the considerate.*  *I: Sorry I’m just because of the tape recorder sorry.*  *R: But it again if you could link you know comments the additional things you wanted to share to questions or suggest that people link them to the question if they wanted to that would be good.*  *Reference 1 - 4.74% Coverage*  *R: Oh this one here it’s more easy no they’re both good don’t get me wrong but this one here was better like as I said I had to read that sort of underneath the wording there and Mum’s very like I say very good it’s just not with breathing she has a bit of trouble her mouth’s not dry. She’s a bit more alert when she’s at home you know actually she’s better than me she can actually hear. I had the phone like the receiver was off the hook and I was going to go to the coffee shop and get some coffee. And I was in the kitchen and it was in the lounge room and she said what’s that sound. So I thought your hearing’s pretty good.*  *I: So because it’s an interview and we are getting this transcribed I’m just going to reiterate for the tape recording that you preferred the Considerate questions over the Commission tool questions if you were to fill out the survey.*  *R: Yes.*  *Reference 1 - 0.69% Coverage*  *R: I think it would be important to have those life expectancy that kind of stuff in it if it’s related because that is a really key part to a lot of our discussions is thinking about the future. Where the patient experience set one it doesn’t, there’s not a question that really edges towards that. Whereas the considerate one does it actually like it talks about you know sorting your affairs and those kind of things. So it’s kind of a, it’s a bit more specific.*  *Reference 2 - 2.24% Coverage*  *R3: Ahh probably prefer considerate mainly because I think if you had access to this at the time it could bring up some important conversations about things. Because you know patients are obviously very different in what they want to discuss. Sometimes they’d like to talk about time like to live or they want you know some certainty about that. And then there’s some patients who outright don't want to have to talk about it. And certainly things you know this had got specifics about wills, finances, advanced care directives and all of these things should be addressed while somebody is, particularly if they’re admitted to hospital. And if patients feel like they’re not then I’d actually see this as a really important tool at the time to start a discussion with them. Because it’s a short survey and you can actually see you know it’s one page you can actually see down the list and I don't know how the survey would be given. If it’s given as a confidential thing and then sort of tabulated but it could be useful as a tool at the time to start a discussion. Because if these things aren’t being met while they’re in hospital then you know there’s a couple of things here which are really, really, important I would think for patients. Like advanced care planning and like discussing their illness getting worse and they haven’t felt like they’ve been communicated about those things then I would see that one probably as a better review at a point in time. Particularly before they are leaving hospital.*  *Reference 3 - 1.13% Coverage*  *I: So if I'm hearing it correctly so far we’re thinking commission is good for broad high level information but the considerate tool could be used in clinical care deliveries? So you can give it out and then use that within the MDT to say the patient is worried about this, this and this.*  *R3 I think so.*  *I: Yeah.*  *R: I think that would give a quick snapshot yeah of what they’re thinking.*  *R3: Even as a pre-discharge tool or something like that to you know if they’re going to go home make sure that they’re aware of what’s going to happen and any of their needs are addressed. But that’s kind of, that’s probably why I prefer that one because I think this is good as a yeah as you say as an overall sort of picture of patient’s overall hospital experience.*  *Reference 4 - 0.92% Coverage*  *R2: I prefer considerate. Umm yeah I think that for all the same reasons there’s more specific sort of examples of things that the patient is almost prompted with to, rather than maybe from this one the commission where it feels a little bit more about like a broad general feeling. Of overall care and you know rather than pinpointing exactly what went really well, wrong, that’s given you all the patient that sense of umm like experience. This is …a little bit more specific to yeah like physical problems, what did we do well or not well, advanced care planning…*  *R: …yeah the considerate one is far more targeted ….*  *Reference 5 - 0.47% Coverage*  *R2: Yeah. Which is maybe what I was speaking to earlier if, we were, when we were saying that you know if we sort of did this it doesn’t really give us anything tangible to go on without having to drill down further. And maybe this one does a little bit more.*  *R: …it goes straight to the point…*  *R2: …directed. Yeah.*  *Reference 6 - 1.04% Coverage*  *R1: So I think I agree, I think this was also like a bit more user friendly.*  *I: The considerate tool?*  *R1: Yeah how it is displayed and I guess the specific information you get from here you can use. Whereas I don't know like if question four, I felt cared for, like if they circle …I guess you’d have to, you’d need them to elaborate more and be like “Okay well why did you not feel, why did you say never or why did you say rarely”? Whereas this is like quite specific to, it’s not as vague as this I guess I would say. Which is why I feel like it’s user friendly and it’s quicker and I think that’s what I feel like that’s how you get more numbers. If it’s something that’s quick and ticking a box.*  *Reference 7 - 1.31% Coverage*  *R3: From a clinical point of view this one.*  *I: Considerate? Yep.*  *R3: Yeah but for the reasons I mentioned before I think. You know some patients will not highlight you know their concerns about what’s happening and sometimes they’ll be more willing to do it in a form. But I think as well you have to think about who would help them fill out the form. Because if they’ve got you know medical staff or the people that have been caring for them and then they’re saying you know very bad for attention to their feelings. So maybe a little bit less willing to write that if the person caring for them is sitting in front of them. Particularly if there’s you know if the information is going to be used at a higher level and you know..for those sorts of things. So does it need to be someone independent or do they need to fill it out themselves? I don't know but that might be an issue.*  *Reference 8 - 0.27% Coverage*  *R: I think the considerate one could be something that would be done with a patient and family much easier than the other one. Because it might be more collective kind of questions.*  *Reference 9 - 1.65% Coverage*  *R: I think they have different, I think there’s some different benefits in each of them…*  *R4: ..it depends on what you’re aiming for…*  *R: …yeah in what you’re aiming for.*  *R4: Like in terms of the information that you are wanting to get I think considerate would be useful and you could implement it at whatever stage they are in. This would be easily accessible through an iPad like easier to tick than this kind of one. So it could be used easily through someone with an iPad. And I like the family being able to be involved without skewing too much with the ambiguity of the commission one. So the considerate would give you specific and user friendly things and I think it doesn’t like it’s pretty objective data versus subjective which you’re going to be getting from the commission based. I like this one.*  *I: You like considerate okay.*  *R: I think it’s got a few more palliative driven sort of questions.*  *I: Very much so.*  *R: Which from a palliative care point of view we would find of value yeah.*  *R4: This one would be good for every other patient that I have. That one would be better for my palliatives.*  *Reference 10 - 2.24% Coverage*  *: So you just mentioned question one to five of considerate you could more or less give out to anyone. So if you were to think of those first five questions as opposed to the twelve questions on the commission tool, as a bit of a screener for quality improvement would you have a preference?*  *R2: Definitely considerate.*  *I: Okay the first five?*  *R1: I think that I actually don't find now looking at both of them I don't find the commission one as helpful to really …I still feel the same. That if it was my umm choice of like if it was what we were using to screen people we’d still have to dig down further. Like it really doesn’t give me any tangible information to go with without having to pull the answers apart a little bit deeper.*  *R: It leaves us with more questions doesn’t it?*  *R1: Yep whereas this one I can the considerate like questions one to five for instance, you know it’s, they’re similarly framed about like how respected they felt and our attention to their feelings. But with the you know feeling sad or worried or like a burden might prompt them then to say you know the staff weren’t sensitive to how I was feeling. I did just get some bad news or feeling like a burden I hear a lot about. Just how busy they think the staff are all of the time and so you know like I think I feel like that would prompt them a lot more to…elaborate.*  *R: I think certainly one to five you could hand out, be done very quickly and I think you could do that to a very large population of the hospital.*  *Reference 1 - 1.06% Coverage*  *R4: I think it’s the wording that I think would confuse people rather than what’s really been asked.*  *R2: [name of researcher] having a look at both of them this one’s much more clinical.*  *I: The Commission’s much more clinical.*  *R2: Much more clinical and this one is much more to do with respect and dignity even though it’s busy I’d agree with that.*  ***27:06:6***  *I: So the Considerate Tool?*  *R2: I think this is more consideration of how they feel, was there respect, was there communication, good attention? This is all about your pain, was my pain relief adequate, was I confident that I was safe and I was you know confident in the treatment and care. This one’s more about how’s the hospital doing and how do you think the medical part was going. This one is much more looking at am I being treated with dignity and respect.*  *Reference 2 - 0.83% Coverage*  *R2: Well if a person doesn’t feel respected and this is what we’ve heard from here everywhere today if a person doesn’t have dignity, respect, communication, a feeling like your Mum that she can actually say something it’s not just being dished out to me. All of that is encompassed in more of the feelings at the am I feeling okay about how I’m being treated as a person comes through this.*  *I: Considerate?*  *R2: Rather than the clinical side of things.*  *R4: It’s more personal and it’s even you know my Mum if I asked her a question where you involved she wouldn’t ever have been sure if she was entitled to be involved because she was of that generation.*  *Reference 3 - 0.04% Coverage*  *R2: I think it’s more of the heart.*  *Reference 4 - 0.33% Coverage*  *R1: I feel it’s more person focused even though it’s got those things because if you’ve got physical problems feeling, surrounding, respect, communication those sort of things. It’s more open ended yes.*  *R3: That can make it hard to answer the question.*  *Reference 5 - 0.08% Coverage*  *So three are saying Considerate and two are saying Commission.*  *Reference 6 - 0.25% Coverage*  *R2: I’d love to hash the two because I like the way this one focuses on the individual how you felt, your perceptions, your perspective and this one’s more clinical which it has to be at some stage.*  *Reference 7 - 0.45% Coverage*  *R3: This one’s getting to know the person and this one’s purely about the medical experience.*  *R2: Yeah how did the medical experience go.*  *R3: Whereas this one’s getting to know the person which I guess could be beneficial for the long term.*  *R2: This one says did I feel valued, respected and a human being which is very important but it’s not everything.*  *Reference 1 - 0.06% Coverage*  *R2: I prefer the questions but not the layout.*  *Reference 2 - 0.89% Coverage*  *R1: I’d like to see sorry obviously I’m going to say I love this. But I would like to see how you’d line up that with that okay. It is messy but it’s actually attractive. It’s tricky if you’re having an off day but I like the way it’s asked you know the way it leads into the question.*  *R2: Just a bit of space in between.*  *R1: If you put that like the question there like that it would be easier. It’s definitely extremely attractive for someone wanting to fill it in it’s just going to be very jumbled. Someone’s going to go off track here but I like it better than I like the language here better.*  *R3: I like good, bad, better.*  *Reference 3 - 0.79% Coverage*  *R1: It’s the feeling of it.*  *R2: It’s more tangible.*  *R1: Like it’s a humanistic approach to something which I like. Our attention to your affairs, what matters to you, attention to your feelings. It’s all said in a similar it could be said here but it’s to me it’s not this is I don’t know however this is designed it doesn’t actually get to the heart of the matter that’s what I would say. So I absolutely love this I think if there was a way that you could get them to agree to just allow you to space it a bit to bring it down it just wouldn’t look so cluttered.*  *Reference 4 - 0.39% Coverage*  *R2: No in my opinion Considerate. I like the layout if we could space it a little bit more I really like the questions then for Questions 6 and 7 from my perspective I don’t have an issue with that but that’s from the cancer environment it’s different for the medical wards.*  *Reference 5 - 0.37% Coverage*  *R: System feedback whereas clearly this is more individualised and I guess to some degree would be more helpful from the point of view of physio-social type stuff. I guess some of them are going to be difficult about “attention to your affairs” or things like that.*  *Reference 6 - 0.34% Coverage*  *R: If for your project yes if it’s a systems thing and you want to know about you know the environment and some of the more I guess there’s sort of quality of care and then there’s perceptions of care and what you’re trying to measure really.*  *Reference 1 - 5.76% Coverage*  *I: So which survey would you prefer to fill out if you were given the option?*  *R: This one.*  *I: So the considerate.*  *R: Yeah*  *I: And why would that be?*  *R: Because I think that sort of gets you turn around and your care is not…what is important to you and that sometimes it’s not important to who is treating you. I get the feeling you know with nursing staff you know they’re to me they’re not all of them very considerate you know.*  *I: So do you feel this, the considerate tool might be a bit more personal than the…*  *R: …yes…*  *I: …commission tool? Is there anything else, are there any other reasons why you prefer it?*  *R: No I just…so easy to answer you know.*  *Reference 1 - 11.81% Coverage*  *R: pause Very good. Oh the first one how would you rate your attention to physical problems, things like pain, dry mouth and trouble breathing. Umm excellent, excellent. How do you rate your attention to your feelings, feelings like sad, worried or burdened, that’s excellent. Because as I said someone said that to someone this morning and we had a chat. So mmm. How would you …and they made me feel heaps better. How would you rate our attention to your surroundings, things like noise, light and more…well I got back into bed after going to the loo and the lady did the (shade?) just little things like that you know are just yeah just lovely. How would you rate or…oh how would you rate our respect for what matters to you? Perfect, very good. Umm how would you rate our communication about your plans oh just any time I asked a question answered immediately. There was nothing left out, I didn't want to know the gory bits but you know nothing was left out. Yeah I was fully informed all the way so yeah. Reads quickly to self….attention to your affairs, things like finances or advances, directives for care…yeah that’s fine that’s perfect, very good. How would you rate…sorry I normally wear glasses…*  *I: No, no, no.*  *R: How would you rate our attention to what you…*  *I: Do you have your glasses here? I don't see them on the table.*  *R: No they’re, we lived on a boat and the boat sunk and we lost everything.*  *I: Oh no.*  *R: Everything. How would you rate our attention to what you can expect like illness getting worse or time to live? Oh time left to live, umm…ahhh excellent.*  *Reference 1 - 5.44% Coverage*  *R: Oh yeah it’s all touchy that sort of stuff.*  *I: Is that touchy feeling?*  *R: How would you rate our attention to your surroundings? Oh I couldn't give a shit as long as they bloody fixed the pain that’s why. My overriding sort of…I don't care. I don't care if I'm in the bloody basement. The surroundings apart from this (?) you know …yeah. Anyway.*  *I: So if you were given the choice to complete either of those survey which survey would you prefer?*  *R: Oh the first one.*  *I: So you’d prefer the commission tool and why is that?*  *R: Well I think the first one is just got a bit more meat on the bone you know you can add a bit more meat . This one is a bit sort of tick a box. Tick and flick sort of touchy feely like I said.*  *I: Touchy feely.*  *Reference 1 - 2.81% Coverage*  *: Okay so if you were given the choice to fill out either of those surveys which one would you prefer to do?*  *R: This one.*  *I: The considerate tool. And why would you prefer to fill that one out?*  *R: It’s more in tune with me.*  ***13:43.7***  *I: okay yeah.*  *R: If that’s the right word.*  *I: Yeah, no it is yeah.*  *R: See I watch the TV and I learn these big words like this one here and…*  *I: ..so it reflects what …it reflects more about you?*  *R: …it reflects that they know what page I'm on love.*  *I: Okay yeah.*  *R: They know my needs, they all know me.*  *Reference 1 - 3.67% Coverage*  *I: So if you were given the choice to complete one of those surveys which one would you prefer?*  *R: Probably this one.*  *I: The Considerate Tool.*  *R: Yes.*  *I: And why is that?*  *R: I think it’s dealing a bit more with what’s involved with me than what this one is.*  *I: Than what the other one is?*  *R: Yes.*  *I: Than the Commission Tool.*  *R: Yeah.*  *Reference 1 - 0.59% Coverage*  *Ahh I suppose a bit more defining I suppose. Yeah. I can’t see a heck a lot of difference.*  *Reference 2 - 3.49% Coverage*  *R: Ahh…no I suppose this one is a bit more to the point isn’t it?*  *I: The considerate tool?*  *R: Yeah.*  *I: What do you mean by more to the point?*  *R: In respect …reading to himself…like you could go on for a month of Sundays about that one couldn't you?*  *I: You think question four is a bit too broad is that what you’re saying?*  ***18:46.7***  *R: Yeah how would you rate or respect what matters to you…reading…. Yeah you don't want to be pushing something down somebody’s throat because they’re your preferences sort of thing like.*  *Reference 1 - 3.30% Coverage*  *R: Oh this.*  *I: So the Considerate Survey?*  *R: Yeah.*  *I: And why is that?*  *R: Because it well partially it deals with first of all it deals with feelings and this one doesn’t feel that it deals with feelings even if somewhere it does and with things that are very difficult like “how would you rate our attention to what you can expect”. Well one of my answers to that would be not very well because they don’t like to you know I’ve had cancer now for two- and a-bit years really no one has ever told me how long I’ve got left. No one has ever even suggested that and still and I understand why they’re very careful with the therapies not to say you’re cured you know I’m in remission.*  *I: Yeah.*  *R: But yeah I think this one offers more space although oh there is a comments page right I was going to say I’d like a …*  *Reference 2 - 1.55% Coverage*  *R: Well let me tell you that I think the best question on this is “How would you rate our attention to your feelings”. Because I think that covers a whole range of stuff including to how delightful it is to come into a place like this which you think of as clinical etc, etc, and find people with senses of humour, people who are laughing, people who are generally just nice people.*  *Reference 3 - 2.70% Coverage*  *R: No because if you read this question if I was the carer and you were the patient and I read “how would you rate our attention to your physical problems” maybe instead of our it should say the hospital’s treat the hospital as a body.*  *I: Yeah.*  *R: Our I must admit even when I first saw it I wondered why it was “our” and that makes it and certainly if someone was reading the question to you and said “our attention” well you’re going to say oh if I say something negative it’s going to insult this person. So I think the “our” should go even if it’s not you know only the patient answering because it’s easier it is easier to be negative to a body of an organisation.*  *Reference 1 - 5.35% Coverage*  *I: So if I was to give you the option of completing one of those surveys which one would you prefer to complete?*  *R: The second one.*  *I: The second one so the Considerate Tool. And why is that?*  *R: Because I’m going through something like that.*  *I: Because you’re experiencing?*  *R: I’ve got cancer and I don’t have long.*  *I: So it relates more to you than the first survey so the Commission Survey?*  *R: Yep.*  *Reference 1 - 4.09% Coverage*  *R: Mmm this one easier.*  *I: Okay so the considerate. And why is that sorry? Because it’s easier?*  *R: Yeah it’s easier, everyone is friendly to me they’re all very good.*  *I: Okay. So you like that option of them actually giving you the prompts?*  *R: Yeah, yeah, yeah, yeah,….*  *I: …underneath the questions?*  *R: Yeah, yeah, it’s fine I think all very good.*  *I: Okay so are you also choosing the considerate because of the length? Because you just mentioned it was easier…*  *R: Yes it is easier yeah.*  *I: …so it’s not the three pages it’s just all like it’s one page.*  *R: Yeah but this one is really you can’t you know I like …all good. All medium all…*  *Reference 1 - 4.56% Coverage*  *R: Probably more Considerate.*  *I: Okay and why would that be?*  *R: Sorry?*  *I: Why would that be your preference?*  *R: Because I think everybody needs to have to be respected you know. We all need to there’s a lot of people out there in this world that don’t respect the elderly people I really do agree with that.*  *I: So do you believe that the Considerate Survey resonates better with you?*  *R: Probably with me yes.*  *I: Okay and is that because of the questions that it has? Is that because you prefer the style of formatting?*  *R: I think finance and affairs is very important.*  *Reference 1 - 5.08% Coverage*  *: In my own situation I’d probably do this one.*  *I: The Considerate Tool?*  *R: Yes.*  *I: And why is that?*  *R: Only because the most important issue with people in my and a lot of other people’s situations here is that you have a finite time to live and so at some stage you’re going to be needing to either discuss or come to grips with that. Which I think Question 7 is a very important one.*  *I: Okay.*  *R: Actually I was going to say before I would have added that question to this really.*  *I: Okay so you would have added Question 7 to the Commission Tool?*  *R: Yep.*  *Reference 1 - 1.23% Coverage*  *R: This is more personal.*  *I: That’s the considerate questions you believe are more personal?* |
| **Preference for Commission** | *Reference 1 - 1.20% Coverage*  *Umm…my only thought say like on question eleven, my harm or distress was discussed with me by staff. Maybe saying which staff might identify more of who is involved. Because often it is the front line nursing staff or, but it would be nice to know who is doing that more often if there was a you know if there was any form of altercation whatever, who is it that’s commonly doing the, that. Well that sort of discussion as such. And the only other thing I thought was on question eight received pain relief that met my needs. Certainly coming from a palliative care point of view pain is not the only thing. So whether we could broaden it to ‘were my symptoms such as’ because we get patients with chronic nausea and all sorts of other things. So pain is just like one snippet, whether we could broaden that?*  *Reference 2 - 1.14% Coverage*  *So overall would you say that you think even just this higher level screening brief tool would provide useful information for you at a ward level?*  *R: I think it would.*  *R1: Yes. I do.*  *R2: It’s very similar to what we already have the patient experience survey. And on oncology ward it’s that survey that feedback is really helpful. Particularly from like a quality and safety and even that’s, it helps us kind of figure out what we need to improve on. So because the questions are quite similar I do, it would be helpful but this obviously focuses more obviously the patient experience is the entire thing. Like food and like hand hygiene all that kind of stuff whereas this is more specific to like their own needs. Which yeah it would be it would help I think yeah.*  *Reference 1 - 0.36% Coverage*  *R1: Just physically this one’s easier to look at.*  *I: Okay the Commission is easier. I’m going to keep using the word for our recording.*  *R3: To engage me I found this one much easier.*  *I: The Commission layout is better.*  *R1: This one’s too busy.*  *I: So the Considerate is too busy.*  *Reference 2 - 0.17% Coverage*  *R3: In order to drive change I actually think it’s good for the medical profession to know directly the answers to these questions.*  *Reference 1 - 1.04% Coverage*  *I: There is but you’re wanting to capture their experience.*  *R1: But its retrospective isn’t it?*  *I: No this is real time.*  *R1: Real time but I was involved as much as I wanted. When a need could not be met staff explained why. It’s got a slightly retrospective feel to it.*  *I: Yes.*  *R1: When I was in the hospital I felt confident in the safety of my treatment and care.*  *R2: They must have been in for a few days.*  *R1: It feels a bit retrospective to me.*  *R3: And most of our patient experience is retrospective.*  *I: Yes.*  *R1: So I don’t know how you would get around that but that’s something [name] would have to …*  *I: So you could just change some my individual needs are …*  *R1: Are being met or something if you decide to go with this tool.*  *Reference 1 - 5.44% Coverage*  *R: Oh yeah it’s all touchy that sort of stuff.*  *I: Is that touchy feeling?*  *R: How would you rate our attention to your surroundings? Oh I couldn't give a shit as long as they bloody fixed the pain that’s why. My overriding sort of…I don't care. I don't care if I'm in the bloody basement. The surroundings apart from this (?) you know …yeah. Anyway.*  *I: So if you were given the choice to complete either of those survey which survey would you prefer?*  *R: Oh the first one.*  *I: So you’d prefer the commission tool and why is that?*  *R: Well I think the first one is just got a bit more meat on the bone you know you can add a bit more meat . This one is a bit sort of tick a box. Tick and flick sort of touchy feely like I said.*  *I: Touchy feely.*  *Reference 1 - 1.06% Coverage*  *I: Okay. So you prefer the formatting on the Commission Tool over the Considerate Tool?*  *R: Yeah it’s just cleaner.*  *Reference 1 - 1.50% Coverage*  *R: Well the way it is worded this one here’s just well realistically, well this one here is all about me about me that’s the way I look at it. But “how do I rate my attention to your surroundings” you’re asking me this you know but this one here’s saying “my views and concerns” so I prefer that one to that one.*  *Reference 1 - 0.72% Coverage*  *R: The first one.*  *I: The Commission Tool. And why is that?*  *R: It’s much easier to read and understand.*  *Reference 1 - 2.41% Coverage*  *R: …but I think the questions in this one…*  *I: ..in the commission tool…*  *R: …in the patient’s situation are better questions. Are more intelligent, more you know. Just better questions.*  *I: okay*  *R: More feeling in the questions more, my views and concerns were pursued, my individual needs were met and there’s more questions. They’re shorter and they’re smaller they’re more of them.*  *Reference 1 - 10.52% Coverage*  *R: On I don't like that sort of…I’ve seen things like this before and to me it’s just…I don't find it makes any sense to me anyway.*  *I: Yeah no so my question for you was going to be which survey would you prefer out of the two and why?*  *R: Number one because it’s more definite. This one seems to be you can take your pick.*  *I: Okay so the first one you prefer is this commission tool, so I’ve just because we are going to transcribe this so that’s why I'm just saying this. And the one that you feel is…what did you say it was sorry? It was not…*  *R: It’s just you could be….you could put something down there and it could be taken either way. In too many ways.*  *I: Okay and that’s the considerate tool that you’re talking about there.*  *R: Yeah I just don't find that, well it reminds me of oh I don't know some of these religious things or something. So you know then we can mark you down as you know.*  *I: Okay so you feel it’s putting a label on you as such or…*  *R: …yeah I think that yeah…*  *I: …when you’re talking about when you put yourself down as a specific religion how you follow a religion?*  *R: yeah it could be like that yeah. Yeah I don't know I just don't, I’ve seen things like this before and to me they don't make any sense.*  *Reference 2 - 0.98% Coverage*  *R: No that was good. Tried to find other words but I can’t find any other words that would be better used than that.* |
| **Prompts are helpful** | *Reference 1 - 4.90% Coverage*  *So if I was to ask you to fill out either of the surveys which one would you prefer to fill out on behalf of [Patient name]?*  *R: This one.*  *I: The Considerate Tool. And why is that?*  *R: It really asks a lot more in depth as in like the first question you know “physical problems” “pain, dry mouth, trouble breathing” I mean it gives you …*  *I: It gives you that little bit of an example?*  ***09:03:4***  *R: An idea of what they’re asking.*  *I: And you find that’s important?*  *R: I do.*  *I: As a guide when answering questions?*  *R: Yes definitely. And like this “how are you feeling sad, worried or like a burden”. It’s all you know they are really important things that you’ve got to think about.*  *Reference 1 - 1.06% Coverage*  *R: It kind of has that just that single small explanation of what the question is getting at is really useful I think. So that’s good people may still find some of this hard to understand.*  *Reference 1 - 0.78% Coverage*  *I: With regards to that it was more about the ease of the questions and you pointed to the explanations underneath the questions as well.*  *R: Yes.*  *Reference 2 - 5.97% Coverage*  *R: But this one here like that one there it was I had to go over the questions and I had to vision what the question was saying. I had vision myself and I had to vision Mum and I came back to that’s why I put always for that question.*  *I: And you were just pointing at the Commission tool then when you explained that. That’s just for the tape recording sorry. So is there anything unclear or confusing about the lay out?*  *R: No they’re both good don’t get me wrong they’re both good but this one here was a lot better so like I said I just read that part under the word.*  *I: So you preferred the lay out of the Considerate survey that they did that explanation underneath.*  *R: Yes.*  *I: You kept talking about that you had to envision the questions so having that sort of explanation underneath helped you in that?*  *R: Yeah then I could understand it.*  *I: Okay.*  *R: It’s just that with this one here I had to sort of put Mum in the picture and put myself in the picture as well and I had to like when she was doing stuff like the same again for this one here I put the same sorts of scenarios to this one.*  *Reference 1 - 0.37% Coverage*  *Umm…I think one page is always easier and I think that having the prompt of you know taking a bit of the ambiguity out of the question by then giving it, the patient the prompt afterwards of the types of things we’re looking for will help as well.*  *Reference 2 - 0.66% Coverage*  *R2: ..it’s messy and I even missed, when I first read it if I'm being honest I was like “Oh these questions I feel like are just going to like get a bit more of like a culture response”. In the sense of what’s the culture of this ward or place I'm in. But then when I looked closer and saw that the prompts were there for the types of information we’re trying to derive, that’s why I prefer it. But it’s definitely visually too busy and messy.*  *Reference 1 - 0.16% Coverage*  *R4: But the examples is a good thing about it because it puts their mind to what sort of things are they really asking about.*  *Reference 1 - 8.83% Coverage*  *R: So now here for example the first question is we are talking about rate our attention to your physical problems, things like pain, dry mouth or trouble breathing. Now there is no specific reference to those three or four conditions in this questionnaire. But I would say that it’s already been covered in this question.*  *I: Yeah. One thing I’ve grappled with in my head is I think in the commission tool it asks specifically about pain. And whether we were to shift the wording of the pain question to more broadly about symptoms? In the commission tool and whether that would be more helpful? Or do you think keep it at pain?*  *R: Yeah because pain is only one of the aspects. Now here we are talking multiple things.*  *I: In the considerate tool they give you that prompt about things like dry mouth or pain…*  *R: …yeah, yeah that’s what I'm saying.*  ***13:19.6***  *I: Yeah. Do you like the prompts, is that something that’s helpful?*  *R: Yeah I mean these prompts could be put in here as well.*  *Reference 1 - 1.04% Coverage*  *R: Umm that makes it a thousand times and your question underneath gives you what do you call it?*  *I: And example.*  *R: Examples, thank you.*  *Reference 1 - 0.89% Coverage*  *R: Well I think it’s a good idea because you’ve got a choice to have what you want.*  *Reference 1 - 4.09% Coverage*  *R: Mmm this one easier.*  *I: Okay so the considerate. And why is that sorry? Because it’s easier?*  *R: Yeah it’s easier, everyone is friendly to me they’re all very good.*  *I: Okay. So you like that option of them actually giving you the prompts?*  *R: Yeah, yeah, yeah, yeah,….*  *I: …underneath the questions?*  *R: Yeah, yeah, it’s fine I think all very good.*  *I: Okay so are you also choosing the considerate because of the length? Because you just mentioned it was easier…*  *R: Yes it is easier yeah.*  *I: …so it’s not the three pages it’s just all like it’s one page.*  *R: Yeah but this one is really you can’t you know I like …all good. All medium all…* |
| **Screening for ConsideRATE** | *Reference 1 - 1.03% Coverage*  *R4: But if you’re looking at the twelve month prognosis and they are discharging like you’re like not going there which isn’t necessarily the demographic we’re used to seeing. I think that’s what’s skewing like my vision on it specifically. Where I'm like most of our patients we maybe in the in-patient setting see them further like closer to the end date. Versus that twelve month prognosis. We like I'm respiratory predominantly so with chronic illness they’d be like you don't really know or they go into respiratory failure and they’re just not going to…*  ***44:16.7***  *R: ……it’s they are doing this like and there’s no…*  *R4: Yeah we don't often get like clear prognoses.*  *R: Yeah prognosis’s…*  *Reference 2 - 0.12% Coverage*  *R2: No I couldn't give this to everyone in my ward it would freak a lot of them out.*  *Reference 3 - 1.52% Coverage*  *I: Yeah so you’d have to, if we’re thinking process…*  *R2: ..it would have to be very specific…*  *I: …you’d have to screen….*  *R2: …on who I give it to. Yes.*  *R1: I probably would give it to any oncology patient because if they’re yeah….all laugh and talk*  *R: …it’s hard because coming from haematology I wouldn't be given that the same because a prognosis is very unclear because you’re aiming for cure. So you know like it’s not a downward palliative tree, it’s not a metastatic disease you’re actually trying to cure the disease. So they have a long process. So it’s not something that I’d give them at all. I think that’s , I think that, it’s important for them to think about the future but they’re targeting a curative intent. Yeah which is a bit different so I wouldn't be giving it to them.*  *R4: I would only give this to someone when palliative care is already involved in the care.*  *I: Would you?*  *R4: yeah I wouldn't have been giving it to someone who hasn’t had a palliative care because like this would scare them.*  *Reference 4 - 1.72% Coverage*  *R2: I only feel that question six and seven on considerate…*  *R1: …I was going to say that too…*  *R2: …you know are basically questions that would prevent me from giving it to my entire ward. I think that questions one to five is information that is useful for every single patient that I have. I just don't think they’ve all got the capacity or capability to fill it out. But this could you know inform all of like care for all of my patients. Six and seven is a little bit more framed towards people that have you know are aware of their chronic illness that isn’t going to get better. Which most of mine are also aware of that so it would still fit a lot of my cohort with general medicine and…*  *R: ….but there’s still a lot of the cohort under the kidney supportive care and stuff they’re like…*  *R2: Yeah like gen med and renal like they’re all chronically unwell and they’ve had that discussion at some point by the time they get to me. How much of that they have accepted is a different story. But it’s they definitely don't hit the ward without you know for the chronically ill people. Without knowing that they’ve got some sort of life long illness.*  *Reference 5 - 1.57% Coverage*  *R4: I think though this is the thing where it has to go through like who is the appropriate person to give this to, are they in their last year? You know like otherwise I couldn't give this to everyone because not everyone is in their last year in the ward. So I couldn't give this out as a…*  *R: …and it’s the same in cancer care too….*  *R4: …full yeah I can’t just give it to…*  *R: …. You couldn't…. you couldn't….*  *R4: …everyone but I could give it to anyone if that makes sense. But I don't think you would get everything that you were needing in a palliative sense. Because like I have dermatology on the ward they don't, like their skin condition is not…*  *R: …laughing no they’re not going to….*  *R4: They would answer the burden but they wouldn't, they would be like …all talking at once*  *R: I would be very surprised if the dermatologist has discussed anything to do with laughter… their prognosis.*  *R4: that’s why I'm like it wouldn't work for every single one of my patients but anyone who has got a year left would like this one. Just easier to read.*  *Reference 6 - 0.46% Coverage*  *R: Particularly that last sort of twelve months of life. Like that’s…*  *R4: I think twelve months is…*  *R: ..the last twelve months of life is a very broad yeah like that’s it’s almost impossible to really prognosticate like that. But anyone who you think has a significant (?) I think it’s very appropriate to use.* |
| **The need for unit specific PREMs** | *Reference 1 - 6.31% Coverage*  *R1: And it’s no reflection on the majority of the nurses it’s because they have to deal with dementia patients, they have to deal with mental health patients it’s a whole…at one point there was sixteen dementia patients in this ward waiting to go either home or to nursing facilities. And they are too busy running around chasing after them and meanwhile I’ve been buzzing for an hour one night, an hour. For a nurse and because they couldn't find one of the patients and I get that. But that’s what I mean by different, different wards are different experiences. So it’s, to do one survey as a reflection of the whole like the dialysis nurses and the ward over there is great. But to do one survey as a reflection of the whole hospital as itself isn’t going to work. You need to have one for emergency you need you know different but the…*  ***26:17.9***  *R: ..they’re all run different.*  *R1: They’re all run differently and they’re all…*  *R: ..and got different issues and different patients for different reasons you know what I mean?*  *I: Yeah*  *R: You know where you’ve got…*  *I: … I understand…*  *R: ….like you know people with cancer and you’ve got people like [patient] you know…*  *R1: But if they know you’re getting moved to a different ward or you’ve come from emergency and you were handed or your family member was handed ‘do you mind quickly filling this out’? and at the end of your stay they all got collected then that’s fine. But yeah different parts of the hospital are just run totally…different hospital …Prince Charles is only ten kilometres down the road but it’s run totally different. So different yeah.* |
| **The need to provide a voice for carers** | *Reference 1 - 5.17% Coverage*  *R: I think when I come in here to visit [patient] I’d just like to visit my daughter and leave here knowing she’s in good hands. I try and take a lot of the stress off the nurses, I wash [patient], I’ll change the (?) I’ll change the bed, I clean up, I help her with her meal. As a family we try and make sure that someone is here in the meal time. But because of lockdown they can’t come at night. So I'm at home stressing. And then [patient] rings me and she can’t open her butter or the jam. They won’t make her a piece of toast, it’s just I go home and I can’t even sleep.*  *I: Yeah. So you’re…*  *R: …it’s not right. The carers …somebody needs to care about the carers.*  *I: Yep.*  ***22:50.9***  *R: ..and the job they do.*  *I: And your experience is very important and we need to know what that experience is like.*  *R1: Yeah.*  *R: I should be able to leave her after being here for five or six hours a day I should be able to leave here knowing my daughter is safe you know. Every day I get home and it’s something….yesterday she came back from having a scan and they were putting her back on the bed and the big drip stand that was up here, the big steel drip stand came down and whacked her over the head. You know and she’d just come back from having a scan because of the bad headaches she’s been getting for two weeks.*  *Reference 2 - 2.61% Coverage*  *: …and this is you know …you know as a carer you try and do as much as you can to take the pressure off of people here. And when I'm here they’re really, the only thing they really do is like anything medical isn’t it [patient]?*  *R1: Mmm.*  *I: So…*  *R: …and I don't do that, I don't do that because they haven’t I'm not saying that. But as a mum the way I say this to people if I can do that little thing for [patient] when I'm here then I feel like I'm achieving something. It’s only something that I would normally be doing at home. So you know.*  *I: So you would like to be given the option of being able to fill out your own experience type of survey?*  *R: Yeah. Yep.*  *Reference 1 - 8.10% Coverage*  *: We added that third page which is the free text option. What do you think of that?*  *R: I think that’s a good idea.*  *I: Is that something that you would use?*  *R: Yeah I would most definitely because there’s been a few things that I have felt a bit in the dark about. Because we transferred from [name of hospital] and I do all his medications. He has a Webster pack so I know what he has and I add a couple of other things like Targin that the chemist prefers I put in and his Burinex which I put the one tablet in every day. And because I’m not here when the tablets are given out I worry that he’s getting his tablets and that wasn’t just here that was at Redcliffe as well. And when I ask questions it’s like I’m an what would you say untrained person if you know like me asking a nurse have you done this makes me feel like I’m being a bit pushy. But I like to be assured that he’s getting his blood pressure tablets and you know and I quite often say have you had your tablets.*  *I: So having that option to be able to write things down is important to you?*  *R: It is.*  *I: Because there’s things that might fall outside of the box.*  *Reference 2 - 5.29% Coverage*  *R: That would be good because I think carers have got a lot to put up with. That’s not a very nice way of saying it but you know what I mean.*  *I: It’s a big job.*  *R: It is a big job and you put your patient before yourself always you know and you get a bit worn out. You do get worn out and then you think I’m tired but …*  *I: So from a hospital perspective you believe it’s important for their carer’s experience to be like it should be valued as well?*  ***15:46:3***  *R: I think so. Yeah I think so because carer’s have got a lot of opinions and they know their person they’re looking after you know you can’t fault the hospital staff or the doctors they’re all just brilliant. But you know your person that you’re here for better than any of them.*  *Reference 1 - 7.50% Coverage*  *R: Umm…I guess the only real communication you get is when they’re looking at discharge. So you don't get any communication while they’re in there, it’s up to you to either get it through the patient or read the (?).*  *I: Yeah do you think there’s important questions that are missing from that perspective? So you spoke about the communication obviously being very sort of either limited or poor. So do you think there needs to be some questions surrounding that as such?*  *R: Well I guess just if there’s I guess like a brief summary of what’s happening with the care. I mean they’re seeing the patient daily I don't expect to get a daily update. But when the patient is unaware to communicate themselves it makes it a bit difficult to find out what level of care they’re actually getting. It’s something during their treatment not necessarily only getting involved on discharge.*  *Reference 2 - 6.19% Coverage*  *R: Umm…I think the considerate one probably needs to be more reflective for that sedentary caring rather than that primary one of the patient. So I guess the thing with the carers they’re more liaising with the rehabilitation team if you like to work out next steps and trying to put that in place. So maybe questions along that line I guess. Like do you feel that you’re included with the care plan of the patient? Yeah? Because sometimes umm maybe question five would cover that anyway. But I guess carers need to feel that they’re involved in the decisions of the patient as well not necessarily just thinking what we’re doing. So probably included within the plan itself I guess. Question five might even cover that anyway.*  *Reference 1 - 1.19% Coverage*  *R2: I think that would be helpful because carers are often advocating for someone who is vey sick and yeah I think it would be helpful. A similar set of questions but from point of view the carer’s point of view.*  *Reference 1 - 5.80% Coverage*  *R: No I think a bit of both like the patient and the carer as well it’s just I think at least the carer knows what’s involved you know what is involved with the patient and I think as the carer I like to know what I’m supposed to be doing. I know what I’m supposed to be doing but just to go that next level do you know what I mean go to that next step to improve my position as a carer. Just trying to go to that next one.*  *I: So you’d like to be able to complete a survey on that you can tell us your experience then?*  *R: Yeah like I said that everything’s sort of going well it’s just that and as a carer I’m really enjoying like I’m not working but I am working around the house so just doing all the physical stuff and trying to get Mum to relax a bit. So I am learning. I’ve never been a carer before so as I said it’s just a learning type of thing and I know if something happens to Mum I have to go back to the workforce because I’m not old enough to retire. And it’s just that I am looking forward to going back in the workforce because I’m putting on weight.*  *Reference 1 - 3.10% Coverage*  *R: And I think it should have been delved into a bit more. I mean I know he’s not the easiest patient in the world to get on with like for going to do things he puts them off. But yeah I think I suppose you’re dealing with different people you know. You’ve got him and then you’ve got another bloke who says oh this is what’s wrong with me you know can they make it quicker that they find out this things. I mean I’m going to lose him and I know there’s not one thing in this world that I can do to stop it.*  *I: So with that being said do you think there’s a question there that’s potentially missing around the communication side of things?*  *R: Yeah I do. I mean it’s not you can’t blame an organisation all the time but in his case yeah I can’t work that out.*  *Reference 2 - 1.21% Coverage*  *R: Well I mean you’re looking after somebody 24/7. They go to the hospital you’ve got to be there. I think that would be very good because it’s I mean the person’s sick and all that but you’re trying to take care of them and all that sort of thing. Yes you’re involved very much in their life.*  *Reference 1 - 2.80% Coverage*  *R1: Yeah I don't personally think that a carer could complete this one. Or I think you know especially when it’s framed at very my views and concerns, my needs. Like as much as they might still try to put themselves in the position of the consumer, if there’s you know….*  *R: …certainly, yeah….*  *R1: …that they’ll be sat there going “Well I know the buzzer has been going for ten minutes, and I asked to speak to the doctor yesterday and they haven’t called me back” you know so their opinion of what’s going on will be…*  *R: …yeah and certainly…,*  *R1: …which is not necessarily a bad thing…*  *R: …definitely from….*  *R1: ….but it’s not you know…*  *R: ….definitely from my experience the patient’s experience and descriptions compared to what their family’s description it can be…*  *R1: …totally different….*  *R: …very, very different.*  *I: Yeah*  *R1: Which is still like valid information but it won’t reflect the patient experience I don't think.*  *R: No. No.*  *R2: And also our demographic of patient on palliative pathway may not be able to answer this without assistance anyway because we have a lot of delirium involved which may…*  *R: ….particularly at end of life…that’s not going to be an easy thing to do at all.*  *I: Yeah*  *R2: Yeah, yeah.*  *R: That’s where obviously family would give us good feedback to what that is. But it would be missing the part of the patient obviously you know.*  *I: So if I'm hearing correctly you don't think doing it as a proxy would be helpful where the family member was trying to answer on the patient’s behalf. But you do think creating another tool that is specifically for carers and families could provide useful information?*  *R: I think it would provide us with a different set of information.*  *I: Yeah*  *R: Of their experience as on the sideline of their loved one. I think that would show us some very different information. Yeah*  *R2: Yeah I agree.*  *Reference 2 - 0.92% Coverage*  *I: So you’d do it together with the patient’s family or…*  *R: ..yeah or…*  *I: …you could do it with….*  *R: …like if they wanted to do it together. I think it would be easier with that one than…*  *I: Well interestingly the team that has developed considerate have said in there that actually it can be done by either. So they’ve written on the top people who are ill or their care givers. So…*  *R: Yeah and I think it would be easier to have a care giver do this one than…*  *R2: You would get more.*  *R: You would get more. Thank from the considerate one…the commission…*  *I: The commission one yeah.*  *R2: …patient centred one…*  *Reference 3 - 0.32% Coverage*  *R: So that might also give you more feedback instead of just a patient’s …palliative care point of view we want to see the family and patient as a whole. So that would be good information as a whole for us but yeah.*  *Reference 1 - 0.56% Coverage*  *R4: My initial thing is there is no way my mother and I couldn’t be here enough because Covid was happening so there where the times when I could be at there’s no way if I was helping my mother I could fill out that information and she certainly wasn’t able to. Whereas I could talk to her and maybe get a bit of a thing there but this is sort of like it’s …*  *I: So the Commission Tool you from a carer perspective feel you couldn’t answer it.*  *Reference 2 - 0.19% Coverage*  *R4: I feel this one. And I also like the idea it says people who are ill or their care givers you know whereas that’s not even mentioned on this form.*  *Reference 1 - 0.31% Coverage*  *R2: Only if you identify that it was the carer that filled it in and not the patient. Because the patient’s expectations may be very different from the carer’s expectations.*  *R1: And you’ve got all those family conflicts.*  *Reference 2 - 2.12% Coverage*  *I: Because what I’m wondering is though so not necessarily about the bereaved carers …*  *R1: No you’re measuring as you go along.*  *I: Well just if you were to come into a ward and I think all three wards actually would have several people who cognitively cannot complete it as a patient. Would it be valuable to you to have in those instances the nurse can say well actually I’ll give this to the carer and so you collect a carer data set and a patient data set around experience?*  *R1: But then you don’t find a lot of carers are in here a lot of the time.*  *R3: And then we’ve got Covid so no one’s here.*  *R1: So not scientifically but our current questions that we ask on our day-to-day kind of auditing are answered by the patient in the first instance but if they can’t answer it for whatever reason then they get the carer to do it.*  *I: Okay.*  *R1: So we ask the carer and we can differentiate that data if we need to so we do collect whether it was the but we do it as a proxy.*  *R3: That’s quite a good point.*  *R1: And we really just give the data as it is. I guess if someone was really thinking in their data oh this is because the carers are answering it all the time we could look into that data. We have the ability to do it but we don’t tend to we just tend to accept it as …*  *R3: What they’re experiencing.*  *R1: As a consumer experience being the patient and the carer.*  *I: Perfect.*  *R2: It’s presented as one?*  *R1: It’s presented as one we don’t differentiate when we present the data but we can if we need to.*  *Reference 3 - 1.69% Coverage*  *R: Yes I just said to [name] I think clearly the family or the carer in our area is a whole area that sort of potentially you know we don’t investigate too well. So one of our colleagues at QMR have just opened a study providing looking at support for carers in a pancreas cancer just for the carer side of things which was a follow on from a study we did here. But it’s always difficult you’re not really treating it’s hard to do things specifically apart from their feedback because they’re not actually patients of the hospital. But I think getting feedback from them in terms of how they feel about communication and other things would be very useful with the rider that families are complex and often different family members don’t agree with each other or the patient. So I was sort of saying to Elise having some sort of project in the future that sort of look at that in a little bit more depth which may have been done to some degree would be an interesting thing. Exactly how you do it I’m not quite sure and it’s not unique it’s universal not only here. The US oncologists talk about the daughter from Florida who flies in and ours used to be the daughter from Sydney or the son from Sydney.*  *Reference 1 - 3.06% Coverage*  *At the moment this tool has only been written for patients, I just wondered what you think about in relation to family members and carers, should we have something?*  *R: Yes you should.*  *I: Yeah? Okay and would you think something similar for them basically?*  *R: Yeah they need to be providing input about what they think about the care provided.*  *Reference 1 - 2.90% Coverage*  *R: Well I think there should be one for carers and one for patients because carers have different needs. You know they have different things that are not …I mean you’re looking for cure for the end of your problem. And I mean they’re just they’ve got to think well what can I do? I'm doing everything I can they need support as well.*  *Reference 1 - 2.54% Coverage*  *R: Umm…it would be a good idea because then you’re looking at two different perspectives. You know I might see it differently than what, because XXX is my carer, he might see it differently to what I do and he may give you a different answer than what I do. And that to me is looking through two people’s eyes you know not just the one person.*  *Reference 1 - 4.84% Coverage*  *R: Umm yes I do. If you want to differentiate yourself that’s possibly one of the things that worries me is how my husband is coping and the impact this is having on his life. Because at a certain point he’s going to have to go and find a new normal. And this is a new normal for him for now and then he’s going to have to go and find another new normal. And if I have a concern it’s not my needs being met because everybody is concerned whether I’ve got pain at seven or eight but …I need to …it would be reassuring to know that he had somewhere to go if he needed help. But we don't know how that looks yet because we haven’t really started the journey properly. We have to a certain degree because he’s been looking after me for weeks. But not this degree of intensity or that we know will be this degree of intensity.*  *Reference 1 - 3.46% Coverage*  *R: Well whose experience are you worried about? The patient or the carer? Umm I mean does it really matter what the carer….experiences?*  *I: I suppose there’s been some thought that carers are …provide and are there quite a lot throughout that journey for the patient. With the patient. So they might be able to provide some form of information.*  *R: Oh I think my partner would be of the opinion that she’s got better things to do.*  *I: Okay*  *R: and she’s my full time carer.*  *Reference 1 - 2.94% Coverage*  *I: So you would, you think it’s important for the carers to have a set of similar questions?*  *R: I do. I do. Yeah. And I’ve got very good carers love. Angela and Len they are very good.*  *I: Yeah and why do you think it’s important that the carers so why do you think it would important for your grandson and his wife to still be able to sort of ….*  *R: …well that way they sort of know a bit…*  *I: …let us know about their experiences?*  *R: ….what’s going on about me see.*  *I: Okay.*  *R: See somebody is communicating with them and letting them know how I'm going. You know.*  *Reference 1 - 1.84% Coverage*  *R: I don’t know that you need that. I tell my kids what’s happening and you know if they thought something wasn’t right I know especially my daughters would be saying so.*  *Reference 1 - 5.98% Coverage*  *R: On a voluntary basis as you know if they feel like doing it. I don't think it should be a you know something that the person needs to do, especially the carer or the …but umm I find those sort of things are beneficial to the person that’s helped, like the carer. Because they’re …I know for a fact they go through more in their worry and their concerns than what the patient does. The patient is lucky, they’ve got nurses and doctors taking care of them and all that sort of thing and they treat you really, really well. Whereby the poor old carer is sort of coming into the hospital, getting changes of clothes, making sure that I'm comfortable or the patient is comfortable and they’re sort of oh now I’ll just watch the patient get taken care of. And not the carer. And I understand the reasons for that but I think the involvement of a carer of their opinion is very important. Because of that, because they see things and hear things and probably got a lot more stress than what a patient does.*  *Reference 1 - 0.78% Coverage*  *R: Well it usually affects more than just the individual doesn’t it? It affects the whole family and the whole …so yeah.*  *Reference 1 - 3.46% Coverage*  *: It wouldn’t hurt.*  *I: And why do you say that?*  *R: Because they have a different perspective to the person who’s going through whatever they’re going through.*  *I: Okay.*  *R: My husband would do a dreadful job like I wouldn’t like him to try and fill that one out.*  *Reference 1 - 2.23% Coverage*  *R: I think carers they’re sort of left out. My partner was a carer for me and there’s not much info and people say that’s he’s a carer well okay and that’s all they seem to be but the carer is very important.*  *I: They are very important. They’re undervalued aren’t they.*  *R: Yes.*  *Reference 1 - 2.95% Coverage*  *R: Yeah possibly.*  *I: What do you mean by possibly? Do you think that matters?*  *R: Maybe something to give them an indication of what they can expect instead of just what the patient should expect.*  *I: Yep.*  *R: But that’s more education isn’t it? Education rather than information. Because it must be hard on them as well.*  *Reference 1 - 5.28% Coverage*  *R2: We know what they’re feeling.*  *R: We do we’re pretty well tele connected.*  *I: You’re in tune with one another?*  *R: Tele connected type of thing well we’re not but we are.*  *R2: Pathetically connected.*  *R: Pathetically.*  *I: So you believe that we should be having a set of similar questions for carers?*  *R: Yeah.*  *I: Okay.*  *R: Yeah why not you’ve got to ask the question as you say but there again you’ve got to have the people.*  *R2: And a lot of the time when they’re sick they’re not thinking straight.*  *R: They’re not thinking at the right level field no. I’ve been at home sometimes and I’ve been in bed you know just lying there you know just going through the motions and you know I couldn’t sit here with you doing this or lie there with you. I’m not being rude to you or anything but I’d end up telling you to move but that’s the way it is it’s just not right. But me this is as I said today is the best day I’ve had in a hell of a long time. Everything’s clear kind of you know well up to a degree I think. I’m getting better and I feel good.*  *I: Okay.*  *R: To do surveys type of thing.*  *Reference 1 - 1.96% Coverage*  *R: If the information you’re seeking is the same I would just go with the one tool.*  *I: Okay.*  *R: And just make sure that it’s clearly identified that it can be completed by either.*  *I: Yep.*  *R: I think that just helps keep the information that data cleaner but I think I’d be keeping it the same.* |
| **Would like a question about care on discharge** | *Reference 1 - 5.05% Coverage*  *R: It’s only just your after care that I always worry about.*  *I: Your after care?*  *R: Yes it doesn’t appear to be anything…*  *I: Okay so things about how you’re going to be cared for when you’re at home?*  *R: Yeah*  *I: …and so forth? Okay. So would you use the free text option to maybe write something about that in there about your concerns after your discharge?*  *R: Oh yeah.* |
