# Supplementary material for: Perspectives of inpatients with palliative care needs, their families, clinicians and key stakeholders on measuring quality of hospital care via patient experience measures: A qualitative study
Source: Palliat Med. 2023 Nov 3;37(10):1498–508. doi: 10.1177/02692163231209845 (PMC10657505; doi:10.1177/02692163231209845)
Supplement: sj-docx-2-pmj-10.1177_02692163231209845 – Supplemental material for Perspectives of inpatients with palliative care needs, their families, clinicians and key stakeholders on measuring quality of hospital care via patient experience measures: A qualitative study [file sj-docx-2-pmj-10.1177_02692163231209845.docx]

**Qualitative coding and theming_V5**

1. **PREMs for people with palliative care needs ought to be tailored to the needs of this population**
2. **PREMs should appraise whether the needs of carers have been met as distinct from those of patients**
3. **PREMs for inpatients with palliative care needs ought to be easy to use, brief and incorporate space for free text alongside each question.**
4. **Implementation of PREMs for people with palliative care needs ought to consider who administers these, when and how often**
5. **PREM data needs to be specific enough to inform process change and/or care provision**
6. **Patients and carers require meaningful feedback to encourage PREM completion**

| **Initial coding (inductive – within NVivo)** | **Related quotes** | **Descriptive theme V1** | **Descriptive theme V2** | **Analytical theme** |
| --- | --- | --- | --- | --- |
| **Formatting**  **Feedback about the icons**  **Brevity and ease of completion is important**  **Consistency and simple scale is important**  **Wanting an 'average' in ConsideRATE**  **Language and literacy**  **Prompts are helpful**  **Free text option is important** | **Formatting related quotes**  ***Space***  Patient 19  *I just find the formatting because of so close I do find it hard to sort of concentrate on that line and do the answer. More so than because of that, and that’s you know again from reading abilities and all that sort of thing.*  Consumer rep focus group  *R3: That’s what my thoughts were you’re more likely to engage with something simple rather than busy.*  ***Free text related quotes***  Carer 18  *Well I like that option that’s the question, that’s the answer, that’s it. But this one you’d have to go over the page and number it number one and then do something else and then go back over the page and number two, number three and they’re not there’s a disconnection between question and answer if you prefer to comment. Like if someone says number one is very bad you’ve got to go over the page to write why…* *If I felt strongly enough to write a comment I would want to do it in a way that it was clear cut and there’s no way anyone could misinterpret which question was linked to which answer I’d pick this one.*  Patient 12  *Honestly I look at it and go “Oh my God no, no I don't want to write an essay”…* *It puts me off even before I start reading the question…But I appreciate some people want to elaborate but I think the fact that there are so many lines means I think I have to fill it in.*  ***Icon related quotes***  Patient 10  *I think the symbols umm….as well as the questions are good because you can sort of look at it and know what it’s or partially know what it’s about before you even start reading… yeah you’ve got the question sort of half of that in your head yeah.*  Patient 12  *I don't know what these are for but they are very confusing…* *But I think the icons I thought the icons were going to somehow relate to the bad, good, very good and they don't. They are just pretty…Well for the reasons I mentioned I think this all looks very silly.*  ***Language / literacy related quotes***  Patient 20  *Again I’d just if I had a tertiary education I’d say if I was a patient with a tertiary education I’d say no but given that I’ve mixed with a lot of very ordinary people I’d say some of the language is too clinical.*  Consumer rep focus group  *R1: The words of some of those could be improved but I think the way it’s framed it suggests an openness or honesty in the response whereas this one even though I like the format of it better I think it’s more readable it does sort of pre-empt.*  *R3: You’re right because even in Question 1 it says “my views and concerns were listed to” so that’s sort of putting words in their mouth to agree.*  *R4: It gives you a positive statement.*  *R1: My mother never wanted to be a problem to anyone. She would have agreed.*  *R2: If you’re after proper data let’s look at these research finds if you’re after proper data then you’ve got to cater for all the cultural groups. So if we’ve got reading problems, if we’ve got cognitive problems, we’ve got language problems or even I was burnt you might have physical because my hands are bound up.*  *R3: There’s a lot of people that can’t read.*  *R4: Literacy let alone health literacy just normal literacy.*  Patient 26  *Yeah but I mean to say the way I write things and the way my brain goes around the corner it could be quite twisted but there again it couldn’t be it just depends on what I say and it’s what I do you know. Just what I said just before you know I probably went from down there up to here and then shot out there somewhere and come back again. It is what it is.*  ***Prompt related quotes***  Carer 6  *It kind of has that just that single small explanation of what the question is getting at is really useful I think.*  Consumer rep focus group  *But the examples is a good thing about it because it puts their mind to what sort of things are they really asking about.*  Patient 11  *R: Umm that makes it a thousand times and your question underneath gives you what do you call it?... Examples, thank you.*  ***Length related quotes***  Patient 1  *Nobody likes to fill forms. But this is twelve questions this is seven questions that’s the right number.*  Patient 12  *Can’t do it too short then you’re not going to get the depth of knowledge that you need. And I think most of us should be able to cope with twelve questions.*  Patient 20  *I don’t I’m in two minds I don’t think you necessarily need more but I think 10 questions would convince people of the sincerity of the survey.*  Carer 6  *So the seven is much easier to use I think. So I think from the perspective of the patient if it captures enough information than that’s probably better for them.*  ***Scale related quotes***  Carer 18  *I don’t think a hospital anywhere would have very bad or even bad. One of those two just bad would do…It would have to be an extreme for me.*  Carer 5  *… because I guess you’re just trying to associate a feeling with the care then rather than umm you know like a net promoter score, sort of rating. Some people would probably …it’s very hard to say something is very good or very bad I would say. So I don't know if you would get a good range of responses with that category. Whereas in the other one having always, sometimes, mostly probably covers the same sort of umm response that you’re wanting to get from them I guess.*  Patient 12  *Okay my first thing would be when I think of something as being very good that’s my number ten or five whatever it is…and that should be at the end of my row yeah? That should be my last thing because it’s the highest. And then I get doesn’t apply so that confuses me because I naturally go to tick this box and actually I need to tick this box ... If the doesn’t apply which has to sort of has to float at an end I think I’d go doesn’t apply, very bad, bad, good, very good you know. So doesn’t apply starts over here before very bad and we just shunt everything along.*  Carer 17  *I would like in between good and bad like average… Because I don’t like to jump from good to bad you know it doesn’t look good you know.* | **PREMs for inpatients with palliative care needs ought to be brief, well formatted and easy to complete**  Key areas:   - Formatting   - Space(poor formatting leads to disengagement)   - Free text -per question to capture stimulated thought for people with poor cognition   - Icons - Language / literacy:   - ensure language is not too clinical   - ensure the language doesn’t bias the response   - cognitive impact - Prompts – appreciated - Brevity – appreciated but also needs to balance being meaningful - Consistency with the scale + aspect of trying to have a feeling associated with care rating + wanting an average | **PREMs for inpatients with palliative care needs ought to be well formatted, easy to understand, brief and incorporate space for free text alongside each question.** | **PREMs for inpatients with palliative care needs ought to be easy to use, brief and incorporate space for free text alongside each question.** |
| **Perspectives on survey for all V for pal care**  **Preference for ConsideRATE**  **Preference for Commission** | ***The need for a more attuned survey tool***  Carer 17  *I think it just I just find it more that it the other one’s quite general. This one tends to focus more on really the critically ill people*  Patient 10  *I think that people that have you know like life threatening things need that extra little bit of support. If you’ve just got appendix that’s going to be better in a couple of weeks you get like me terminal cancer. It’s a big, big, big worry and I think more support, more support talking support you know would be probably a bit more handy. I know the services are there but to get into the services is very difficult.*  Patient 19  *it’s telling me about a point in time when I'm in hospital. And it probably doesn’t cover in regards to the progress with my chronic condition … I find with my chronic condition is that I'm doing these surveys but I'm not getting answers in regards to, and obviously there’s a reason for that. But every individual case is completely different. But I'm not getting answers of what I'm expecting next and that sort of can be frustrating yeah.*  Patient 20  *Well I think it feels much more like a survey for people who aren’t necessarily having a lot of involvement. I think say for people like me you’d need another survey.*  Clinician focus group  *I guess the specific information you get from here you can use. Whereas I don't know like if question four, I felt cared for, like if they circle …I guess you’d have to, you’d need them to elaborate more and be like “Okay well why did you not feel, why did you say never or why did you say rarely”? Whereas this is like quite specific to, it’s not as vague as this I guess I would say.*  ***General survey is ok but perhaps with ‘branching’ for depth***  Patient 12  *I know probably sounds silly but I am aware I have a serious illness. I'm aware that people are aware that I have a serious illness and I'm not sure how much more emphasis I need to give it. You know this, my needs will be met through this survey just as much as, just because it doesn’t say and you’ve got a serious illness or I don't believe my needs are not being met…* *If you wanted to go down to the like the end of the survey and then said now if you have a serious illness do you want to….you could then maybe involve a couple of palliative care questions. And that would be a way of, you know when you have a click here for more?*  *…incorporating some of that extra depth without necessarily making it necessary for everybody.*  Carer 18  *No not really for whatever reason you’re in hospital. Even if he was in here to have a baby no matter what it is if you’re in hospital, you’re being cared for the questions basically are the same. I don’t think serious illness comes into it.*  Patient 24  *I think everyone’s going to be concerned about their care whether they’ve got a serious illness or not so I think all the questions are relevant…For people that it matters to you could possibly have questions in there about I don’t know compassionate care, end of life expectations and that sort of thing.*  ***Reasons some preferred ConsideRATE questions – more aligned with needs***  Patient 15  *It’s more in tune with me… …it reflects that they know what page I'm on love.. they know my needs, they all know me.*  Patient 16  *I think it’s dealing a bit more with what’s involved with me than what this one is.*  Patient 24  *Only because the most important issue with people in my and a lot of other people’s situations here is that you have a finite time to live and so at some stage you’re going to be needing to either discuss or come to grips with that. Which I think Question 7 is a very important one.*  Carer 17  *I think it just I just find it more that it the other one’s quite general. This one tends to focus more on really the critically ill people.*  Carer 25  *It’s all you know they are really important things that you’ve got to think about…* *This is really I think all those questions they cover pretty well everything that you have to think about.*  Consumer Focus Group  *This one’s getting to know the person and this one’s purely about the medical experience.*  ***More aligned with key areas important for clinical care***  Clinician focus group  *I think it would be important to have those life expectancy that kind of stuff in it if it’s related because that is a really key part to a lot of our discussions is thinking about the future. Where the patient experience set one it doesn’t, there’s not a question that really edges towards that. Whereas the considerate one does it actually like it talks about you know sorting your affairs and those kind of things. So it’s kind of a, it’s a bit more specific.*  *there’s more specific sort of examples of things that the patient is almost prompted with to, rather than maybe from this one the commission where it feels a little bit more about like a broad general feeling. Of overall care and you know rather than pinpointing exactly what went really well, wrong, that’s given you all the patient that sense of umm like experience. This is …a little bit more specific to yeah like physical problems, what did we do well or not well, advanced care planning…*  ***Reasons some preferred Commission tool questions – less ‘touchy feely’ / preference for wording***  Patient 13  *Well I think the first one is just got a bit more meat on the bone you know you can add a bit more meat . This one is a bit sort of tick a box. Tick and flick sort of touchy feely like I said.*  Patient 26  *Well the way it is worded this one here’s just well realistically, well this one here is all about me about me that’s the way I look at it. But “how do I rate my attention to your surroundings” you’re asking me this you know but this one here’s saying “my views and concerns” so I prefer that one to that one.*  Patient 3  *More feeling in the questions more, my views and concerns were pursued, my individual needs were met and there’s more questions. They’re shorter and they’re smaller they’re more of them.* | **PREMs for people with palliative care needs ought to be tailored to their needs specifically**  **Key areas:**   - The need for a more attuned survey tool - General survey is ok but perhaps with ‘branching’ for depth - Reasons some preferred ConsideRATE questions – more aligned with needs - More aligned with key areas important for clinical care - Reasons some preferred Commission tool questions – less ‘touchy feely’ / preference for wording | **PREMs for people with palliative care needs ought to be tailored to their needs specifically** | **PREMs for people with palliative care needs ought to be tailored to the needs of this population** |
| **The need to provide a voice for carers**  **Complexity for carers to rate as proxy** | ***It is important to measure carers’ needs in addition to those of patients***  Carer 25  *It is a big job and you put your patient before yourself always you know and you get a bit worn out.*  Carer 6  *I think that would be helpful because carers are often advocating for someone who is vey sick and yeah I think it would be helpful. A similar set of questions but from point of view the carer’s point of view.*  Patient 1  *they need to be providing input about what they think about the care provided*  Patient 10  *Well I think there should be one for carers and one for patients because carers have different needs. You know they have different things that are not …I mean you’re looking for cure for the end of your problem. And I mean they’re just they’ve got to think well what can I do? I'm doing everything I can they need support as well.*  Patient 12  *If you want to differentiate yourself that’s possibly one of the things that worries me is how my husband is coping and the impact this is having on his life. Because at a certain point he’s going to have to go and find a new normal. And this is a new normal for him for now and then he’s going to have to go and find another new normal. And if I have a concern it’s not my needs being met because everybody is concerned whether I’ve got pain at seven or eight but …I need to …it would be reassuring to know that he had somewhere to go if he needed help. But we don't know how that looks yet because we haven’t really started the journey properly. We have to a certain degree because he’s been looking after me for weeks. But not this degree of intensity or that we know will be this degree of intensity.*  Patient 19  *On a voluntary basis as you know if they feel like doing it. I don't think it should be a you know something that the person needs to do, especially the carer or the …but umm I find those sort of things are beneficial to the person that’s helped, like the carer. Because they’re …I know for a fact they go through more in their worry and their concerns than what the patient does. The patient is lucky, they’ve got nurses and doctors taking care of them and all that sort of thing and they treat you really, really well. Whereby the poor old carer is sort of coming into the hospital, getting changes of clothes, making sure that I'm comfortable or the patient is comfortable and they’re sort of oh now I’ll just watch the patient get taken care of. And not the carer. And I understand the reasons for that but I think the involvement of a carer of their opinion is very important. Because of that, because they see things and hear things and probably got a lot more stress than what a patient does.*  ***The current tools requires a proxy rating and does not measure needs of carers***  Patient 12  *Whose perspective? Because you said that they’re completing it on behalf of their loved one. So it’s not them completing it, who is this for? It’s not for the caregiver, it says people who are ill or for their caregiver. Not it’s for this person who is ill but the caregiver is actually completing the form. So that’s very confusing.*  Patient 11  *because then you’re looking at two different perspectives. You know I might see it differently than what, because XXX is my carer, he might see it differently to what I do and he may give you a different answer than what I do. And that to me is looking through two people’s eyes you know not just the one person.*  Carer 9  *Well if these are about the patient what sense would it be me saying anything?...* *I’m looking at it through my eyes I’m not looking it through his eyes and that makes the difficult part of it you know.*  Carer 18  *No not really I think it’s probably more something that you’re here 24/7, I’m here a few hours a day. It’s more something that we could sit down and fill out together because he obviously knows a lot more about what’s going on in my absence.*  Clinician focus group  *I don't personally think that a carer could complete this one. Or I think you know especially when it’s framed at very my views and concerns, my needs. Like as much as they might still try to put themselves in the position of the consumer… definitely from my experience the patient’s experience and descriptions compared to what their family’s description it can be*  *very, very different.*  Exec Focus group  *Only if you identify that it was the carer that filled it in and not the patient. Because the patient’s expectations may be very different from the carer’s expectations.* | **Enabling carers to appraise care quality from their perspective and in line with their needs (rather than purely as a proxy rating of a patient’s needs) is required** |  | **PREMs should appraise whether the needs of carers have been met as distinct from those of patients** |
| **Happy to complete a PREM**  **Feels like criticising staff**  **Hard to be accurate**  **Subjective nature of PREM data**  **Need assistance to complete**  **Frequency for PREM completion**  **Give patients sufficient time to complete this**  **Paper V electronic completion**  **Screening for ConsideRATE**  **~~Confronting terminology~~ (too specific to appraising ConsideRATE specifically)?**  ***The need for unit specific PREMs***  ***Identifiers for patient groups*** | ***Good idea but be mindful of patient’s condition prior to providing survey***  Patient 1  *Depends on what condition I am in. Today I'm in a very good condition. When I came in on Thursday I was in a very, very bad state. So if someone was to come to me on that day I would have probably told them go away.*  Patient 13  *Well my reaction immediately would be “Hey I'm in pain I'm not going to be bothered filling out a survey”… Yes I didn't come into hospital to fill out paperwork.*  Patient 12  *I'd be fine, I understand that you’re…things are trying to get better. So this is a way, possibly, of finding out some of the information that you want in order to continually improve. Hopefully find gaps where if you’re not improving where aren’t you improving. That sort of thing, so I understand the reason for it.*  Patient 20  *I think you should be giving I don’t know how often but I know I would welcome this as a way to say thank you. But how often I don’t know .... I think it’s very important actually*  Patient 23  *Yeah there’s no reason why I wouldn’t want to complete it…Because I think it’s the only way you get through to solving problems.*  ***Timing***  Carer 5  *Because it needs to be taken not just once I guess you know so that there’s different parts of when you are an in-patient. There’s when you’re admitted, so when you’re admitted the answers might be different to when you’re moved to a ward and being treated to when you’re discharged. So if it was to be done to an in-patient the timing of the in patient’s road map would need to be considered in that as well.*  ***Need to carefully consider patient safety in providing feedback***  Consumer rep group  *I actually think it’s important to give direct feedback but I agree you’re not going to get that from patients unless they feel confident that they can be authentic when they answer this. I think that’s the big barrier…* *But I think patients need help to understand that it’s okay to be honest and they need help and support to be guided that they’re not going to be judged by what they’re saying and it’s not going to come back at them. It won’t affect their treatment but I don’t know that they feel that.*  Clinician focus group: *… you have to think about who would help them fill out the form. Because if they’ve got you know medical staff or the people that have been caring for them and then they’re saying you know very bad for attention to their feelings. So maybe a little bit less willing to write that if the person caring for them is sitting in front of them. Particularly if there’s you know if the information is going to be used at a higher level and you know..for those sorts of things. So does it need to be someone independent or do they need to fill it out themselves? I don't know but that might be an issue.*  ***Patient preparation needs consideration***  Consumer rep group  *The issue I have with just handing these out is I think patients need to be prepared. Communicated in some way about what these surveys mean because I don’t think my mother would have done this properly and she hadn’t lost capacity or anything.*  ***It can be hard to answer with specificity***  Patient 13  *There’s a lot of motherhood sort of statements you know that the sort of all encompassing…No my individual needs were met? Well some of them might have been some of them maybe not I don't know I mean I’d have to think about what you mean by individual needs were met*  Patient 2  *I suppose easy enough to fill out but a bit hard to be very accurate.*  Carer 6  *It’s like it’s hard to for people to separate out how the service they’re getting or whatever you want to call it in the hospital the care they’re getting in the hospital from their own pain and illness and those sorts of things.*  Patient 26  *I mean to say is “how would you rate your attention to your surroundings like things like noise, light and warmth” to me that’s stupid. A lot of people out there are pretty they don’t like a blind open. I love a blind open. It is what it is you know…* *Well everyone’s different you know it’s just a situation where it’s bloody hard.*  ***Most patients preferred to complete this in paper version although variance noted***  Patient 10  *Well paper. It’s more in my face to do it rather than umm a computer. Because a computer means nothing to me.*  Patient 19  *Oh I’d like to have an option but more than likely myself as a text or an email for myself personally. Obviously people, older people prefer paper but it’s easier for me to do it as a… (email)*  Patient 20  *It’s not so easy on a device especially if it’s a phone because they’re smaller and I have to tell you I never take surveys on the computer. I certainly wouldn’t on my phone.*  ***Some patients will need assistance***  Carer 6  *I think if someone had a serious illness they’d find it hard to engage with a survey period. I can’t think of some of the patients in Mum’s bay I don’t think any of them would be able to engage with this because they’re so sick.*  Patient 27  *Personally not necessarily but that’s obviously something that comes down to physical ability and all that sort of stuff.*  ***Frequency***  **At ward changes**  Carer 14  *If you’re an in-patient and you get changed to a different ward, I think it should be each ward you go to. Because different wards honestly run differently…*  **At key points within an admission**  Carer 17  *Close to the beginning, the middle and then like just before they leave.*  **At discharge**  Carer 18  *Probably a day or so before they leave maybe or depending on how long they’re here.*  Patient 21  *just once maybe just once on discharge*  **Every few months**  Carer 7  *Once every three months just to get to see how everything’s progressing*  Patient 15  *Every month is too much love. Six months and people would read them. But if you’re getting them every month let’s be honest you’re just chucking them aside. And it’s a waste of your time.*  **Within longer admissions**  Patient 11  *Probably each time I was in hospital.*  Patient 12  *I think there has to be a degree of discretion and smarts. Going into how often you give them the survey because at the moment we’re in a survey culture. Which is not helpful for people like you when I literally you know I phone Vodaphone and I’ve only just put down the phone. And they said “Please answer three questions survey”. I said “Well I don't know if we’ve actually done anything yet how can I answer your survey”? And it’s just it’s irritating. Please tell us about your experience in Myer this weekend? No, go away. You’re a shop I bought you did your job. So you need to be careful.*  Patient 16  *If you’re only in for a day or two you’re not going to know the answers to a lot of those questions. So I think more on long term ones I think..No I wouldn’t say everyone because some people come in and go home the next day you know.*  Patient 27  *a lot of that will depend on the particular circumstances of that inpatient and the timing of when it actually occurs during their stay. I mean for me like I’ve been here two weeks and I mean I could answer this relatively easily and comfortably but somebody who’s only been in for like two days may have a very different view because they’re not understanding how everything works. It’s almost like have you been in long enough to understand how the institution works. So for me I have a relatively good knowledge of how the health system works and all of that sort of stuff and what doctors and nurses are required to provide to you and things like that.* | **Routinely enabling patient’s with palliative care needs to appraise care quality is a good idea but who administers these, when and how often needs consideration** |  | **Implementation of PREMs for people with palliative care needs ought to consider who administers these, when and how often** |
| **Ability to use this data to inform change**  **Enabling PREMs to impact clinical care** | ***Specificity***  Clinician focus group: *… if it was a little bit more specific on you know were your needs addressed or whereabouts you know could we improve, you know directed a little bit more if that makes sense?*  *But then I think there would have to be a second screening, a point before we got to trying to implement some sort of project to know what we’re trying to target. So yes so even if this was just like a first step and then depending on specific areas that maybe have more problems than others or whatever doing a further drill down into what those issues actually look like to the patient.*  ***Clinical response***  *… I think if you had access to this at the time it could bring up some important conversations about things. Because you know patients are obviously very different in what they want to discuss. Sometimes they’d like to talk about time like to live or they want you know some certainty about that. And then there’s some patients who outright don't want to have to talk about it. And certainly things you know this had got specifics about wills, finances, advance care directives and all of these things should be addressed while somebody is, particularly if they’re admitted to hospital. And if patients feel like they’re not then I’d actually see this as a really important tool at the time to start a discussion with them. Because it’s a short survey and you can actually see you know it’s one page you can actually see down the list and I don't know how the survey would be given. If it’s given as a confidential thing and then sort of tabulated but it could be useful as a tool at the time to start a discussion. Because if these things aren’t being met while they’re in hospital then you know there’s a couple of things here which are really, really, important I would think for patients. Like advance care planning and like discussing their illness getting worse and they haven’t felt like they’ve been communicated about those things then I would see that one probably as a better review at a point in time. Particularly before they are leaving hospital.*  *Even as a pre-discharge tool or something like that to you know if they’re going to go home make sure that they’re aware of what’s going to happen and any of their needs are addressed.*  *I guess the specific information you get from here you can use. Whereas I don't know like if question four, I felt cared for… you’d need them to elaborate more and be like “Okay well why did you not feel, why did you say never or why did you say rarely”? Whereas this is like quite specific to, it’s not as vague as this I guess I would say. Which is why I feel like it’s user friendly and it’s quicker*  *So the considerate would give you specific and user friendly things and I think it doesn’t like it’s pretty objective data versus subjective which you’re going to be getting from the commission based. I like this one.*  *I don't find the commission one as helpful to really …I still feel the same. That if it was my umm choice of like if it was what we were using to screen people we’d still have to dig down further. Like it really doesn’t give me any tangible information to go with without having to pull the answers apart a little bit deeper.*  Exec and QI focus group  *I do worry when it’s collected if you don’t respond to it as well… why would you ask them if you’re not going to do anything about them sort of thing.*  ***Clinical response and information provision***  Patient 19  *you know umm with myself like wanting to speak to someone about palliative care. And having that as an option in the questionnaire. Then they can come to you. I’ve got advice from people like I’ve got from the social person here that sort of gave me connections to palliative care. I'm sort of a little bit sort of shy a little bit sort of not up front in actually ringing up a person and saying “I want to speak to someone” but I prefer to be approached in regards to that sort of thing. It would be nice to have that as an option in a way.*  Patient 19  *…I think keeping it under sort of twenty is probably a good idea even under fifteen is a good idea. But as I said to you before on both these questionnaires I’d like to get, instead of writing that information out, getting more details. And more generalised like I said you know would you want any more assistance from palliative care from dieticians etc. Just as a tick box than writing it down because as I said you don't know what’s available through Queensland Health and all that sort of thing. I didn't know a thing about palliative care until recently and I’ve been in the system for four years.*  Carer 6  *It would be good to be able to express that to the staff who could actually do something about that and it might be for a really specific thing you know like my heel was rubbing. Like a guy in Mum’s other ward you know just his heel was rubbing on the bed a certain way that was painful or whatever and he carried on for a really long time and then that was fixed it was so easy.*  ***Addressing complex concerns***  Exec and QI focus group  *And even though those questions are difficult for some of the young ones to ask like you know you might bring up we have issues all of the time with our complex patients about finances and that. The social worker then sort of deals with those.* | **PREM data needs to provide enough specificity for clinical teams to utilise**  3 key areas:   1. Specificity 2. Immediate clinical response re clinical improvement 3. Focus on complex areas is helpful | **PREM data needs to be specific enough to inform process change** | **PREM data needs to be specific enough to inform process change and/or care provision** |
| **Patients need to feel this information is for a reason** | Carer 14  *…because those forms are probably getting not filled out because people with chronic care chronic illness like myself either are too sick to do it or can’t be bothered. Because your voice isn’t heard.. what’s the point, it never has changed it won’t change.*  *Carer 14*  *I would be happy to put my name to it and not be anonymous if I knew that I was going to get a text message…and even if you tick the feedback you never get the feedback.*  Carer 14  *Like I’d like to know once the survey is all done what is going to be the action after that, like what is the process after that? Is it just done and then just swept under the carpet onto somebody’s desk and then it’s just forgotten?*  Patient 13  *Well I’d like to…get some sort of ahh responses to what actually was the result of the survey. So that you’re not just ticking boxes or making comments and they’re going into some deep dark hole somewhere… Just so that you say ‘oh well I completed that survey I was involved in that survey and this is what happened’.*  Patient 13  *I would like to think if I’ve devoted half an hour or an hour to filling out a survey or whatever that I would get some sort of return feedback to say well this is what resulted from participance in this survey. This is the actions that we’re taking or this is what people didn't agree with or whatever you know … You know and with all this sort of end of life stuff you’re talking about people that really haven’t got a lot of time to spare. So we don't want to be spending hours and hours bogged down on paperwork. But I mean I would agree with trying to help somebody in the future if we can improve the services. But I'm not going to fill out a survey if it’s just going to be thrown in a too hard basket somewhere.*  Patient 19  *feedback is not so much sort of thank you for giving us a good mark or a bad mark and just you know we’ve taken account of your response. And we’ll keep in touch with regards to how it’s progressing or something like that.* | **It is important to provide a meaningful response to patient feedback**   - Understand process of action in response to feedback provided - meaningful feedback about system improvement work + specific aspect of EOL care and not wasting time | **It is important to provide a meaningful response to patient feedback at the individual level** | **~~Patients and carers require meaningful feedback in response to survey completion to feel this is worthwhile~~**  **Patients and carers require meaningful feedback to encourage PREM completion.** |
